# Synaptonemal Complex in Human Biology and Disease

**DOI:** 10.3390/cells12131718

**Published:** 2023-06-25

**Authors:** Elena Llano, Alberto M. Pendás

**Affiliations:** 1 Departamento Fisiología y Farmacología, Universidad de Salamanca, 37007 Salamanca, Spain; 2 Molecular Mechanisms Program, Centro de Investigación del Cáncer, Instituto de Biologıía Molecular y Celular del Cáncer, CSIC-Universidad de Salamanca, 37007 Salamanca, Spain; amp@usal.es

**Keywords:** synaptonemal complex, meiosis, meiotic recombination, infertility, cancer

## Abstract

The synaptonemal complex (SC) is a meiosis-specific multiprotein complex that forms between homologous chromosomes during prophase of meiosis I. Upon assembly, the SC mediates the synapses of the homologous chromosomes, leading to the formation of bivalents, and physically supports the formation of programmed double-strand breaks (DSBs) and their subsequent repair and maturation into crossovers (COs), which are essential for genome haploidization. Defects in the assembly of the SC or in the function of the associated meiotic recombination machinery can lead to meiotic arrest and human infertility. The majority of proteins and complexes involved in these processes are exclusively expressed during meiosis or harbor meiosis-specific subunits, although some have dual functions in somatic DNA repair and meiosis. Consistent with their functions, aberrant expression and malfunctioning of these genes have been associated with cancer development. In this review, we focus on the significance of the SC and their meiotic-associated proteins in human fertility, as well as how human genetic variants encoding for these proteins affect the meiotic process and contribute to infertility and cancer development.

## 1. Introduction

Meiosis is a fundamental cellular process essential for sexual reproduction, which reduces the genome by half, generating haploid gametes (sperm and eggs) from diploid cells [1]. This process involves a series of events unique to meiosis, including pairing, synapsis, crossover formation between homologous chromosomes, suppression of sister centromere separation during the first division (reductional), and sister chromatid separation during the second division (equational). Notably, meiosis displays an extended prophase I, during which many of these characteristic events occur, such as the formation of programmed double-strand breaks (DSBs) and their repair via homologous recombination (HR), as well as homologous chromosome pairing and synapsis to form bivalents. Resolution of these DSBs leads to a subset of recombination events that mature into crossovers (COs). These COs are essential for maintaining physical binding (chiasmata) between homologues until anaphase I, ensuring accurate segregation during the first meiotic division. COs contribute to genetic diversity through two mechanisms: chromosome assortment and genetic recombination [2]. The synaptonemal complex (SC) is a meiosis-specific megaprotein structure conserved among sexually reproducing organisms that forms between homologous chromosomes during prophase of meiosis I. The SC mediates and maintains synapsis along the full length of each pair of homologous chromosomes, facilitates the exchange of genetic material between them, and sustains CO formation which is essential for the proper segregation of chromosomes at the first meiotic division.

Thus, SC assembly is crucial for successful meiosis progression [1,3]. Errors in any of these events can lead to chromosomal missegregation during meiosis, resulting in miscarriages, infertility, birth defects, and tumorigenesis in humans [4]. In this review, we will examine the structure and function of the SC, including the proteins directly associated with its structure, such as cohesins, and their roles in SC assembly and function. Additionally, we will explore the function of the meiotic recombination machinery associated with the SC and the impact of human genetic variants encoding these proteins on the meiotic process, as well as their contributions to infertility and cancer development.

## 2. Structure and Function of the Synaptonemal Complex

**Figure 1:**
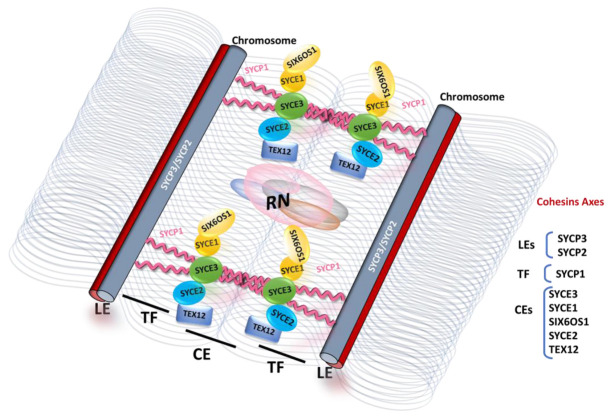
Schematic representation of the synaptonemal complex (SC) based on recent interaction data between central element proteins and the transverse filament [25]. The SC assembly, facilitated by the central element (CE) region, provides a necessary three-dimensional framework for double-strand break (DSB) repair and crossover (CO) formation. This assembly process is directed by recombination intermediates that are enzymatically processed by dynamic macromolecular protein complexes called recombination nodules (RNs). The transverse filament (depicted in pink) is composed of a supramolecular SYCP1 tetramer lattice that binds parallel SYCP1 dimers together. It supports cooperative head-to-head interactions between the N-terminal interaction protein sites of bioriented SYCP1 tetramers, which are anchored to chromosome axes through the back-to-back assembly of their helical C-termini [26]. Strong interactions between central element proteins are depicted by the overlap between the ovals representing SYCE2–TEX12 and SYCE1–SIX6OS1. Weak interactions are shown as punctual contacts, with SYCE2 interacting with SYCE3 and SYCE1 interacting with SYCE3. The grey coiled lines represent DNA loops generated by the meiotic cohesin axes.

The discovery of the SC in primary spermatocytes of invertebrates and vertebrates was first reported by Moses and Fawcett in 1956 [5,6]. The ultrastructure of this complex has been found to be remarkably conserved among sexually reproducing organisms, indicating a fundamental and conserved role in meiosis [7,8]. The SC comprises a tripartite structure consisting of two parallel lateral elements (LEs), which are referred to as axial elements (AEs) prior to the loading of central region proteins, and a central region (CR) containing the central element (CE) and the transverse filaments (TFs) (refer to Figure 1).

While the SC proteins are highly divergent in different eukaryotic phyla and cannot be identified across distant taxa based on sequence homology, their ultrastructure is extremely conserved. The TFs are arranged head-to-head, with their N-terminal domains situated in the middle of the CR and their C-terminal tails facing the chromosome axis within the LEs. The CE proteins overlap with the N-terminal domains of the TFs in the middle of the CR, providing stability to the SC structure. Furthermore, most of the CR proteins described in different organisms contain coiled-coil domains, which promote protein–protein interactions and enable homodimerization [9,10,11]. These conserved features are potentially critical for the biological functions of the SC, although the underlying mechanisms remain unclear.

The AEs of the SC assemble with cohesin complexes along the chromosome pairs to form the chromosome axis and maintain cohesion between sister chromatids, which promotes sister kinetochore mono-orientation and avoids chiasma slippage off the chromosomes [12]. 

Before SC assembly in its tripartite structure, each chromosome must identify and pair with its homologue to prevent SC assembly between nonhomologous chromosomes [13,14]. The process of SC assembly is precisely regulated and begins during leptotene with the formation of protein axes known as AEs along each chromosome. During zygotene, the AEs become increasingly continuous, closely aligned and connected through the CR. Once synapsis occurs, the AEs are referred to as LEs of the SC. The CE plays a crucial role in promoting synapsis between homologous chromosomes and facilitating their genetic exchange. Structurally, the TFs cross-link the LEs in a zipper-like manner, and together with the CE, they form the CR of the mature SC. At pachetene, the SC assembly is completed, synapse is achieved, and crossing over occurs. Consequently, the SC holds homologous chromosome pairs in synapsis throughout prophase I, from zygotene to pachytene [15]. Subsequently, during the diplotene and diakinesis stages, the SC disassembles, while the homologs desynapse but remain connected through chiasmata until the chromosomes segregate at anaphase I and the recombination process is completed.

In mammals, including humans, the process of synapsis differs between males and females due to the distinctive nature of sex chromosomes and the presence of different sex-determining mechanisms. During synapsis in males, the XY sex chromosomes pair only at specific small regions called pseudoautosomal regions (PARs), allowing for pairing and recombination, while the nonhomologous regions of the sex chromosomes remain unpaired. In contrast, in females, the homologous X chromosomes extensively pair throughout their entire length, facilitating recombination and the exchange of genetic material between the homologous X chromosomes. These differences have dramatic consequences in the meiotic arrest of spermatocytes when synapsis is incomplete, as compared to the more permissive control of synapsis in females [16].

In mammals, eight SC proteins have been identified: SYCP2 and SYCP3 (synaptonemal complex protein 2 and 3), located in the AEs/LEs; SYCP1, present in the TFs; and SYCE1/2/3 (synaptonemal complex central element proteins 1 to 3), TEX12 (testis-expressed protein 12), and SIX6OS1, forming the CE [17,18,19,20,21,22,23] (Figure 1).

Although there is no sequence homology between the CE proteins of the SC across different species beyond the basal-branching phylum of Cnidaria, multiple lines of evidence suggest a common evolutionary origin for these proteins [8]. Notably, the axis/core proteins, including Red1 in budding yeast, SYCP2/SYCP3 in mammals, and ASY3/ASY4 in plants, share an evolutionarily conserved structure. These proteins form homotetrameric or heterotetrameric coiled-coil assemblies that further oligomerize into filaments, which play crucial roles in meiotic chromosome organization and recombination. Additionally, the closure motifs in each complex recruit meiotic HORMADs, the master regulators of meiotic recombination, in these distant species [24]. Although some components of the SC and meiotic chromosome axis have undergone dynamic evolution, these findings highlight the significance of these proteins in meiosis and provide insights into the evolution of these structures across eukarya.

## 3. Assembly of the SC in Mammals

The AE components responsible for the organization of meiotic chromosomes in prophase I include, in addition to the two AE proteins SYCP2 and SYCP3, the somatic and meiosis-specific cohesin subunits (SMC1α and SMC1β, SMC3, STAG3, RAD21, RAD21L, REC8) and proteins belonging to the meiotic HORMAD family (HORMAD1 and HORMAD2) [24,27].

The first step for the assembly of AEs is the loading of the meiosis-specific cohesin complex at meiotic onset, which provides an evolutionary conserved scaffold for the recruitment of other meiosis-specific proteins whose incorporation into AEs is required for subsequent meiotic events.

**Figure 2:**
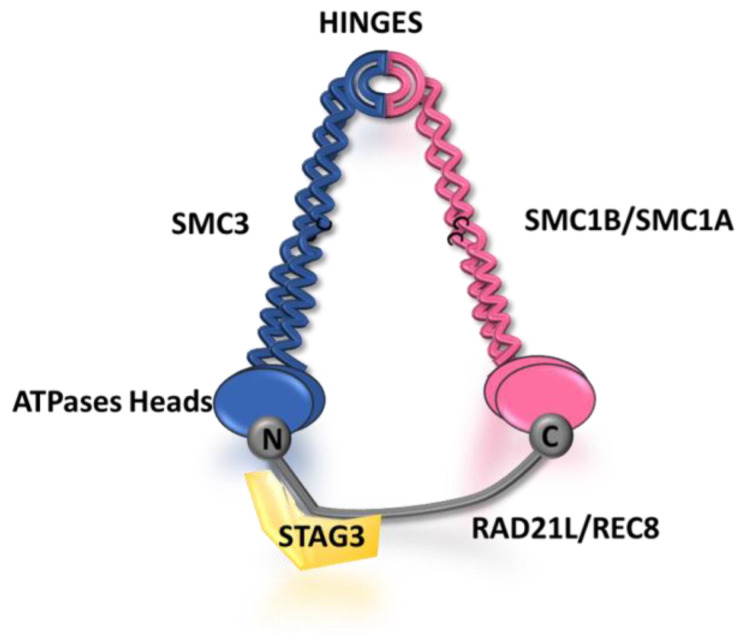
Schematic representation of the male and female meiotic cohesin complex according to the ring model. Meiotic cohesins are composed, both in males and females, of two structural maintenance of chromosomes (SMC) subunits (SMC1A or SMC1B and SMC3), as well as an α-kleisin subunit (Rec8 or RAD21L) and a STAG3 subunit. The SMC subunits contain hinges and ATPase heads which allow them to interact with each other and with the N-terminal and C-terminal domains of the α-kleisin subunit, respectively. The STAG3 subunit is known to interact with REC8/RAD21L and is indeed essential for the integrity of the ring complex. It is believed that cohesins, similar to their activity in somatic cells, generate DNA loops at the synaptonemal complex through DNA extrusion, facilitating proper chromosome organization during meiosis.

The cohesin complex is a proteinaceous structure capable of DNA looping, either between two sister chromatids (trans) or within the same DNA molecule/chromosome (cis), through its ring structure [28]. Cohesins play a critical role in chromosome segregation, DNA damage repair, gene regulation, genome organization, and also synaptonemal complex (SC) formation [29]. The founding function of cohesins was discovered in yeast, owing to their critical role in maintaining the cohesion of sister chromatids during cell division, which is indispensable for the accurate segregation of chromosomes [30]. The somatic cohesin complex is composed of four main subunits: two structural maintenance of chromosomes subunits (SMC3 and SMC1α), a stromal antigen protein subunit (STAG1 or STAG2), and one α-kleisin (RAD21). There are four meiosis-specific cohesins, which include one SMC protein, SMC1β; two kleisins, RAD21L and REC8; and a stromal antigen protein, STAG3. All mammalian meiotic cohesin complexes contain the subunit SMC3 with SMC1α or SMC1β, the STAG3 subunit, and one of the three kleisins RAD21, REC8, or RAD21L, leading to a wide variety of meiosis-specific cohesin complexes [28] (Figure 2). 

Consequently, it is widely accepted that the kleisin subunits confer distinct specificities to the cohesin complexes formed during meiosis. In meiosis, the cohesin complexes play a crucial role not only in meiotic DNA recombination and DSB formation but also in the proper organization of the AEs and chromosome synapsis (Figure 2). Additionally, cohesins are necessary for AE assembly, and this function is evolutionarily conserved from yeast to mammals [31,32,33]. LE proteins assemble alongside cohesins and form the chromosome axis by establishing the loop-axis meiotic chromatin structure [11]. The recruitment of cohesins to the chromosome arms is tightly regulated and occurs in a sequential manner, with different cohesin complexes being recruited at different stages of meiotic prophase I. 

After the premeiotic S phase, it is thought that sister chromatid cohesion is established through the formation of the α-kleisin Rec8 complex. During early leptotene, the RAD21L-containing cohesins play a crucial role in clustering the pericentromeric heterochromatin, which is essential for the formation of the bouquet structure and homologous pairing [32,34]. At the onset of anaphase I, the Rec8 cohesin complexes undergo dissociation from the chromosomes due to the action of separase, a cysteine endoprotease that cleaves the kleisin subunit of cohesin. As a result, the homologous chromosomes separate. However, it is important to note that at this stage, the centromeric Rec8 is shielded from degradation by Sgol2 [35]. This protective mechanism allows the sister chromatids to maintain their association at the centromere throughout the first meiotic division. In metaphase I, REC8 levels increase at the centromeres, where it remains bound until cleavage at metaphase II by separase [36,37,38]. Although RAD21L is also associated with the centromeres from metaphase I until anaphase II [39], it has not been formally demonstrated whether it undergoes separase-mediated cleavage.

RAD21L and REC8 are crucial for the association of the axis-associated proteins, although they contribute differently to the formation of the AEs [39,40]. The absence of AE formation in double mice mutants lacking both Rec8 and Rad21l further demonstrates the synergistic action of these kleisins [32].

While Rec8-deficient spermatocytes lack synapsis between homologues, they still exhibit synapsis between sister chromatids with SYCP1 labelling, and a substantial population of spermatocytes still shows homologue pairing. Conversely, Rad21l knockout results in impaired homologue association, indicating that RAD21L plays a crucial role in homologue association, whereas REC8 may contribute to this process to a lesser extent [41].

HORMADs exhibit a cohesin-dependent localization to the chromosomal axis and play a crucial role in regulating important events during meiotic prophase. Null mutations of HORMADs in all studied organisms have resulted in SC assembly defects. In mammals, meiotic HORMADs promote AE assembly both dependently and independently of their involvement in double-strand break (DSB) formation [42]. HORMAD1 is loaded to AEs in the absence of SYCP2, but not of meiotic cohesion [43], suggesting that cohesin interacts with HORMAD1 directly. HORMAD2 recruitment is largely dependent on HORMAD1 [44].

SYCP3, the key component of the LEs, plays a crucial role in chromosome compaction by stabilizing chromatin loops [45]. SYCP3 colocalizes with SYCP2 along the chromosome axes, and their recruitment to the axes is interdependent, requiring prior assembly of cohesins [46].

Stochastic optical reconstruction microscopy (STORM) has recently revealed that SYCP3 and the SYCP2 C-terminus form a compact core, around which cohesin complexes, HORMADs, and the N terminus of SYCP2 are arranged [47]. While no direct interactions between cohesin complexes and any component of the axis have been formally identified in mammals to date, the SYCP2 ortholog Red1 in *Saccharomyces cerevisiae* has been shown to form a stable protein complex with meiotic cohesins, suggesting that cohesin complexes might associate directly or indirectly with SYCP2. Furthermore, SYCP2 may stabilize the association of SYCP3 with AEs, as the SYCP3 signal decreases in its absence [48].

At the zygotene stage, the homologue chromosomes align even more closely through the assembly of the TFs and CE acting as a zipper, a process referred to as synapsis. SYCP1 forms homodimers that assemble into the axes, binding to the inner edge of the LEs through its C-terminal region. In the central region, SYCP1 establishes head-to-head interactions through the N-terminal region with SYCP1 dimers originating from the opposite LE, creating a zipper-like structure between the homologues [49]. Both SYCP3 and SYCP1 serve as integral components of the SC, providing the structural framework necessary for the assembly of other complex proteins [50].

The five central element (CE) proteins play critical roles in supporting the SYCP1 lattice, allowing for its continuous and cooperative extension along the entire length of the chromosome. Among the CE proteins, SYCE3, SYCE1, and SIX6OS1 are categorized as synaptic initiation factors, while SYCE2 and TEX12 function as elongation factors. Genetic analysis has demonstrated the cooperative nature of these CE proteins, as deficiencies in any of these proteins result in the loss of synapsis and prevent the loading of SYCP1 [21,22,23,51,52].

Finally, at the pachytene stage, the SC is completely assembled, forming a fully synapsed structure along the chromosome.

Mutant male mice deficient in the genes Sycp1, Sycp3, Sycp2, Rad21l, Rec8, Smc1β, Hormad1, and Hormad2 display a range of abnormal meiotic phenotypes such as synaptic and DSB repair defects, increased apoptosis, and meiotic arrest. Similarly, mutant female mice deficient in these genes exhibit effects ranging from altered chromosome pairing and synapsis, resulting in infertility or subfertility, to normal prophase I progression with no meiotic arrest (see Table S1 for references).

## 4. The Role of the SC in Sustaining Meiotic Recombination

The SC is critical for the maintenance of meiotic recombination, which is essential for the proper segregation of chromosomes during meiosis. Meiotic recombination occurs through the exchange of genetic material between homologous chromosomes. The SC facilitates this exchange by stabilizing the alignment of homologous chromosomes and promoting the formation of COs, which are crucial for the accurate segregation of chromosomes during the first meiotic division [53].

DSB formation and repair, exchange between homologues, and CO formation occur within the context of the chromosome axis. The proper formation of AEs is essential for the initiation of meiotic recombination through the introduction of programmed DSBs catalyzed by the topoisomerase-type enzyme SPO11 and the topoisomerase VIB-like TOP6BL [27,28]. These DSBs are formed at specific sites known as hotspots, which in mice and humans, are determined by the histone methyltransferase PRDM9 [54,55]. The MEI1, MEI4, REC114, and IHO1 complex is responsible for recruiting SPO11 to the chromosomal axis. In turn, the proteins associated with the axis, such as cohesins, HORMADs, SYCP2, and SYCP3, provide the basis for loading this complex. In fact, this complex, as well as other proteins involved in the initial events of DSB repair, such as RPA and the DMC1 and RAD51 recombinases, exhibits axial localization [53,56,57,58]. 

After DSB formation, 5′ overhangs are resected by the exonuclease EXO1, and the RPA complex is recruited to the 3′ ends to protect ssDNA and avoid secondary structure [59]. MEIOB and SPATA22 bind to ssDNA to recruit the BRCA2–HSF2BP–BRME1 complex. Thereafter, RPA is replaced in the ssDNA by RAD51 and DMC1, which are directly recruited by the BRCA2–HSF2BP–BRME1 complex, generating the early recombination nodules (RNs) [60]. SPIDR (scaffold protein involved in DNA repair) regulates the assembly or stability of RAD51/DMC1 on ssDNA [61]. As prophase I proceeds, the early RNs mature into intermediate nodules, and RPA, SPATA22, and MEIOB are loaded again into the ssDNA. Homology seeking and strand invasion mediated by the ATP-dependent recombinases RAD51 and DMC1 facilitate homologs to pair. DMC1 is a meiosis-specific recombinase that needs two auxiliary factor complexes to function, SWI5–SFR1 and HOP2–MND1 [62]. All these interactions are further stabilized by the loading of the SC proteins during synapsis, at zygotene. As the homologues synapse, RAD51 and DMC1 are replaced by the components of the intermediate RNs, which are responsible for the processing of recombination intermediates towards the COs or non-crossovers (NCOs) pathway [63]. 

In mammals, most of the recombination intermediates are resolved as NCOs and only 10% of them are resolved as COs [64,65]. In addition to the aforementioned RPA, SPATA22, and MEIOB, there are a large number of proteins involved in this processing for the final fate of DSBs. These include BLM, TEX11, RNF212, HEI10, HFM1, and the MSH4/MSH5 complex (MutSγ complex), which show partial colocalization with RAD51/DMC1 on synapsed axes [66,67]. The SC, through its CR, is essential for the appropriate processing of intermediate recombination nodules required for maturation of the DSBs into COs [23,68]. The final resolution of the recombination intermediates should occur at pachytene, and because COs are very few, the SC is essential for holding homologs together until the onset of diplotene. This process is mainly mediated by the mismatch repair proteins MLH1/MLH3 (MutLγ complex) required to resolve double Holliday junction recombination intermediates and the exonuclease EXO1 [69,70]. 

In many organisms, most COs are regulated by a phenomenon known as interference, a process by which the presence of a CO on a chromosome decreases the chances of a second CO occurring nearby on the same chromosome. LE and CR components are involved in the regulation of CO interference in different organisms [10]. The presence of at least one CO per bivalent is required for tension at metaphase I and thus correct segregation during anaphase I. Recent evidence suggests that SCs contain mobile subunits and that their assembly is promoted by weak hydrophobic interactions, indicating that it is a phase-separated compartment with liquid crystalline properties. These dynamics properties of the SC can be involved in CO formation and CO interference [71].

Meiotic recombination mutants, such as Spo11^−/−^, are deficient in the formation of DSBs, repair of damaged DNA, or promotion of COs between homologous chromosomes [72]. As a result, these mutants experience a failure in the synapsis of homologous chromosomes, leading to an arrest in meiotic prophase I [73]. On the other hand, synapsis-deficient mutants, such as those lacking the SC, experience defects in the proper alignment and pairing of homologous chromosomes, leading to a failure in meiotic recombination and an arrest in meiotic prophase I [51]. Although there are differences in the specific mechanisms affected, both types of mutants experience failure in meiotic progression and proper gamete production, resulting in infertility in both males and females.

## 5. Infertility

The SC undergoes changes in dynamic state throughout meiosis I that are linked to the progression of other meiotic events. Given the conserved roles of the SC in chromosome pairing, meiotic recombination and COs/chiasmata formation in model organisms, and the resulting phenotype of their loss-of-function mutants, it is expected that mutations in SC coding genes and in genes that encode the associated meiotic recombination machinery might be associated with human fertility.

Since the first discovery in 2014 of a homozygous 1 bp deletion in a cohesin gene (*STAG3*) as a cause of primary ovarian failure (POI) in a consanguineous family [74], several more homozygous or compound heterozygous loss-of-function mutations have been identified in this gene in different POI-affected families [47,48,49,50]. Given the relevance of meiotic cohesins in SC assembly during prophase I and the shared steps of meiosis I in both oogenesis and spermatogenesis, it is not surprising that *STAG3* variants have also been identified in men affected by nonobstructive azoospermia (NOA) [75,76,77]. In fact, very recently, an in-frame STAG3 variant associated with both NOA and POI was reported for the first time, as we had previously predicted [28,78]. Furthermore, recent studies have identified genetic variants of the other meiotic-specific cohesins SMC1β, REC8, and RAD21L associated with infertility phenotypes including POI and NOA [76,79,80,81,82]. Moreover, the recent discovery of cohesin involvement in RNA splicing can provide a potential avenue for investigating the etiology of idiopathic infertility, in which non-null hypomorphic mutations in meiotic cohesins may be disrupting the transcriptional program of gametogenesis [83].

A biallelic loss-of-function variant in *HORMAD1*, which also localizes to AE and is required for proper SC assembly, has very recently been identified in three NOA-affected brothers within a large consanguineous family, revealing that this SC component is an essential genetic factor in the meiotic process in humans [84]. 

The ATPase TRIP13 acts as a negative regulator of the HORMA proteins, MAD2 in mitosis, and HORMAD1/2 in meiosis, and plays a critical role in chromosome synapsis by removing HORMAD2 from synapsed chromosomal axes. While recurrent mutations in *TRIP13* have been reported to cause Wilms’ tumors, pathogenic variants that cause female infertility due to oocyte maturation arrest have recently been identified in three families without any other abnormalities [85].

Regarding the structural subunits of the SC, infertility-related mutations have only been identified in four genes in humans, which include two LE components (SYCP2 and SYCP3) and two CE components (SYCE1 and SIX6OS1) [15,86]. However, given the infertility phenotype observed in null mutant mice for all SC structural components, it is expected that infertility-related mutations in the other SC genes will be identified as more cases are studied. Interestingly, most of the identified mutations in CE genes are homozygous, while mutations in LE genes have a dominant-negative effect [15]. One possible explanation is that mutant LE proteins can incorporate into AEs and interfere with the correct formation of the SC, whereas mutations in CE proteins interrupt their union with other SC proteins. Clinical mutations in *SYCE1* associated with NOA and POI have been reported to affect their interaction with SIX6OS1 and, consequently, the structural assembly of the SC and its role in meiosis [87].

Proteasomal degradation of proteins is an essential mechanism for many developmental programs, including gametogenesis. The spermatoproteasome is a type of proteasome found exclusively in mammalian germ cells and is essential for spermatogenesis [88]. The spermatoproteasome is characterized by the presence of the testis-specific proteasomal subunit PSMA8 that localizes to and is dependent on the CR of the SC. The spermatoproteasome is responsible for the proteostasis of several key meiotic players, including SYCP3, SYCP1, and TRIP13. PSMA8 is among the top seven candidate genes found in an unbiased differential proteomic profiling of spermatozoa proteins from infertile men with a varicocele, suggesting a causal role in the severity of the disease [23,89].

As stated previously, meiotic recombination events, from DSB induction to CO formation, take place in the context of the SC and must be tightly controlled by different mechanisms and pathways including the SC itself [53]. Non-formation or insufficient formation of DSBs can cause homologous mismatching and chromosome missegregation. On the other hand, an increase in the number of DSBs would increase the risk of mutations and genomic instability. In addition, the DSBs/COs must be generated in the correct places to protect gene-promoter regions and other functional genomic elements [54,90,91].

Mutations in many genes related to DSB formation that cause human infertility have been identified. Interestingly, variants in genes responsible for DSB formation such as *SPO11*, *TOP6BL*, *MEI1*, and *REC114* have been proven causative for POI and/or NOA [92,93,94,95,96]. More recently, a new REC114 interactor has been identified, ANKRD31, that regulates the distribution of DSB and is essential for the recombination between X and Y chromosomes [97]. Pathogenic heterozygous variants in *ANKRD31*, which disturbed its interaction with REC114, have been recently identified in POI-affected women. In addition, this study also describes the presence in POI patients of pathogenic heterozygous variants in PRMD9 that impaired its methyltransferase activity [98]. These results suggest that the impact of these two genes in POI development is dosage-dependent.

Gene variants involved in the repair of the programmed DSBs within meiotic-specific components of the RNs such as MEIOB, SPATA22, TEX11, HSF2BP, DMC1, RNF212, HFM1, or the DNA mismatch repair proteins of the MutSγ complex, MSH4 and MSH5, have been associated with infertility in both sexes (POI, NOA) [60,75,99,100,101,102,103,104]. In addition, a homozygous 3 bp deletion in the *HOP2* gene has been identified as the genetic cause of 46,XX female gonadal dysgenesis (46XX-GD), a rare disorder of sex development characterized by a primary ovarian defect [105].

The HR machinery is fundamental for the repair of DSBs during mammalian gametogenesis, as well as the repair of DSBs caused by both exogenous and endogenous factors in somatic cells. Therefore, mutations in genes with dual roles in DNA mismatch repair and meiosis, such as *SPIDR*, *SWI5*, and *MLH3*, have been reported as causative for human infertility [106,107,108]. These findings demonstrate that although these genes are not specific to meiosis, they are essentials for its proper progression. 

This is also the case for the MCM8-MCM9 and HELQ helicases that are required for HR repair induced by DNA interstrand crosslinks (ICLs) [109,110] and for late stage D-loop dissolution in meiosis [111]. HROB recruits MCM8/9 helicases to sites of DNA damage, and this pathway redundantly acts with the HELQ helicase [112]. Recently, it has also been described that RAD51B interacts with HELQ and RAD51 and works together with them to promote/mediate meiotic recombination [113]. Mutations in these five genes have also been associated with nonsyndromic POI [82,107,111,113]. In this sense, the Fanconi anemia (FA) pathway is well known for its essential role in ICL repair. Interestingly, both male and female FA patients show severely reduced fertility. In female FA patients, the reduced fertility manifests as POI, while in males, it manifests as NOA and Sertoli cell-only syndrome (SCOS) [114]. Moreover, recent reports describe patients with mutations in *FANCA*, *FANCM*, or *XRCC2*/*FANCU* who were diagnosed with FA after they displayed NOA and SCOS [115]. It is also known that defects in BRCA2 (FANCD1) can lead to cancer predisposition and FA. However, a very recent study described a homozygous hypomorphic BRCA2 variant in a patient with POI but no other pathology [116]. In fact, most loss-of-function mouse models of these proteins are not lethal but are infertile [61,112,117,118,119].

Very recent studies have identified variants in the human mismatch repair proteins MLH1/3 causing gamete aneuploidy, pregnancy loss, and premature reproductive aging [120]. According to that, a variant in the *SIX6OS1* gene was also identified as an influencing polymorphism affecting the human recombination rate and has been associated with age at menarche, an indirect fertility trait [23]. These results suggest that hypomorphic variants of genes affecting the recombination rate may predispose women to pregnancy loss due to an increased incidence of aneuploidy oocytes.

Considering that the majority of genetic variants responsible for human infertility exhibit a recessive pattern, the assessment of mouse gene knockouts serves as a valuable resource to evaluate the clinical significance of novel gene candidates. Therefore, in Table S1, we have included relevant information regarding the phenotype of these mouse gene knockouts, along with additional details concerning the list of genes reviewed in this study.

## 6. Cancer

The process of meiotic recombination is essential for fertility and integrity of the genome. Recent studies have revealed that meiotic genes that are normally restricted to germ cells can become aberrantly expressed in human cancers [121]. Due to the role of these genes in DSB repair by the exchange of genetic information between homologous chromosomes, their aberrant expression in tumor cells drives genetic instability that can have catastrophic oncogenic consequences [122]. In fact, several meiotic genes have been included within the category of cancer/testis (CT) genes. The expression of CT genes is normally restricted to the germ cells of the testis, where they are believed to play a role in spermatogenesis [123]. However, the expression of many CT genes is upregulated in different cancers due to epigenetic mechanisms, such as DNA methylation and histone modifications, which can be altered in cancer cells, leading to their aberrant expression. CT genes are thought to play a critical role in the initiation and progression of cancer by promoting cell proliferation, migration, and invasion [124]. Considering the abnormal re-expression of these ‘meiotic’ genes, we have conducted individual analyses of each gene listed in this study, describing their RNA transcription in somatic tissues, cell lines, and cancer using publicly available databases (Table S1 and Figure S1). It is important to note that the precise mechanism through which CT genes contribute to tumorigenesis is not yet fully understood and has not been formally proven.

Moreover, it is worth noting that several structural proteins of the SC, such as SYCP1/3, SYCE1-2, and TEX12, have been observed to be ectopically expressed in different types of human cancers [125,126,127,128,129] (Table S1 and Figure S1). Given the function of these proteins in chromosome structure and DNA recombination, their expression outside of the meiotic context could be detrimental to chromosomal stability. For example, ectopic expression of SYCP3 in somatic cells forms a complex with BRCA2 that impairs the recruitment of RAD51 to resected DSBs and reduces HR efficiency, which potentially may result in oncogenic genome instability [130]. Additionally, HORMAD1 expression has been associated with drug resistance in various types of cancer by promoting efficient resection of DSBs, making repair of damage produced in tumor cells more effective [131,132,133,134].

The expression of the meiotic cohesin subunits as CT genes is also relevant because their function in chromosome pairing, sister chromatid cohesion, and DNA repair differ from the roles of somatic cohesins. In this regard, REC8, SMC1β, and STAG3 have been found to be widely expressed in human cancers, whereas the expression of the RAD21L subunit is practically undetectable [135]. Recent studies link aberrant expression of meiotic cohesins with aneuploidy, chromosomal mutations, and altered gene expression in affected cells. In addition, they show that the expression of REC8 in human cancer cell lines triggers a mild mitotic phenotype, so its presence is permissible in tumors, while the expression of RAD21L induces a cell cycle arrest that leads to chromosomal instability, which would explain its underrepresentation in tumors, but would not rule out its transient activation in early oncogenic events [136] (see also Table S1 and Figure S1). 

Interestingly, aberrant expression of PRMD9 in human tumors is associated with structural variant breakpoints frequently neighboring the DNA motif recognized by PRDM9, suggesting a potential role for this specific meiotic gene in the genomic instability observed in cancer cells [137]. In relation to the endonuclease SPO11, to date, no direct evidence has been found indicating that SPO11 has an oncogenic function in humans [122].

Expression of various meiotic recombination genes such as *DMC1*, *HOP2-MND1*, *MEIOB*, *SPATA22*, *HSF2BP*, and *MSH4* in somatic cell tumors has also been described [138]. Aberrant expression of these genes could interact with somatic HR proteins and interfere in the correct progression of the DNA repair process (Table S1 and Figure S1). The first strong evidence linking meiotic recombination gene expression and carcinogenesis came from the finding that the HOP2–MND1 meiotic complex, through interaction with RAD51, is required for the homologous recombination events that maintain chromosome ends in cancer cells relying on alternative lengthening of telomeres (ALT) [139]. Interestingly, expression of DMC1 in cancer cells provides an additional repair mechanism to evade cell death caused by DNA damage and is associated with depolyploidization events [140,141]. These results suggest that activation of meiotic genes might allow a change from pro-mitotic to a pro-meiotic division regimen to reduce the chromosome number and facilitate recombination that decreases the mutation load of aneuploidy and lethality in the chemoresistant tumor cells.

As mentioned above, many genes involved in HR during gametogenesis are also essential for the repair of DSBs in somatic cells. HR deficiency leads to genetic instability, and as expected, alteration in HR genes is prevalent among many cancer types. It is well known that BRCA2 is one of the most mutated HR genes in familial breast/ovarian cancers [142]. Moreover, germline mutations in several HR genes including *RAD51*, *XRCC2*, *FANCA*, *FANCM,* and the previously mentioned *BRCA2* are responsible for subgroups of FA, a phenotypically heterogeneous recessive disorder with predisposition to hematologic and solid tumors [143]. Mutation in the *RAD51* paralog, *RAD51B*, has been also associated with male and female breast cancer, ovary cancer, prostate cancer, and pituitary adenoma [113].

As already mentioned, MLH1 and MLH3 are part of the MMR, a pathway responsible for maintaining genome stability. If the MMR does not function normally in somatic cells, the overall mutation rate increases, and microsatellite instability (MSI) occurs, triggering the tumor phenotype. Mutations in *MLH1* and *MLH3* have been associated with different types of cancer including colon, rectum, endometrium, hereditary nonpolyposis colorectal cancer type 7 (HNPCC7), and low-grade glioma [144]. In addition, inactivating mutations in *MLH1* are the main cause of Lynch syndrome, characterized by autosomal dominant inheritance of early-onset colorectal cancer (CRC) and associated with increased risk of other cancers, and its promoter region is often epigenetically silenced in a variety of sporadic cancers [145] (see also Table S1 and Figure S1).

## 7. Conclusions

The SC is a highly conserved meiosis-specific megaprotein structure found in most sexually reproducing organisms. It plays a crucial role in the prophase of meiosis I by forming between homologous chromosomes. The mammalian SC serves as a scaffold that facilitates and maintains synapsis, the pairing of homologous chromosomes, along their entire length. It enables the exchange of genetic material between homologs, promoting genetic diversity. Additionally, in most species, the SC supports the formation of crossovers (COs), which are essential for the accurate segregation of chromosomes during the first meiotic division. In summary, the SC is a key player in meiosis, ensuring proper chromosome dynamics and genetic recombination, ultimately contributing to successful sexual reproduction.

In humans, errors in chromosome segregation during meiosis are a major cause of miscarriages, infertility, and birth defects. The precise mechanisms underlying these errors are still not fully understood, and the study of the SC and its associated proteins is therefore of great importance in understanding the fundamental processes underlying meiotic recombination and their role in human disease.

The functional analysis of most of these proteins relies on genetic investigations conducted in model organisms such as yeast, worms, and mice, due to their significant conservation of functional attributes. Furthermore, the mechanisms underlying the involvement of certain meiosis-specific proteins have been inferred based on their well-established roles as related homologs in somatic DNA repair.

Although meiotic recombination and synapsis are distinct processes, mutations in genes involved in either process can result in similar meiotic progression defects, ultimately leading to infertility and other developmental abnormalities (see Table S1). In humans, mutations in genes encoding the SC and associated meiotic recombination machinery have been linked to fertility issues, while aberrant expression of meiotic genes in somatic cells is observed in certain cancers, causing genetic instability and potentially catastrophic oncogenic consequences. Further investigation into the SC and its associated proteins in diverse model organisms will enhance our understanding of how meiotic complexes and proteins affect human reproductive health, as well as provide insights into their mechanistic role in tumor development.

## Data Availability

Not applicable.

## Supplementary Materials

The following supporting information can be downloaded at https://www.mdpi.com/article/10.3390/cells12131718/s1, Figure S1: Expression data of genes mentioned in the review; Table S1: Additional information of genes mentioned in the review.
