# Supplementary material for: Synaptonemal Complex in Human Biology and Disease
Source: Cells. 2023 Jun 25;12(13):1718. doi: 10.3390/cells12131718 (PMC10341275; doi:10.3390/cells12131718)
Supplement: Supplementary file 1 [file cells-12-01718-s001.zip › cells-2439912-supplementary.pdf]

Figure S1. Expression data of genes mentioned in the review

The RNA expression of the genes of interest was also analyzed using data from the TCGA Pan Cancer Atlas studies, extracted from the cBioPortal (https://www.cbioportal.org/). The order of the genes in the analysis corresponds to the order presented in Table 1. Additional analyses, such as mutation frequency, copy number variation (CNV), methylation status, and more, can be conducted at the cBioPortal by modifying the query using the uploaded list of genes of interest available at this link:

https://www.cbioportal.org/results/plots?tab_index=tab_visualize&Action=Submit&session_id=646c6bb8e4f00e3f62d80fbe&plots_horz_selection=%7B%22dataType%22%3A%22clinical_attribute%22%2C%22selectedDataSourceOption%22%3A%22CANCER_STUDY%22%7D&plots_vert_selection=%7B%22selectedGeneOption%22%3A642636%2C%22dataType%22%3A%22MRNA_EXPRESSION%22%2C%22selectedDataSourceOption%22%3A%22rna_seq_v2_mrna%22%2C%22logScale%22%3A%22true%22%7D&plots_coloring_selection=%7B%22colorByCopyNumber%22%3A%22false%22%7D

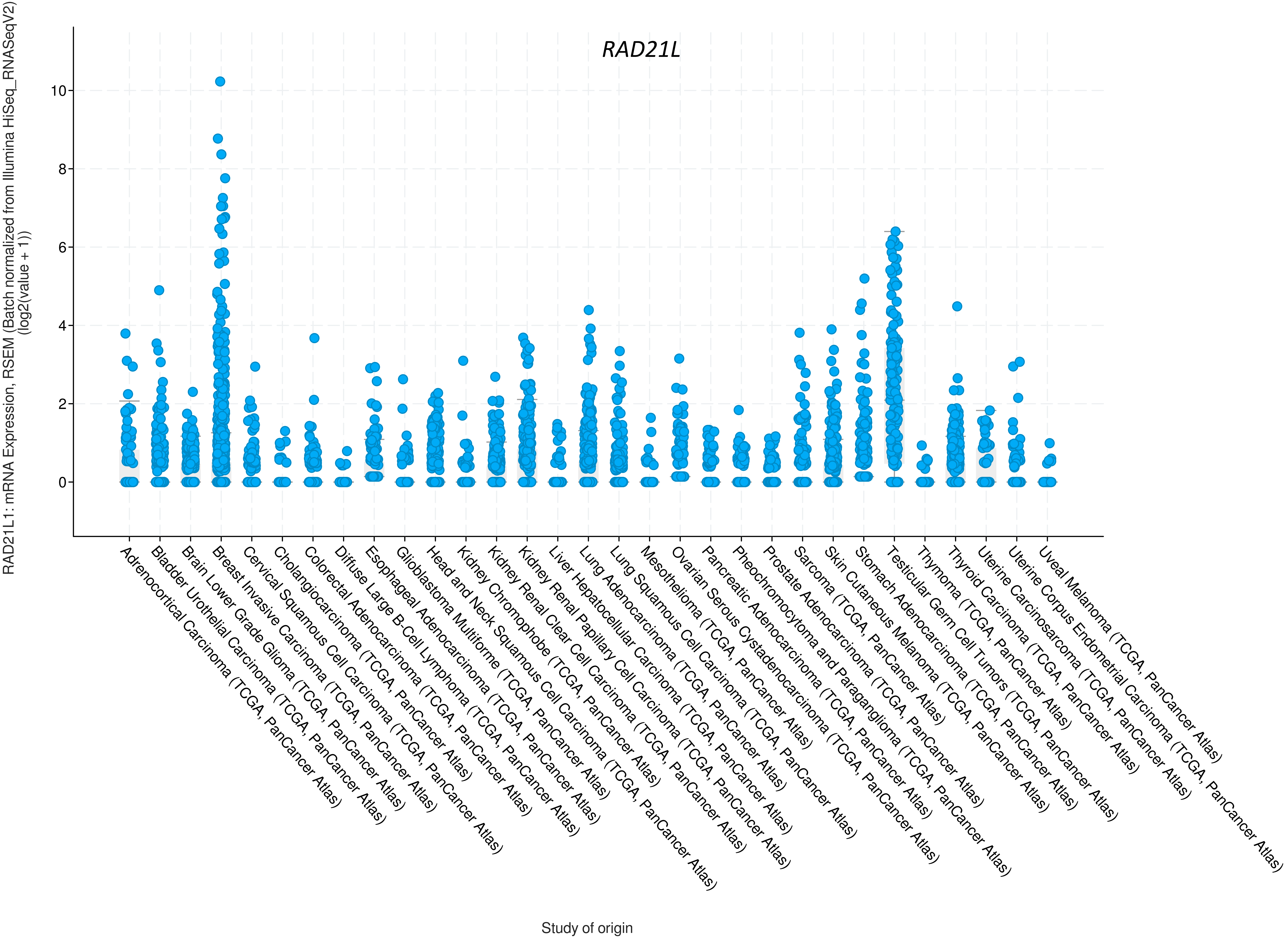

RAD21L
RAD21L1: mRNA Expression, RSEM (Batch normalized from Illumina HiSeq_RNASeqV2) (log2(value + 1))
10
8
6
4
2
0
Study of origin
Adrenocortical Carcinoma (TCGA, PanCancer Atlas)
Bladder Urothelial Carcinoma (TCGA, PanCancer Atlas)
Brain Lower Grade Glioma (TCGA, PanCancer Atlas)
Breast Invasive Carcinoma (TCGA, PanCancer Atlas)
Cervical Squamous Cell Carcinoma (TCGA, PanCancer Atlas)
Cholangiocarcinoma (TCGA, PanCancer Atlas)
Colorectal Adenocarcinoma (TCGA, PanCancer Atlas)
Diffuse Large B-Cell Lymphoma (TCGA, PanCancer Atlas)
Esophageal Adenocarcinoma (TCGA, PanCancer Atlas)
Glioblastoma Multiforme (TCGA, PanCancer Atlas)
Head and Neck Squamous Cell Carcinoma (TCGA, PanCancer Atlas)
Kidney Chromophobe (TCGA, PanCancer Atlas)
Kidney Renal Clear Cell Carcinoma (TCGA, PanCancer Atlas)
Kidney Renal Papillary Cell Carcinoma (TCGA, PanCancer Atlas)
Liver Hepatocellular Carcinoma (TCGA, PanCancer Atlas)
Lung Adenocarcinoma (TCGA, PanCancer Atlas)
Lung Squamous Cell Carcinoma (TCGA, PanCancer Atlas)
Mesothelioma (TCGA, PanCancer Atlas)
Ovarian Serous Cystadenocarcinoma (TCGA, PanCancer Atlas)
Pancreatic Adenocarcinoma (TCGA, PanCancer Atlas)
Pheochromocytoma and Paraganglioma (TCGA, PanCancer Atlas)
Prostate Adenocarcinoma (TCGA, PanCancer Atlas)
Sarcoma (TCGA, PanCancer Atlas)
Skin Cutaneous Melanoma (TCGA, PanCancer Atlas)
Stomach Adenocarcinoma (TCGA, PanCancer Atlas)
Testicular Germ Cell Tumors (TCGA, PanCancer Atlas)
Thymoma (TCGA, PanCancer Atlas)
Thyroid Carcinoma (TCGA, PanCancer Atlas)
Uterine Carcinosarcoma (TCGA, PanCancer Atlas)
Uterine Corpus Endometrial Carcinoma (TCGA, PanCancer Atlas)
Uveal Melanoma (TCGA, PanCancer Atlas)

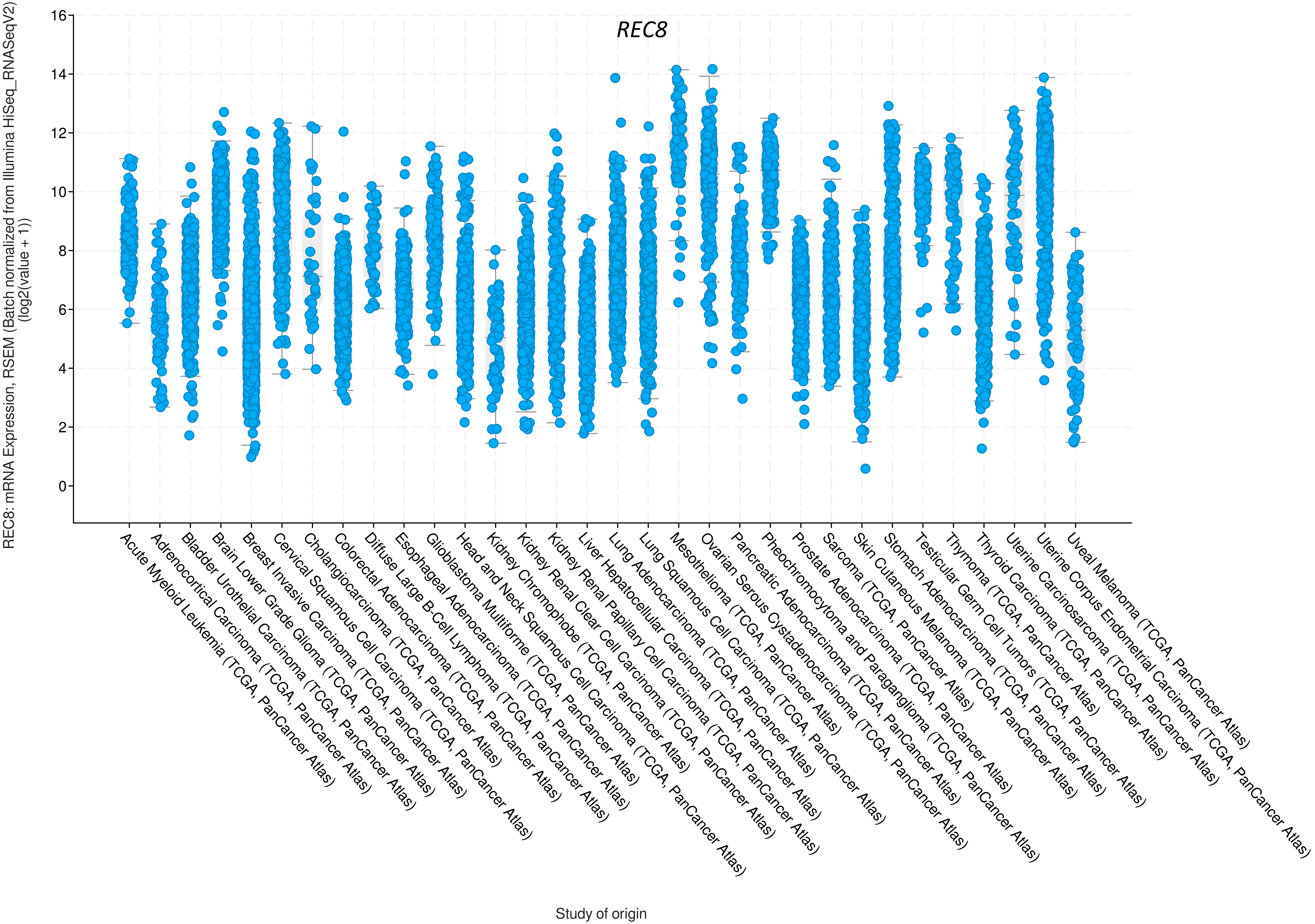
REC8
REC8: mRNA Expression, RSEM (Batch normalized from Illumina HiSeq_RNASeqV2) (log2(value + 1))
16
14
12
10
8
6
4
2
0
Study of origin
Acute Myeloid Leukemia (TCGA, PanCancer Atlas)
Adrenocortical Carcinoma (TCGA, PanCancer Atlas)
Bladder Urothelial Carcinoma (TCGA, PanCancer Atlas)
Brain Lower Grade Glioma (TCGA, PanCancer Atlas)
Breast Invasive Carcinoma (TCGA, PanCancer Atlas)
Cervical Squamous Cell Carcinoma (TCGA, PanCancer Atlas)
Cholangiocarcinoma (TCGA, PanCancer Atlas)
Colorectal Adenocarcinoma (TCGA, PanCancer Atlas)
Diffuse Large B-Cell Lymphoma (TCGA, PanCancer Atlas)
Esophageal Adenocarcinoma (TCGA, PanCancer Atlas)
Glioblastoma Multiforme (TCGA, PanCancer Atlas)
Head and Neck Squamous Cell Carcinoma (TCGA, PanCancer Atlas)
Kidney Chromophobe (TCGA, PanCancer Atlas)
Kidney Renal Clear Cell Carcinoma (TCGA, PanCancer Atlas)
Kidney Renal Papillary Cell Carcinoma (TCGA, PanCancer Atlas)
Liver Hepatocellular Carcinoma (TCGA, PanCancer Atlas)
Lung Adenocarcinoma (TCGA, PanCancer Atlas)
Lung Squamous Cell Carcinoma (TCGA, PanCancer Atlas)
Mesothelioma (TCGA, PanCancer Atlas)
Ovarian Serous Cystadenocarcinoma (TCGA, PanCancer Atlas)
Pancreatic Adenocarcinoma (TCGA, PanCancer Atlas)
Pheochromocytoma and Paraganglioma (TCGA, PanCancer Atlas)
Prostate Adenocarcinoma (TCGA, PanCancer Atlas)
Sarcoma (TCGA, PanCancer Atlas)
Skin Cutaneous Melanoma (TCGA, PanCancer Atlas)
Stomach Adenocarcinoma (TCGA, PanCancer Atlas)
Testicular Germ Cell Tumors (TCGA, PanCancer Atlas)
Thymoma (TCGA, PanCancer Atlas)
Thyroid Carcinoma (TCGA, PanCancer Atlas)
Uterine Carcinosarcoma (TCGA, PanCancer Atlas)
Uterine Corpus Endometrial Carcinoma (TCGA, PanCancer Atlas)
Uveal Melanoma (TCGA, PanCancer Atlas)

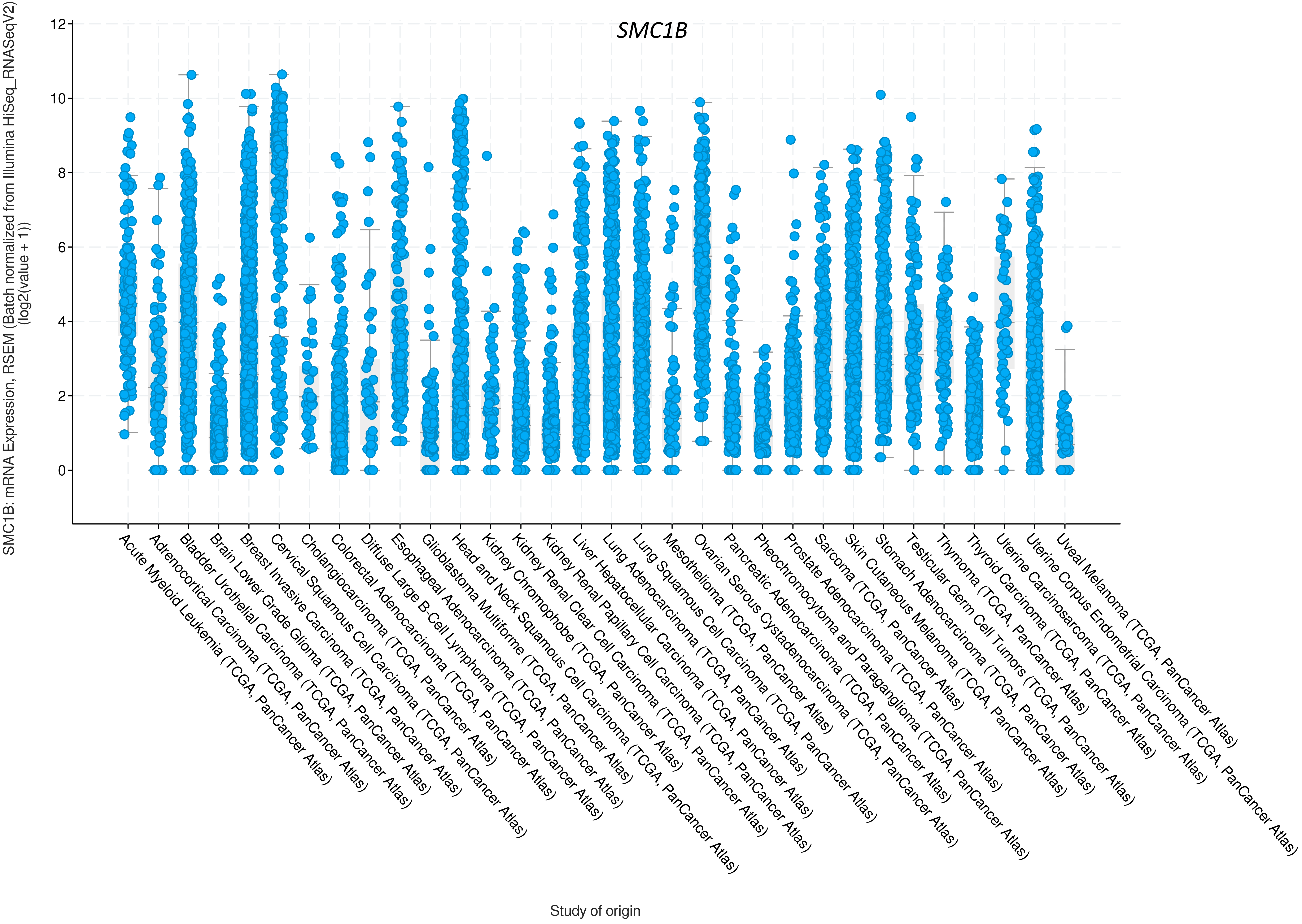

SMC1B
SMC1B: mRNA Expression, RSEM (Batch normalized from Illumina HiSeq_RNASeqV2) (log2(value + 1))
12
10
8
6
4
2
0
Acute Myeloid Leukemia (TCGA, PanCancer Atlas)
Adrenocortical Carcinoma (TCGA, PanCancer Atlas)
Bladder Urothelial Carcinoma (TCGA, PanCancer Atlas)
Brain Lower Grade Glioma (TCGA, PanCancer Atlas)
Breast Invasive Carcinoma (TCGA, PanCancer Atlas)
Cervical Squamous Cell Carcinoma (TCGA, PanCancer Atlas)
Cholangiocarcinoma (TCGA, PanCancer Atlas)
Colorectal Adenocarcinoma (TCGA, PanCancer Atlas)
Diffuse Large B-Cell Lymphoma (TCGA, PanCancer Atlas)
Esophageal Adenocarcinoma (TCGA, PanCancer Atlas)
Glioblastoma Multiforme (TCGA, PanCancer Atlas)
Head and Neck Squamous Cell Carcinoma (TCGA, PanCancer Atlas)
Kidney Chromophobe (TCGA, PanCancer Atlas)
Kidney Renal Clear Cell Carcinoma (TCGA, PanCancer Atlas)
Kidney Renal Papillary Cell Carcinoma (TCGA, PanCancer Atlas)
Liver Hepatocellular Carcinoma (TCGA, PanCancer Atlas)
Lung Adenocarcinoma (TCGA, PanCancer Atlas)
Lung Squamous Cell Carcinoma (TCGA, PanCancer Atlas)
Mesothelioma (TCGA, PanCancer Atlas)
Ovarian Serous Cystadenocarcinoma (TCGA, PanCancer Atlas)
Pancreatic Adenocarcinoma (TCGA, PanCancer Atlas)
Pheochromocytoma and Paraganglioma (TCGA, PanCancer Atlas)
Prostate Adenocarcinoma (TCGA, PanCancer Atlas)
Sarcoma (TCGA, PanCancer Atlas)
Skin Cutaneous Melanoma (TCGA, PanCancer Atlas)
Stomach Adenocarcinoma (TCGA, PanCancer Atlas)
Testicular Germ Cell Tumors (TCGA, PanCancer Atlas)
Thymoma (TCGA, PanCancer Atlas)
Thyroid Carcinoma (TCGA, PanCancer Atlas)
Uterine Carcinosarcoma (TCGA, PanCancer Atlas)
Uterine Corpus Endometrial Carcinoma (TCGA, PanCancer Atlas)
Uveal Melanoma (TCGA, PanCancer Atlas)
Study of origin

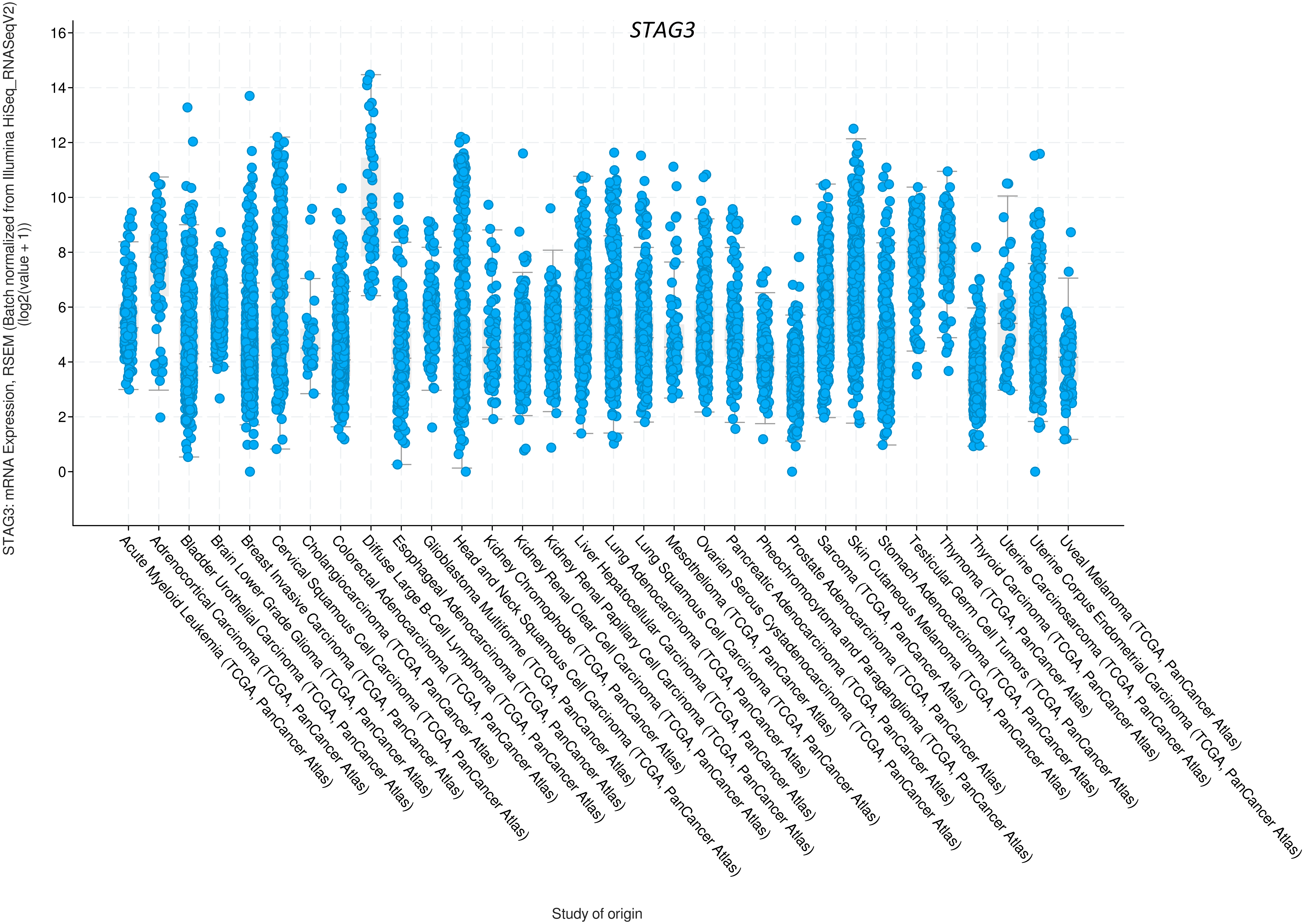
STAG3
STAG3: mRNA Expression, RSEM (Batch normalized from Illumina HiSeq_RNASeqV2) (log2(value + 1))
16
14
12
10
8
6
4
2
0
Acute Myeloid Leukemia (TCGA, PanCancer Atlas)
Adrenocortical Carcinoma (TCGA, PanCancer Atlas)
Bladder Urothelial Carcinoma (TCGA, PanCancer Atlas)
Brain Lower Grade Glioma (TCGA, PanCancer Atlas)
Breast Invasive Carcinoma (TCGA, PanCancer Atlas)
Cervical Squamous Cell Carcinoma (TCGA, PanCancer Atlas)
Cholangiocarcinoma (TCGA, PanCancer Atlas)
Colorectal Adenocarcinoma (TCGA, PanCancer Atlas)
Diffuse Large B-Cell Lymphoma (TCGA, PanCancer Atlas)
Esophageal Adenocarcinoma (TCGA, PanCancer Atlas)
Glioblastoma Multiforme (TCGA, PanCancer Atlas)
Head and Neck Squamous Cell Carcinoma (TCGA, PanCancer Atlas)
Kidney Chromophobe (TCGA, PanCancer Atlas)
Kidney Renal Clear Cell Carcinoma (TCGA, PanCancer Atlas)
Kidney Renal Papillary Cell Carcinoma (TCGA, PanCancer Atlas)
Liver Hepatocellular Carcinoma (TCGA, PanCancer Atlas)
Lung Adenocarcinoma (TCGA, PanCancer Atlas)
Lung Squamous Cell Carcinoma (TCGA, PanCancer Atlas)
Mesothelioma (TCGA, PanCancer Atlas)
Ovarian Serous Cystadenocarcinoma (TCGA, PanCancer Atlas)
Pancreatic Adenocarcinoma (TCGA, PanCancer Atlas)
Pheochromocytoma and Paraganglioma (TCGA, PanCancer Atlas)
Prostate Adenocarcinoma (TCGA, PanCancer Atlas)
Sarcoma (TCGA, PanCancer Atlas)
Skin Cutaneous Melanoma (TCGA, PanCancer Atlas)
Stomach Adenocarcinoma (TCGA, PanCancer Atlas)
Testicular Germ Cell Tumors (TCGA, PanCancer Atlas)
Thymoma (TCGA, PanCancer Atlas)
Thyroid Carcinoma (TCGA, PanCancer Atlas)
Uterine Carcinosarcoma (TCGA, PanCancer Atlas)
Uterine Corpus Endometrial Carcinoma (TCGA, PanCancer Atlas)
Uveal Melanoma (TCGA, PanCancer Atlas)
Study of origin

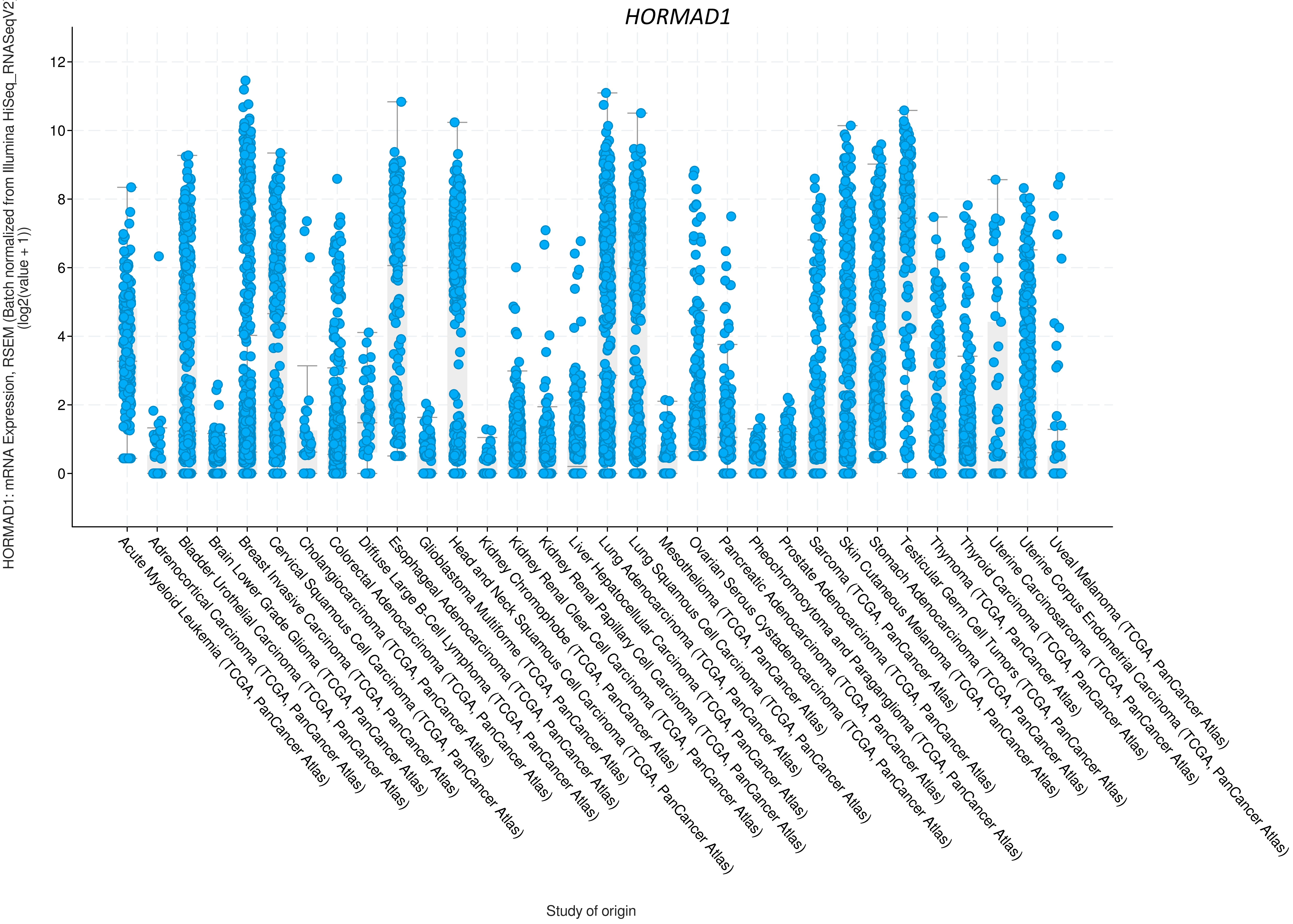
HORMAD1
HORMAD1: mRNA Expression, RSEM (Batch normalized from Illumina HiSeq_RNASeqV2) (log2(value + 1))
Study of origin
0
2
4
6
8
10
12
Acute Myeloid Leukemia (TCGA, PanCancer Atlas)
Adrenocortical Carcinoma (TCGA, PanCancer Atlas)
Bladder Urothelial Carcinoma (TCGA, PanCancer Atlas)
Brain Lower Grade Glioma (TCGA, PanCancer Atlas)
Breast Invasive Carcinoma (TCGA, PanCancer Atlas)
Cervical Squamous Cell Carcinoma (TCGA, PanCancer Atlas)
Cholangiocarcinoma (TCGA, PanCancer Atlas)
Colorectal Adenocarcinoma (TCGA, PanCancer Atlas)
Diffuse Large B-Cell Lymphoma (TCGA, PanCancer Atlas)
Esophageal Adenocarcinoma (TCGA, PanCancer Atlas)
Glioblastoma Multiforme (TCGA, PanCancer Atlas)
Head and Neck Squamous Cell Carcinoma (TCGA, PanCancer Atlas)
Kidney Chromophobe (TCGA, PanCancer Atlas)
Kidney Renal Clear Cell Carcinoma (TCGA, PanCancer Atlas)
Kidney Renal Papillary Cell Carcinoma (TCGA, PanCancer Atlas)
Liver Hepatocellular Carcinoma (TCGA, PanCancer Atlas)
Lung Adenocarcinoma (TCGA, PanCancer Atlas)
Lung Squamous Cell Carcinoma (TCGA, PanCancer Atlas)
Mesothelioma (TCGA, PanCancer Atlas)
Ovarian Serous Cystadenocarcinoma (TCGA, PanCancer Atlas)
Pancreatic Adenocarcinoma (TCGA, PanCancer Atlas)
Pheochromocytoma and Paraganglioma (TCGA, PanCancer Atlas)
Prostate Adenocarcinoma (TCGA, PanCancer Atlas)
Sarcoma (TCGA, PanCancer Atlas)
Skin Cutaneous Melanoma (TCGA, PanCancer Atlas)
Stomach Adenocarcinoma (TCGA, PanCancer Atlas)
Testicular Germ Cell Tumors (TCGA, PanCancer Atlas)
Thymoma (TCGA, PanCancer Atlas)
Thyroid Carcinoma (TCGA, PanCancer Atlas)
Uterine Carcinosarcoma (TCGA, PanCancer Atlas)
Uterine Corpus Endometrial Carcinoma (TCGA, PanCancer Atlas)
Uveal Melanoma (TCGA, PanCancer Atlas)

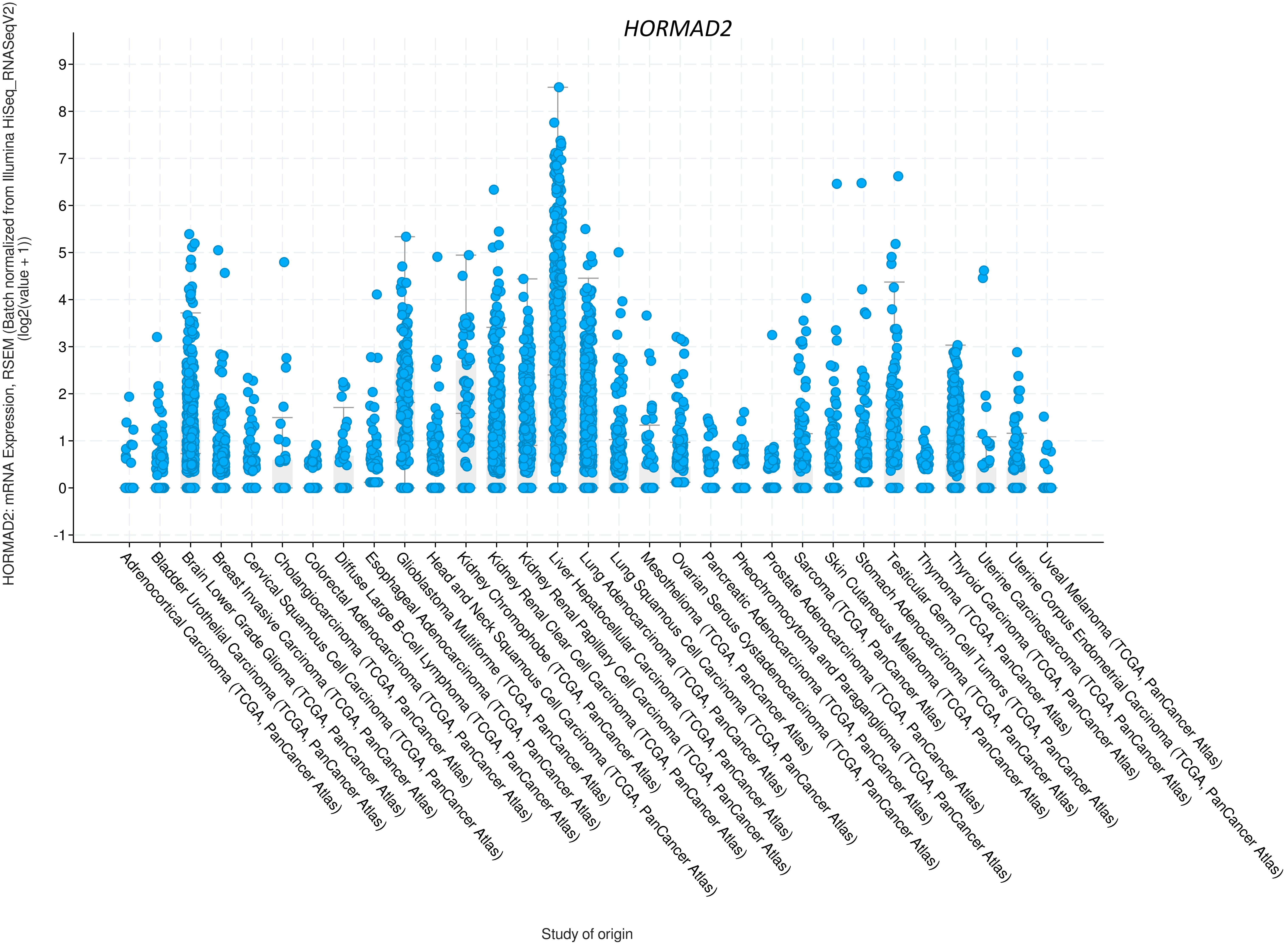

HORMAD2
HORMAD2: mRNA Expression, RSEM (Batch normalized from Illumina HiSeq_RNASeqV2) (log2(value + 1))
Study of origin
9
8
7
6
5
4
3
2
1
0
-1
Adrenocortical Carcinoma (TCGA, PanCancer Atlas)
Bladder Urothelial Carcinoma (TCGA, PanCancer Atlas)
Brain Lower Grade Glioma (TCGA, PanCancer Atlas)
Breast Invasive Carcinoma (TCGA, PanCancer Atlas)
Cervical Squamous Cell Carcinoma (TCGA, PanCancer Atlas)
Cholangiocarcinoma (TCGA, PanCancer Atlas)
Colorectal Adenocarcinoma (TCGA, PanCancer Atlas)
Diffuse Large B-Cell Lymphoma (TCGA, PanCancer Atlas)
Esophageal Adenocarcinoma (TCGA, PanCancer Atlas)
Glioblastoma Multiforme (TCGA, PanCancer Atlas)
Head and Neck Squamous Cell Carcinoma (TCGA, PanCancer Atlas)
Kidney Chromophobe (TCGA, PanCancer Atlas)
Kidney Renal Clear Cell Carcinoma (TCGA, PanCancer Atlas)
Kidney Renal Papillary Cell Carcinoma (TCGA, PanCancer Atlas)
Liver Hepatocellular Carcinoma (TCGA, PanCancer Atlas)
Lung Adenocarcinoma (TCGA, PanCancer Atlas)
Lung Squamous Cell Carcinoma (TCGA, PanCancer Atlas)
Mesothelioma (TCGA, PanCancer Atlas)
Ovarian Serous Cystadenocarcinoma (TCGA, PanCancer Atlas)
Pancreatic Adenocarcinoma (TCGA, PanCancer Atlas)
Pheochromocytoma and Paraganglioma (TCGA, PanCancer Atlas)
Prostate Adenocarcinoma (TCGA, PanCancer Atlas)
Sarcoma (TCGA, PanCancer Atlas)
Skin Cutaneous Melanoma (TCGA, PanCancer Atlas)
Stomach Adenocarcinoma (TCGA, PanCancer Atlas)
Testicular Germ Cell Tumors (TCGA, PanCancer Atlas)
Thymoma (TCGA, PanCancer Atlas)
Thyroid Carcinoma (TCGA, PanCancer Atlas)
Uterine Carcinosarcoma (TCGA, PanCancer Atlas)
Uterine Corpus Endometrial Carcinoma (TCGA, PanCancer Atlas)
Uveal Melanoma (TCGA, PanCancer Atlas)

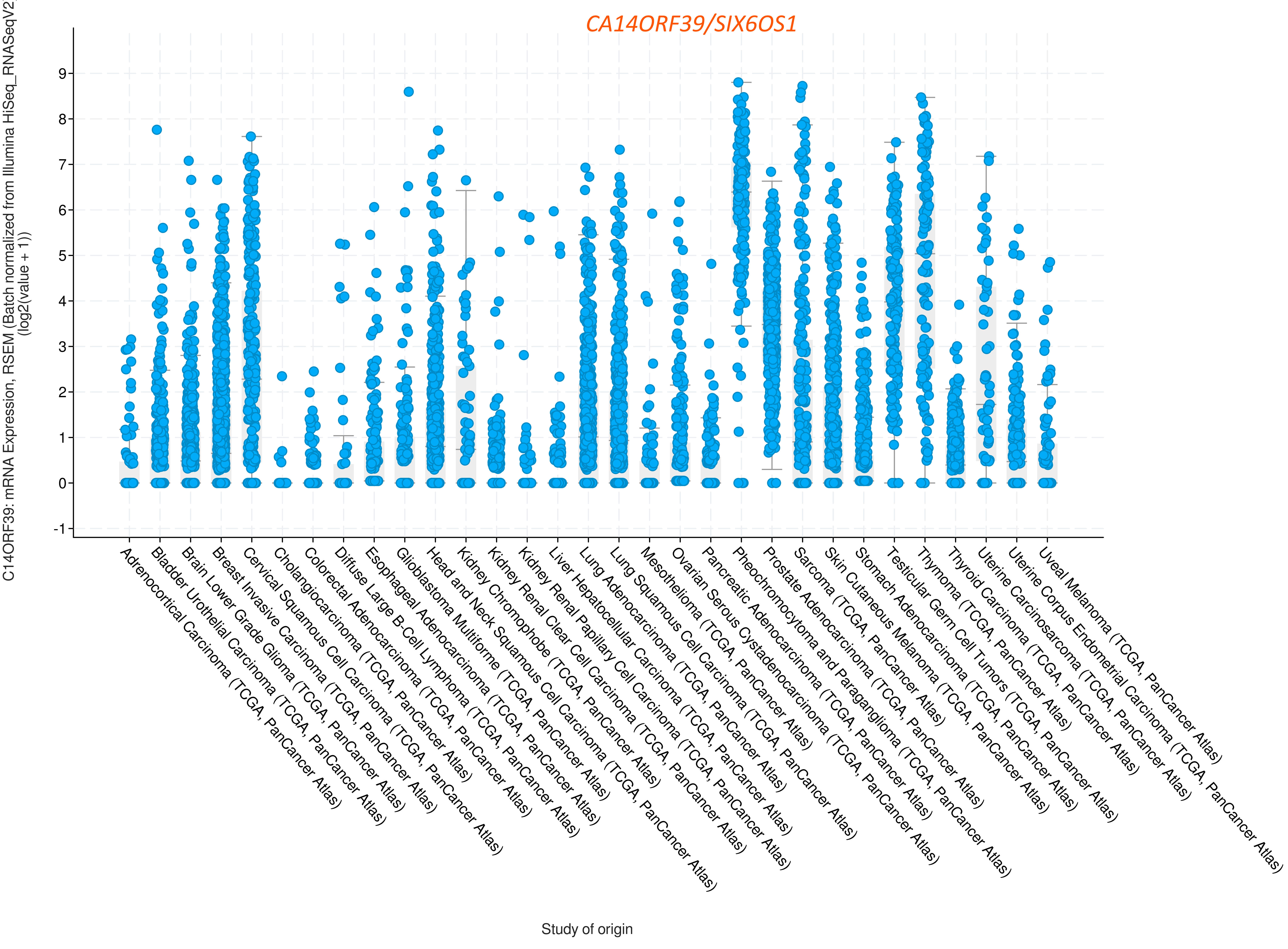

CA14ORF39/SIX6OS1
C14ORF39: mRNA Expression, RSEM (Batch normalized from Illumina HiSeq_RNASeqV2) (log2(value + 1))
9
8
7
6
5
4
3
2
1
0
-1
Adrenocortical Carcinoma (TCGA, PanCancer Atlas)
Bladder Urothelial Carcinoma (TCGA, PanCancer Atlas)
Brain Lower Grade Glioma (TCGA, PanCancer Atlas)
Breast Invasive Carcinoma (TCGA, PanCancer Atlas)
Cervical Squamous Cell Carcinoma (TCGA, PanCancer Atlas)
Cholangiocarcinoma (TCGA, PanCancer Atlas)
Colorectal Adenocarcinoma (TCGA, PanCancer Atlas)
Diffuse Large B-Cell Lymphoma (TCGA, PanCancer Atlas)
Esophageal Adenocarcinoma (TCGA, PanCancer Atlas)
Glioblastoma Multiforme (TCGA, PanCancer Atlas)
Head and Neck Squamous Cell Carcinoma (TCGA, PanCancer Atlas)
Kidney Chromophobe (TCGA, PanCancer Atlas)
Kidney Renal Clear Cell Carcinoma (TCGA, PanCancer Atlas)
Kidney Renal Papillary Cell Carcinoma (TCGA, PanCancer Atlas)
Liver Hepatocellular Carcinoma (TCGA, PanCancer Atlas)
Lung Adenocarcinoma (TCGA, PanCancer Atlas)
Lung Squamous Cell Carcinoma (TCGA, PanCancer Atlas)
Mesothelioma (TCGA, PanCancer Atlas)
Ovarian Serous Cystadenocarcinoma (TCGA, PanCancer Atlas)
Pancreatic Adenocarcinoma (TCGA, PanCancer Atlas)
Pheochromocytoma and Paraganglioma (TCGA, PanCancer Atlas)
Prostate Adenocarcinoma (TCGA, PanCancer Atlas)
Sarcoma (TCGA, PanCancer Atlas)
Skin Cutaneous Melanoma (TCGA, PanCancer Atlas)
Stomach Adenocarcinoma (TCGA, PanCancer Atlas)
Testicular Germ Cell Tumors (TCGA, PanCancer Atlas)
Thymoma (TCGA, PanCancer Atlas)
Thyroid Carcinoma (TCGA, PanCancer Atlas)
Uterine Carcinosarcoma (TCGA, PanCancer Atlas)
Uterine Corpus Endometrial Carcinoma (TCGA, PanCancer Atlas)
Uveal Melanoma (TCGA, PanCancer Atlas)
Study of origin

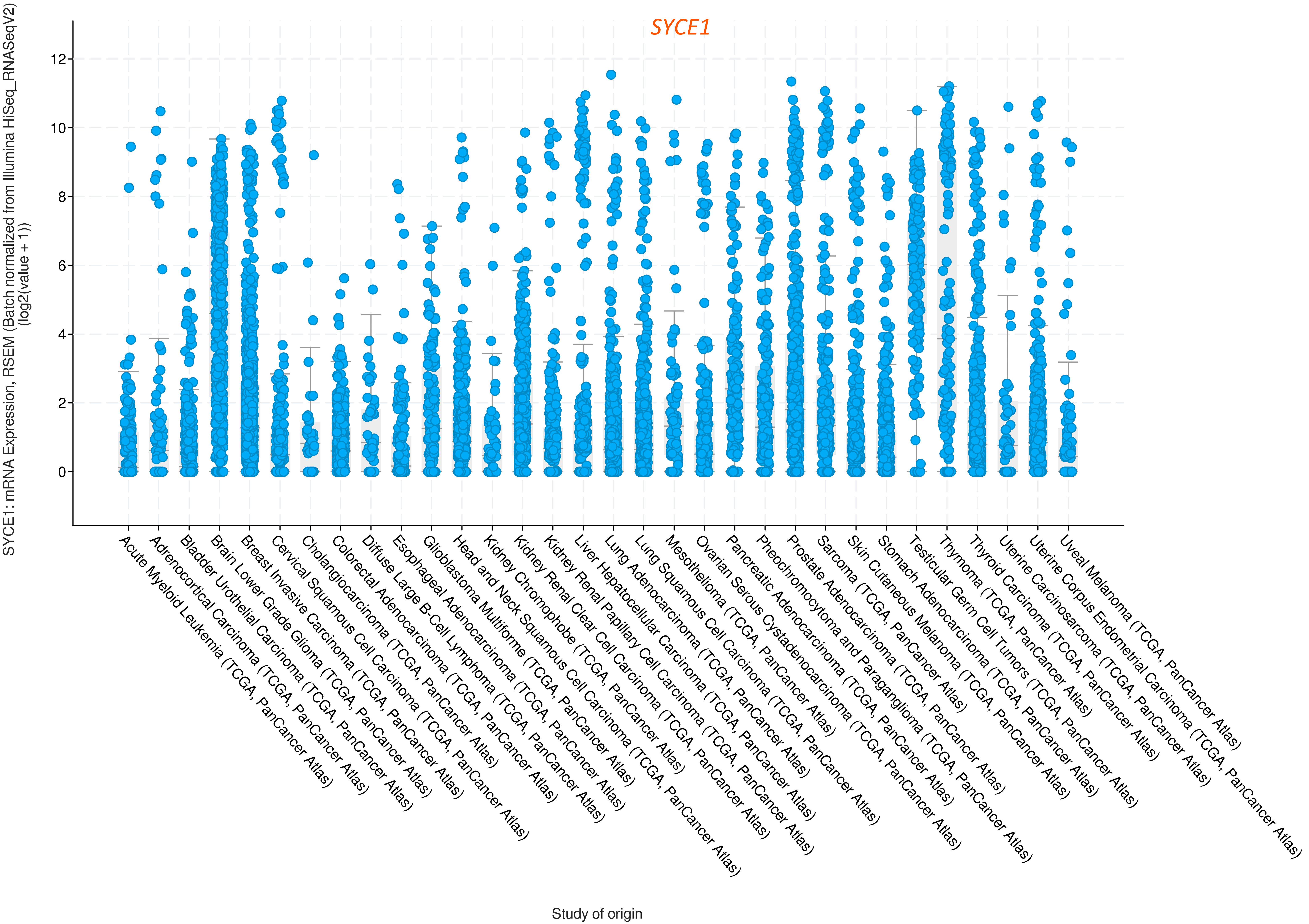

SYCE1
SYCE1: mRNA Expression, RSEM (Batch normalized from Illumina HiSeq_RNASeqV2) (log2(value + 1))
0
2
4
6
8
10
12
Acute Myeloid Leukemia (TCGA, PanCancer Atlas)
Adrenocortical Carcinoma (TCGA, PanCancer Atlas)
Bladder Urothelial Carcinoma (TCGA, PanCancer Atlas)
Brain Lower Grade Glioma (TCGA, PanCancer Atlas)
Breast Invasive Carcinoma (TCGA, PanCancer Atlas)
Cervical Squamous Cell Carcinoma (TCGA, PanCancer Atlas)
Cholangiocarcinoma (TCGA, PanCancer Atlas)
Colorectal Adenocarcinoma (TCGA, PanCancer Atlas)
Diffuse Large B-Cell Lymphoma (TCGA, PanCancer Atlas)
Esophageal Adenocarcinoma (TCGA, PanCancer Atlas)
Glioblastoma Multiforme (TCGA, PanCancer Atlas)
Head and Neck Squamous Cell Carcinoma (TCGA, PanCancer Atlas)
Kidney Chromophobe (TCGA, PanCancer Atlas)
Kidney Renal Clear Cell Carcinoma (TCGA, PanCancer Atlas)
Kidney Renal Papillary Cell Carcinoma (TCGA, PanCancer Atlas)
Liver Hepatocellular Carcinoma (TCGA, PanCancer Atlas)
Lung Adenocarcinoma (TCGA, PanCancer Atlas)
Lung Squamous Cell Carcinoma (TCGA, PanCancer Atlas)
Mesothelioma (TCGA, PanCancer Atlas)
Ovarian Serous Cystadenocarcinoma (TCGA, PanCancer Atlas)
Pancreatic Adenocarcinoma (TCGA, PanCancer Atlas)
Pheochromocytoma and Paraganglioma (TCGA, PanCancer Atlas)
Prostate Adenocarcinoma (TCGA, PanCancer Atlas)
Sarcoma (TCGA, PanCancer Atlas)
Skin Cutaneous Melanoma (TCGA, PanCancer Atlas)
Stomach Adenocarcinoma (TCGA, PanCancer Atlas)
Testicular Germ Cell Tumors (TCGA, PanCancer Atlas)
Thymoma (TCGA, PanCancer Atlas)
Thyroid Carcinoma (TCGA, PanCancer Atlas)
Uterine Carcinosarcoma (TCGA, PanCancer Atlas)
Uterine Corpus Endometrial Carcinoma (TCGA, PanCancer Atlas)
Uveal Melanoma (TCGA, PanCancer Atlas)
Study of origin

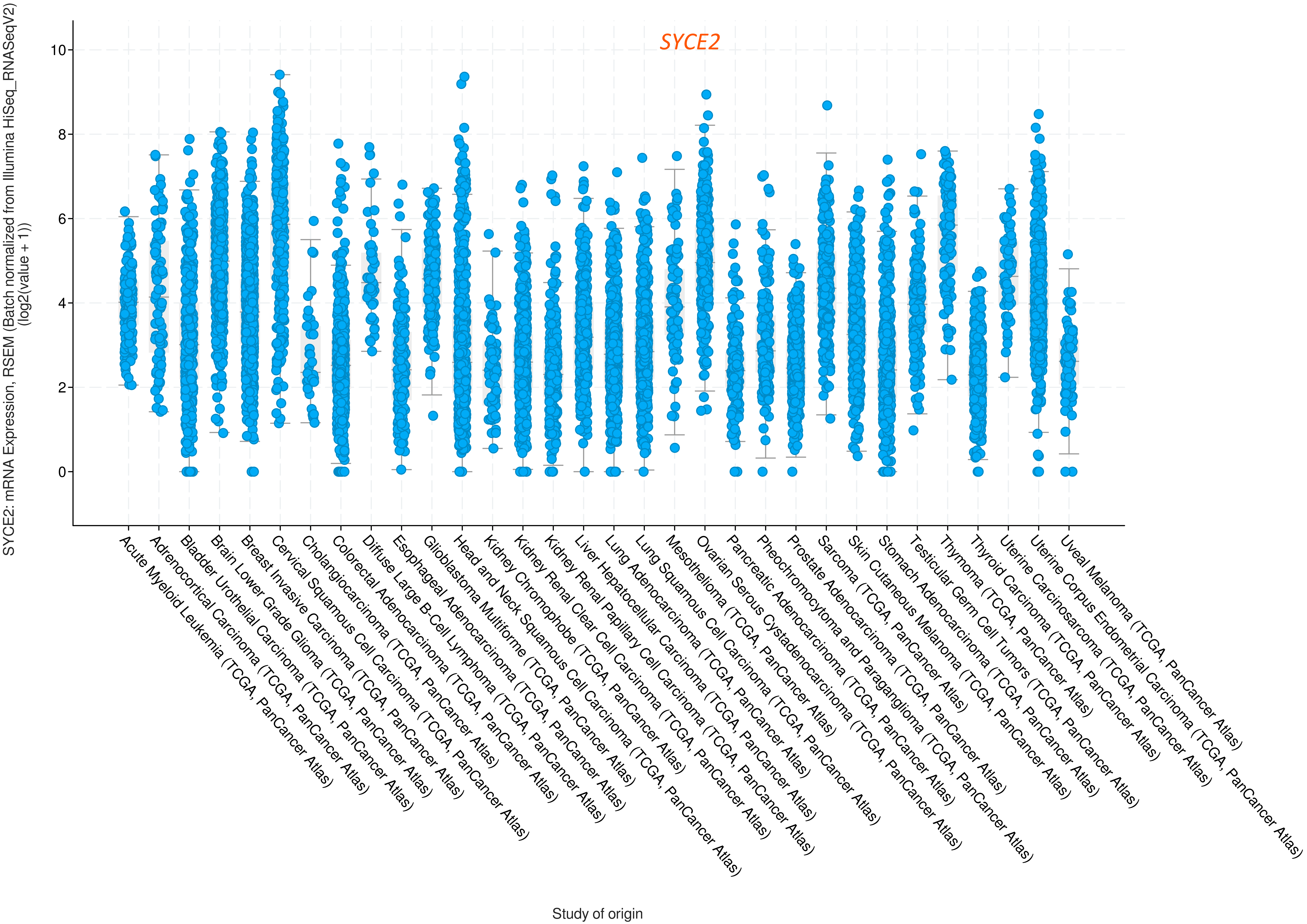

SYCE2
SYCE2: mRNA Expression, RSEM (Batch normalized from Illumina HiSeq_RNASeqV2) (log2(value + 1))
10
8
6
4
2
0
Study of origin
Acute Myeloid Leukemia (TCGA, PanCancer Atlas)
Adrenocortical Carcinoma (TCGA, PanCancer Atlas)
Bladder Urothelial Carcinoma (TCGA, PanCancer Atlas)
Brain Lower Grade Glioma (TCGA, PanCancer Atlas)
Breast Invasive Carcinoma (TCGA, PanCancer Atlas)
Cervical Squamous Cell Carcinoma (TCGA, PanCancer Atlas)
Cholangiocarcinoma (TCGA, PanCancer Atlas)
Colorectal Adenocarcinoma (TCGA, PanCancer Atlas)
Diffuse Large B-Cell Lymphoma (TCGA, PanCancer Atlas)
Esophageal Adenocarcinoma (TCGA, PanCancer Atlas)
Glioblastoma Multiforme (TCGA, PanCancer Atlas)
Head and Neck Squamous Cell Carcinoma (TCGA, PanCancer Atlas)
Kidney Chromophobe (TCGA, PanCancer Atlas)
Kidney Renal Clear Cell Carcinoma (TCGA, PanCancer Atlas)
Kidney Renal Papillary Cell Carcinoma (TCGA, PanCancer Atlas)
Liver Hepatocellular Carcinoma (TCGA, PanCancer Atlas)
Lung Adenocarcinoma (TCGA, PanCancer Atlas)
Lung Squamous Cell Carcinoma (TCGA, PanCancer Atlas)
Mesothelioma (TCGA, PanCancer Atlas)
Ovarian Serous Cystadenocarcinoma (TCGA, PanCancer Atlas)
Pancreatic Adenocarcinoma (TCGA, PanCancer Atlas)
Pheochromocytoma and Paraganglioma (TCGA, PanCancer Atlas)
Prostate Adenocarcinoma (TCGA, PanCancer Atlas)
Sarcoma (TCGA, PanCancer Atlas)
Skin Cutaneous Melanoma (TCGA, PanCancer Atlas)
Stomach Adenocarcinoma (TCGA, PanCancer Atlas)
Testicular Germ Cell Tumors (TCGA, PanCancer Atlas)
Thymoma (TCGA, PanCancer Atlas)
Thyroid Carcinoma (TCGA, PanCancer Atlas)
Uterine Carcinosarcoma (TCGA, PanCancer Atlas)
Uterine Corpus Endometrial Carcinoma (TCGA, PanCancer Atlas)
Uveal Melanoma (TCGA, PanCancer Atlas)

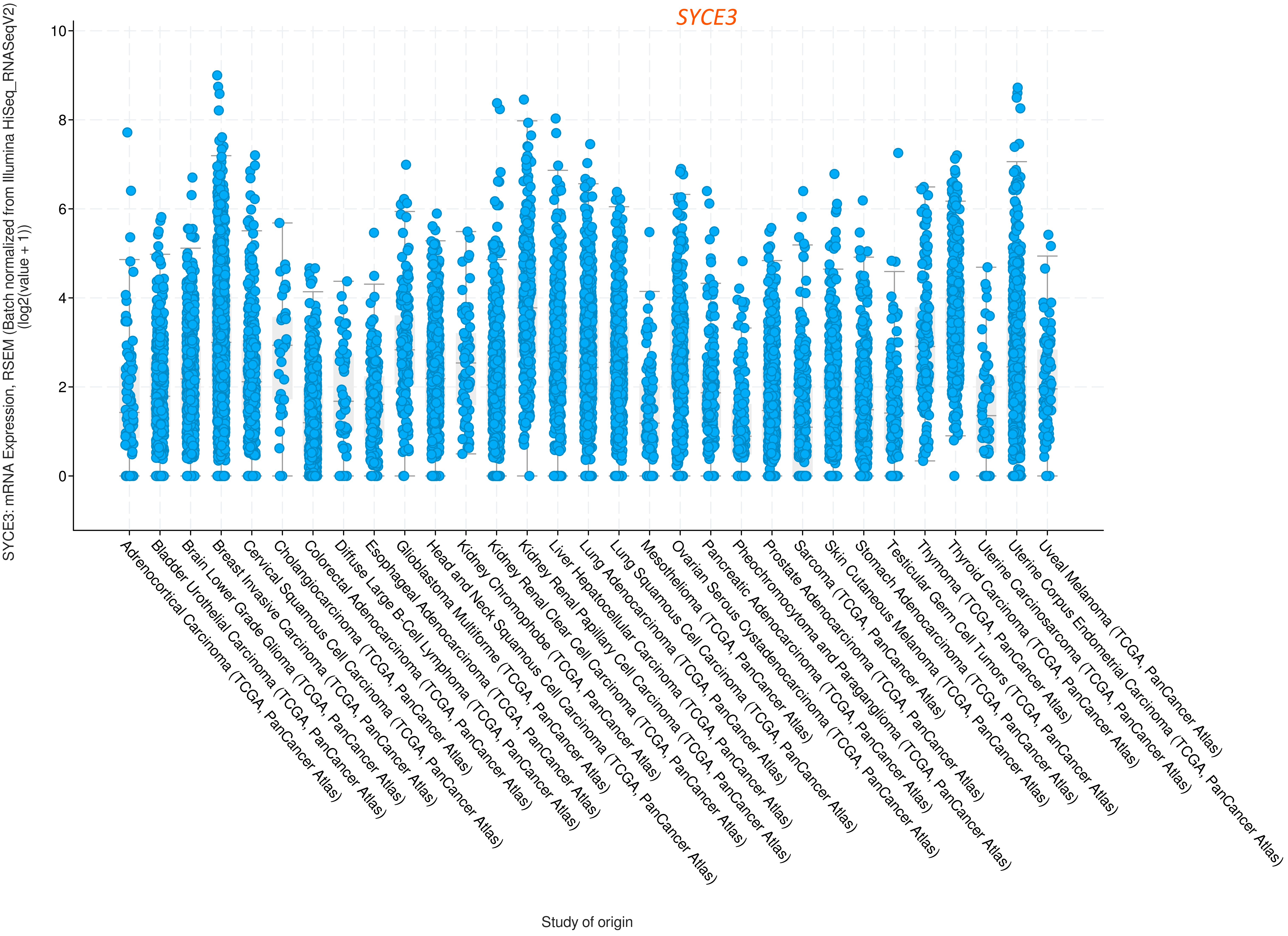

SYCE3
SYCE3: mRNA Expression, RSEM (Batch normalized from Illumina HiSeq_RNASeqV2) (log2(value + 1))
10
8
6
4
2
0
Adrenocortical Carcinoma (TCGA, PanCancer Atlas)
Bladder Urothelial Carcinoma (TCGA, PanCancer Atlas)
Brain Lower Grade Glioma (TCGA, PanCancer Atlas)
Breast Invasive Carcinoma (TCGA, PanCancer Atlas)
Cervical Squamous Cell Carcinoma (TCGA, PanCancer Atlas)
Cholangiocarcinoma (TCGA, PanCancer Atlas)
Colorectal Adenocarcinoma (TCGA, PanCancer Atlas)
Diffuse Large B-Cell Lymphoma (TCGA, PanCancer Atlas)
Esophageal Adenocarcinoma (TCGA, PanCancer Atlas)
Glioblastoma Multiforme (TCGA, PanCancer Atlas)
Head and Neck Squamous Cell Carcinoma (TCGA, PanCancer Atlas)
Kidney Chromophobe (TCGA, PanCancer Atlas)
Kidney Renal Clear Cell Carcinoma (TCGA, PanCancer Atlas)
Kidney Renal Papillary Cell Carcinoma (TCGA, PanCancer Atlas)
Liver Hepatocellular Carcinoma (TCGA, PanCancer Atlas)
Lung Adenocarcinoma (TCGA, PanCancer Atlas)
Lung Squamous Cell Carcinoma (TCGA, PanCancer Atlas)
Mesothelioma (TCGA, PanCancer Atlas)
Ovarian Serous Cystadenocarcinoma (TCGA, PanCancer Atlas)
Pancreatic Adenocarcinoma (TCGA, PanCancer Atlas)
Pheochromocytoma and Paraganglioma (TCGA, PanCancer Atlas)
Prostate Adenocarcinoma (TCGA, PanCancer Atlas)
Sarcoma (TCGA, PanCancer Atlas)
Skin Cutaneous Melanoma (TCGA, PanCancer Atlas)
Stomach Adenocarcinoma (TCGA, PanCancer Atlas)
Testicular Germ Cell Tumors (TCGA, PanCancer Atlas)
Thymoma (TCGA, PanCancer Atlas)
Thyroid Carcinoma (TCGA, PanCancer Atlas)
Uterine Carcinosarcoma (TCGA, PanCancer Atlas)
Uterine Corpus Endometrial Carcinoma (TCGA, PanCancer Atlas)
Uveal Melanoma (TCGA, PanCancer Atlas)
Study of origin

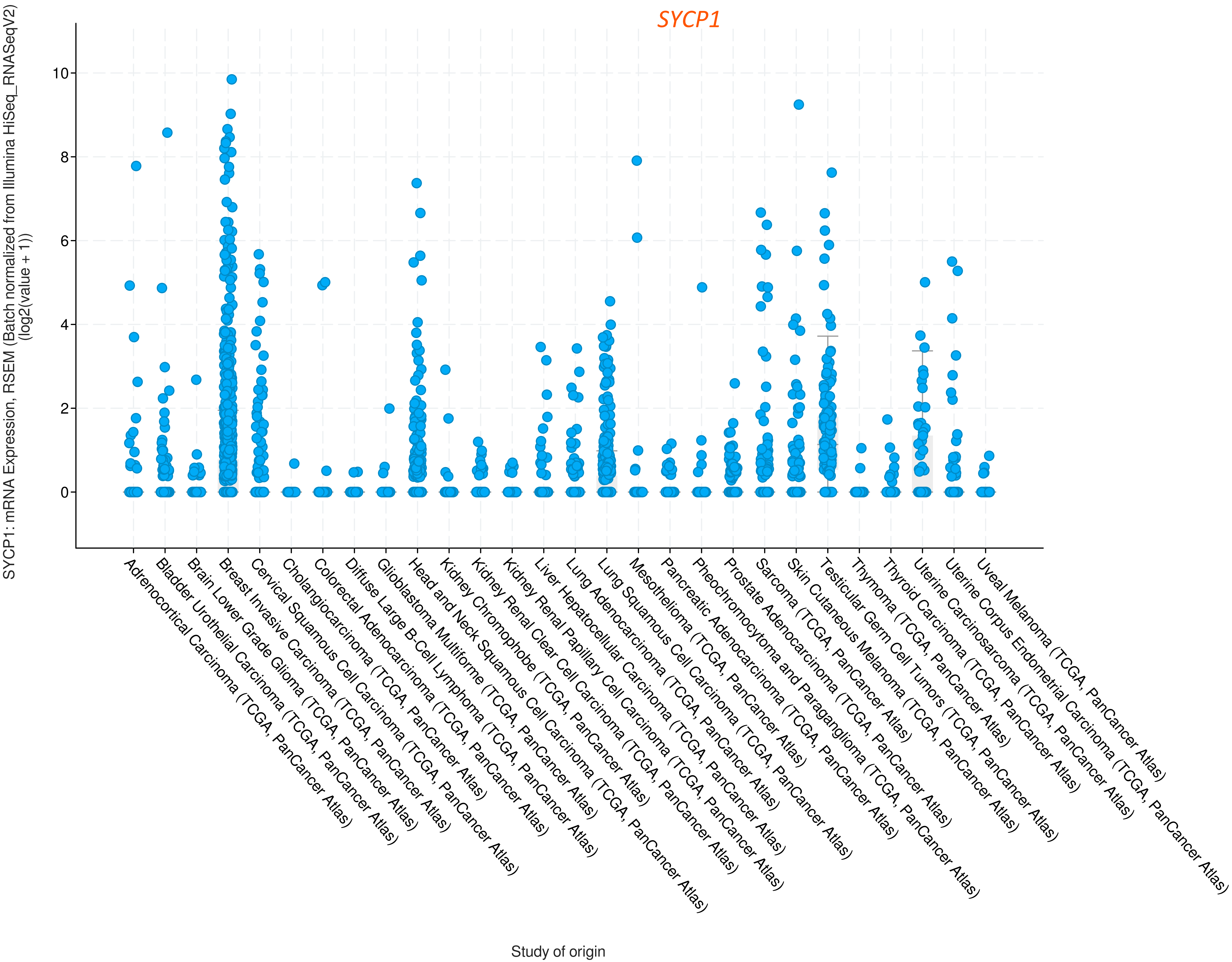
SYCP1
SYCP1: mRNA Expression, RSEM (Batch normalized from Illumina HiSeq_RNASeqV2) (log2(value + 1))
10
8
6
4
2
0
Adrenocortical Carcinoma (TCGA, PanCancer Atlas)
Bladder Urothelial Carcinoma (TCGA, PanCancer Atlas)
Brain Lower Grade Glioma (TCGA, PanCancer Atlas)
Breast Invasive Carcinoma (TCGA, PanCancer Atlas)
Cervical Squamous Cell Carcinoma (TCGA, PanCancer Atlas)
Cholangiocarcinoma (TCGA, PanCancer Atlas)
Colorectal Adenocarcinoma (TCGA, PanCancer Atlas)
Diffuse Large B-Cell Lymphoma (TCGA, PanCancer Atlas)
Glioblastoma Multiforme (TCGA, PanCancer Atlas)
Head and Neck Squamous Cell Carcinoma (TCGA, PanCancer Atlas)
Kidney Chromophobe (TCGA, PanCancer Atlas)
Kidney Renal Clear Cell Carcinoma (TCGA, PanCancer Atlas)
Kidney Renal Papillary Cell Carcinoma (TCGA, PanCancer Atlas)
Liver Hepatocellular Carcinoma (TCGA, PanCancer Atlas)
Lung Adenocarcinoma (TCGA, PanCancer Atlas)
Lung Squamous Cell Carcinoma (TCGA, PanCancer Atlas)
Mesothelioma (TCGA, PanCancer Atlas)
Pancreatic Adenocarcinoma (TCGA, PanCancer Atlas)
Pheochromocytoma and Paraganglioma (TCGA, PanCancer Atlas)
Prostate Adenocarcinoma (TCGA, PanCancer Atlas)
Sarcoma (TCGA, PanCancer Atlas)
Skin Cutaneous Melanoma (TCGA, PanCancer Atlas)
Testicular Germ Cell Tumors (TCGA, PanCancer Atlas)
Thymoma (TCGA, PanCancer Atlas)
Thyroid Carcinoma (TCGA, PanCancer Atlas)
Uterine Carcinosarcoma (TCGA, PanCancer Atlas)
Uterine Corpus Endometrial Carcinoma (TCGA, PanCancer Atlas)
Uveal Melanoma (TCGA, PanCancer Atlas)
Study of origin

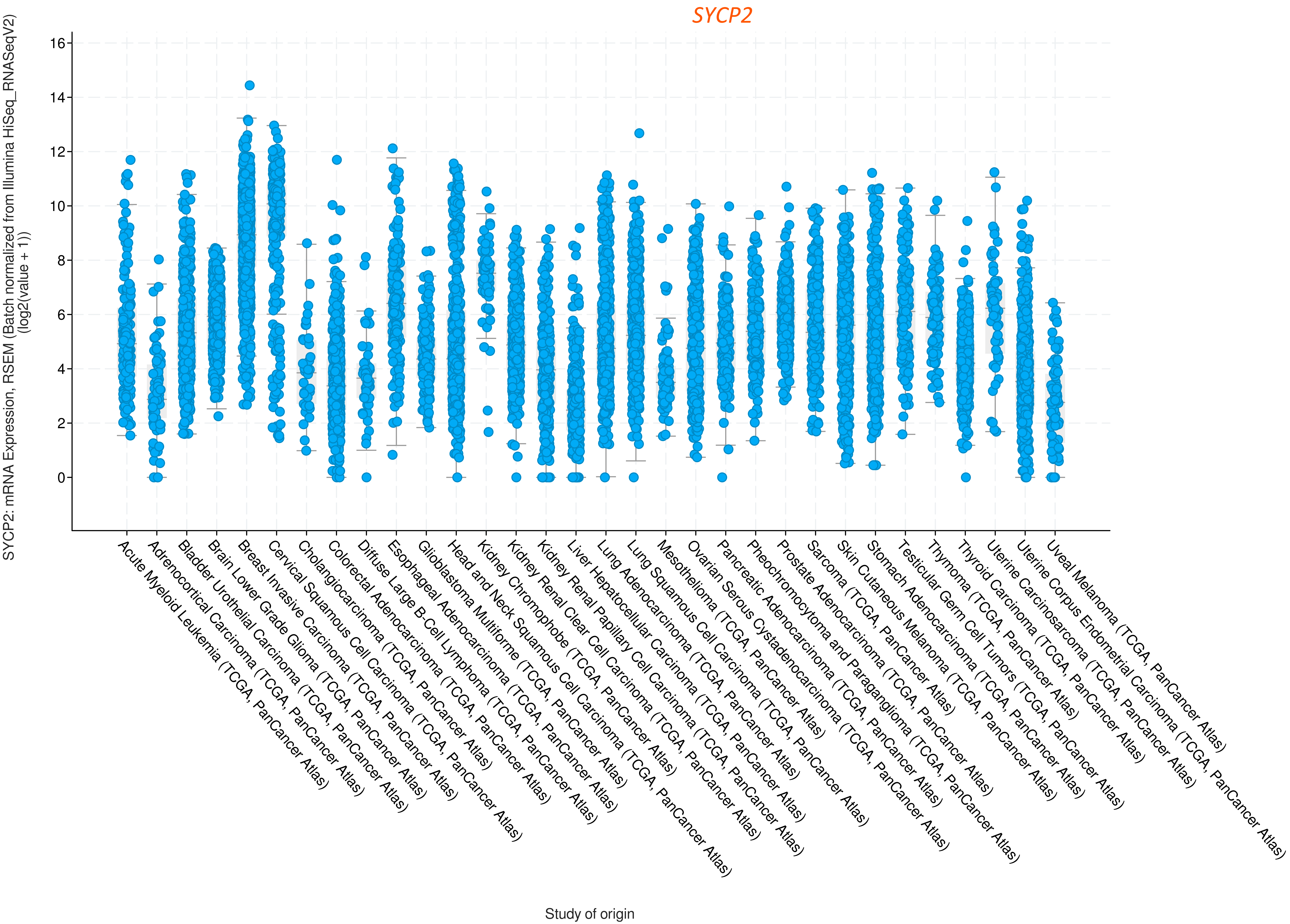

SYCP2
SYCP2: mRNA Expression, RSEM (Batch normalized from Illumina HiSeq_RNASeqV2) (log2(value + 1))
16
14
12
10
8
6
4
2
0
Acute Myeloid Leukemia (TCGA, PanCancer Atlas)
Adrenocortical Carcinoma (TCGA, PanCancer Atlas)
Bladder Urothelial Carcinoma (TCGA, PanCancer Atlas)
Brain Lower Grade Glioma (TCGA, PanCancer Atlas)
Breast Invasive Carcinoma (TCGA, PanCancer Atlas)
Cervical Squamous Cell Carcinoma (TCGA, PanCancer Atlas)
Cholangiocarcinoma (TCGA, PanCancer Atlas)
Colorectal Adenocarcinoma (TCGA, PanCancer Atlas)
Diffuse Large B-Cell Lymphoma (TCGA, PanCancer Atlas)
Esophageal Adenocarcinoma (TCGA, PanCancer Atlas)
Glioblastoma Multiforme (TCGA, PanCancer Atlas)
Head and Neck Squamous Cell Carcinoma (TCGA, PanCancer Atlas)
Kidney Chromophobe (TCGA, PanCancer Atlas)
Kidney Renal Clear Cell Carcinoma (TCGA, PanCancer Atlas)
Kidney Renal Papillary Cell Carcinoma (TCGA, PanCancer Atlas)
Liver Hepatocellular Carcinoma (TCGA, PanCancer Atlas)
Lung Adenocarcinoma (TCGA, PanCancer Atlas)
Lung Squamous Cell Carcinoma (TCGA, PanCancer Atlas)
Mesothelioma (TCGA, PanCancer Atlas)
Ovarian Serous Cystadenocarcinoma (TCGA, PanCancer Atlas)
Pancreatic Adenocarcinoma (TCGA, PanCancer Atlas)
Pheochromocytoma and Paraganglioma (TCGA, PanCancer Atlas)
Prostate Adenocarcinoma (TCGA, PanCancer Atlas)
Sarcoma (TCGA, PanCancer Atlas)
Skin Cutaneous Melanoma (TCGA, PanCancer Atlas)
Stomach Adenocarcinoma (TCGA, PanCancer Atlas)
Testicular Germ Cell Tumors (TCGA, PanCancer Atlas)
Thymoma (TCGA, PanCancer Atlas)
Thyroid Carcinoma (TCGA, PanCancer Atlas)
Uterine Carcinosarcoma (TCGA, PanCancer Atlas)
Uterine Corpus Endometrial Carcinoma (TCGA, PanCancer Atlas)
Uveal Melanoma (TCGA, PanCancer Atlas)
Study of origin

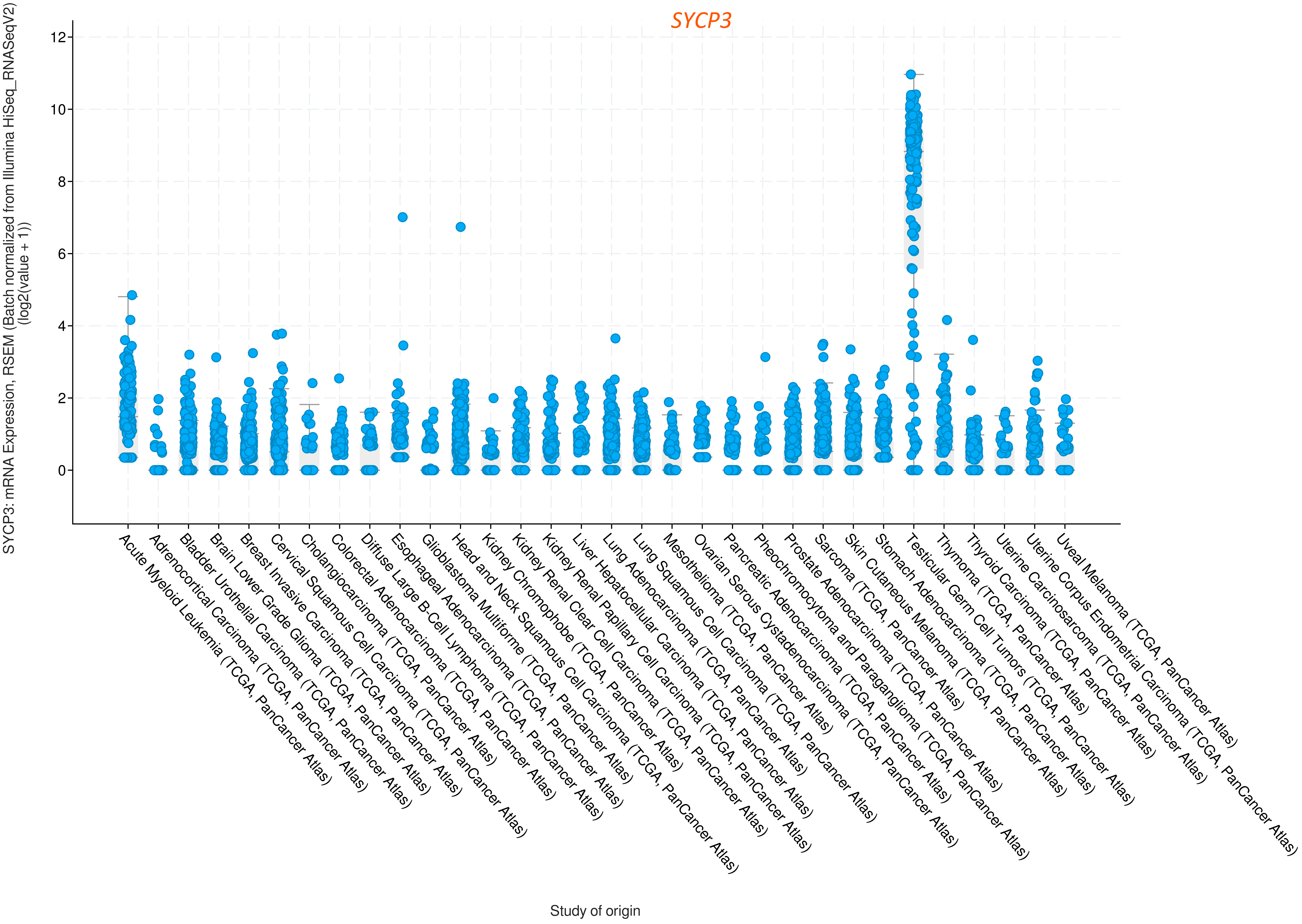
SYCP3
SYCP3: mRNA Expression, RSEM (Batch normalized from Illumina HiSeq_RNASeqV2) (log2(value + 1))
12
10
8
6
4
2
0
Study of origin
Acute Myeloid Leukemia (TCGA, PanCancer Atlas)
Adrenocortical Carcinoma (TCGA, PanCancer Atlas)
Bladder Urothelial Carcinoma (TCGA, PanCancer Atlas)
Brain Lower Grade Glioma (TCGA, PanCancer Atlas)
Breast Invasive Carcinoma (TCGA, PanCancer Atlas)
Cervical Squamous Cell Carcinoma (TCGA, PanCancer Atlas)
Cholangiocarcinoma (TCGA, PanCancer Atlas)
Colorectal Adenocarcinoma (TCGA, PanCancer Atlas)
Diffuse Large B-Cell Lymphoma (TCGA, PanCancer Atlas)
Esophageal Adenocarcinoma (TCGA, PanCancer Atlas)
Glioblastoma Multiforme (TCGA, PanCancer Atlas)
Head and Neck Squamous Cell Carcinoma (TCGA, PanCancer Atlas)
Kidney Chromophobe (TCGA, PanCancer Atlas)
Kidney Renal Clear Cell Carcinoma (TCGA, PanCancer Atlas)
Kidney Renal Papillary Cell Carcinoma (TCGA, PanCancer Atlas)
Liver Hepatocellular Carcinoma (TCGA, PanCancer Atlas)
Lung Adenocarcinoma (TCGA, PanCancer Atlas)
Lung Squamous Cell Carcinoma (TCGA, PanCancer Atlas)
Mesothelioma (TCGA, PanCancer Atlas)
Ovarian Serous Cystadenocarcinoma (TCGA, PanCancer Atlas)
Pancreatic Adenocarcinoma (TCGA, PanCancer Atlas)
Pheochromocytoma and Paraganglioma (TCGA, PanCancer Atlas)
Prostate Adenocarcinoma (TCGA, PanCancer Atlas)
Sarcoma (TCGA, PanCancer Atlas)
Skin Cutaneous Melanoma (TCGA, PanCancer Atlas)
Stomach Adenocarcinoma (TCGA, PanCancer Atlas)
Testicular Germ Cell Tumors (TCGA, PanCancer Atlas)
Thymoma (TCGA, PanCancer Atlas)
Thyroid Carcinoma (TCGA, PanCancer Atlas)
Uterine Carcinosarcoma (TCGA, PanCancer Atlas)
Uterine Corpus Endometrial Carcinoma (TCGA, PanCancer Atlas)
Uveal Melanoma (TCGA, PanCancer Atlas)

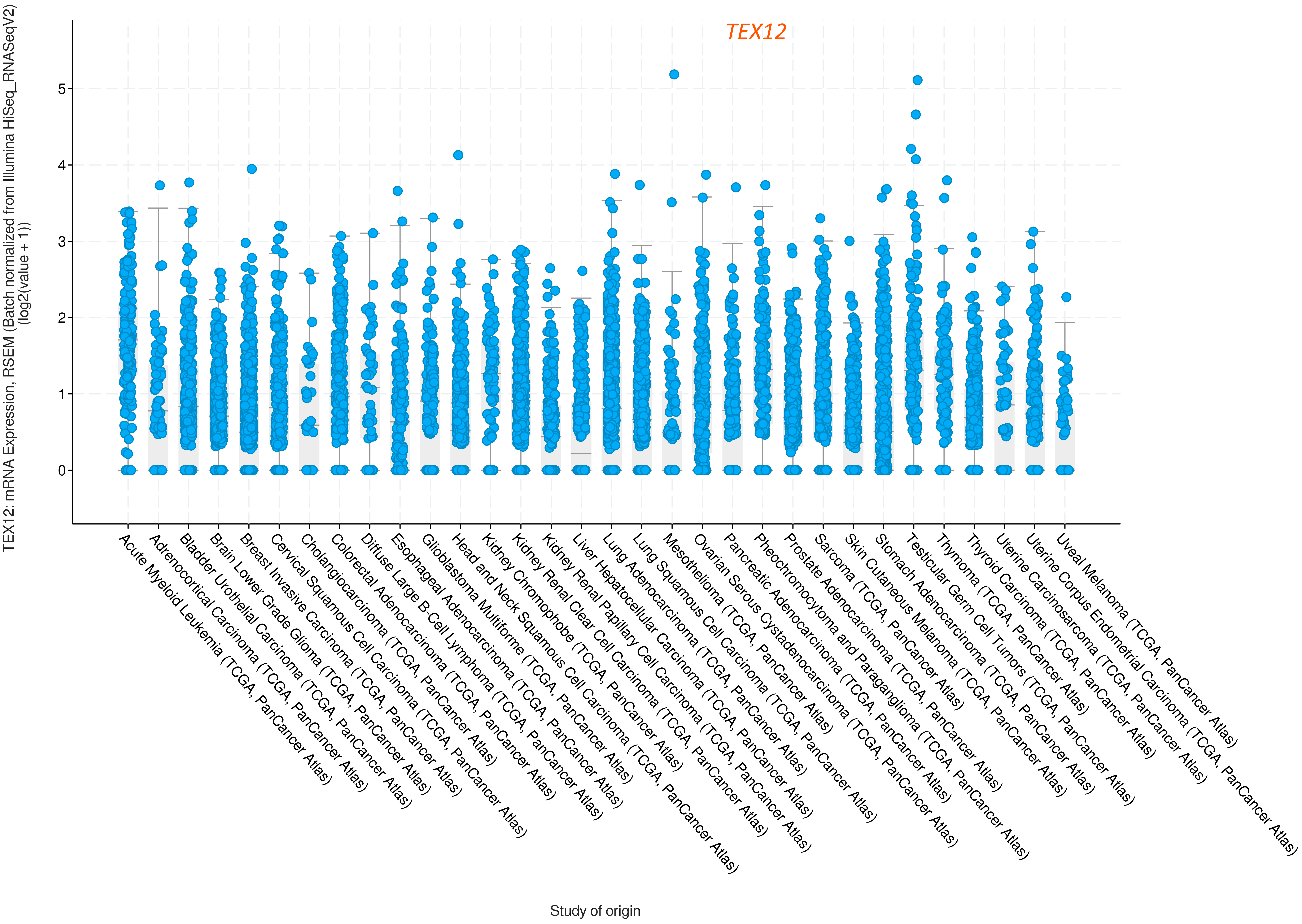

TEX12
TEX12: mRNA Expression, RSEM (Batch normalized from Illumina HiSeq_RNASeqV2) (log2(value + 1))
Study of origin
0
1
2
3
4
5
Acute Myeloid Leukemia (TCGA, PanCancer Atlas)
Adrenocortical Carcinoma (TCGA, PanCancer Atlas)
Bladder Urothelial Carcinoma (TCGA, PanCancer Atlas)
Brain Lower Grade Glioma (TCGA, PanCancer Atlas)
Breast Invasive Carcinoma (TCGA, PanCancer Atlas)
Cervical Squamous Cell Carcinoma (TCGA, PanCancer Atlas)
Cholangiocarcinoma (TCGA, PanCancer Atlas)
Colorectal Adenocarcinoma (TCGA, PanCancer Atlas)
Diffuse Large B-Cell Lymphoma (TCGA, PanCancer Atlas)
Esophageal Adenocarcinoma (TCGA, PanCancer Atlas)
Glioblastoma Multiforme (TCGA, PanCancer Atlas)
Head and Neck Squamous Cell Carcinoma (TCGA, PanCancer Atlas)
Kidney Chromophobe (TCGA, PanCancer Atlas)
Kidney Renal Clear Cell Carcinoma (TCGA, PanCancer Atlas)
Kidney Renal Papillary Cell Carcinoma (TCGA, PanCancer Atlas)
Liver Hepatocellular Carcinoma (TCGA, PanCancer Atlas)
Lung Adenocarcinoma (TCGA, PanCancer Atlas)
Lung Squamous Cell Carcinoma (TCGA, PanCancer Atlas)
Mesothelioma (TCGA, PanCancer Atlas)
Ovarian Serous Cystadenocarcinoma (TCGA, PanCancer Atlas)
Pancreatic Adenocarcinoma (TCGA, PanCancer Atlas)
Pheochromocytoma and Paraganglioma (TCGA, PanCancer Atlas)
Prostate Adenocarcinoma (TCGA, PanCancer Atlas)
Sarcoma (TCGA, PanCancer Atlas)
Skin Cutaneous Melanoma (TCGA, PanCancer Atlas)
Stomach Adenocarcinoma (TCGA, PanCancer Atlas)
Testicular Germ Cell Tumors (TCGA, PanCancer Atlas)
Thymoma (TCGA, PanCancer Atlas)
Thyroid Carcinoma (TCGA, PanCancer Atlas)
Uterine Carcinosarcoma (TCGA, PanCancer Atlas)
Uterine Corpus Endometrial Carcinoma (TCGA, PanCancer Atlas)
Uveal Melanoma (TCGA, PanCancer Atlas)

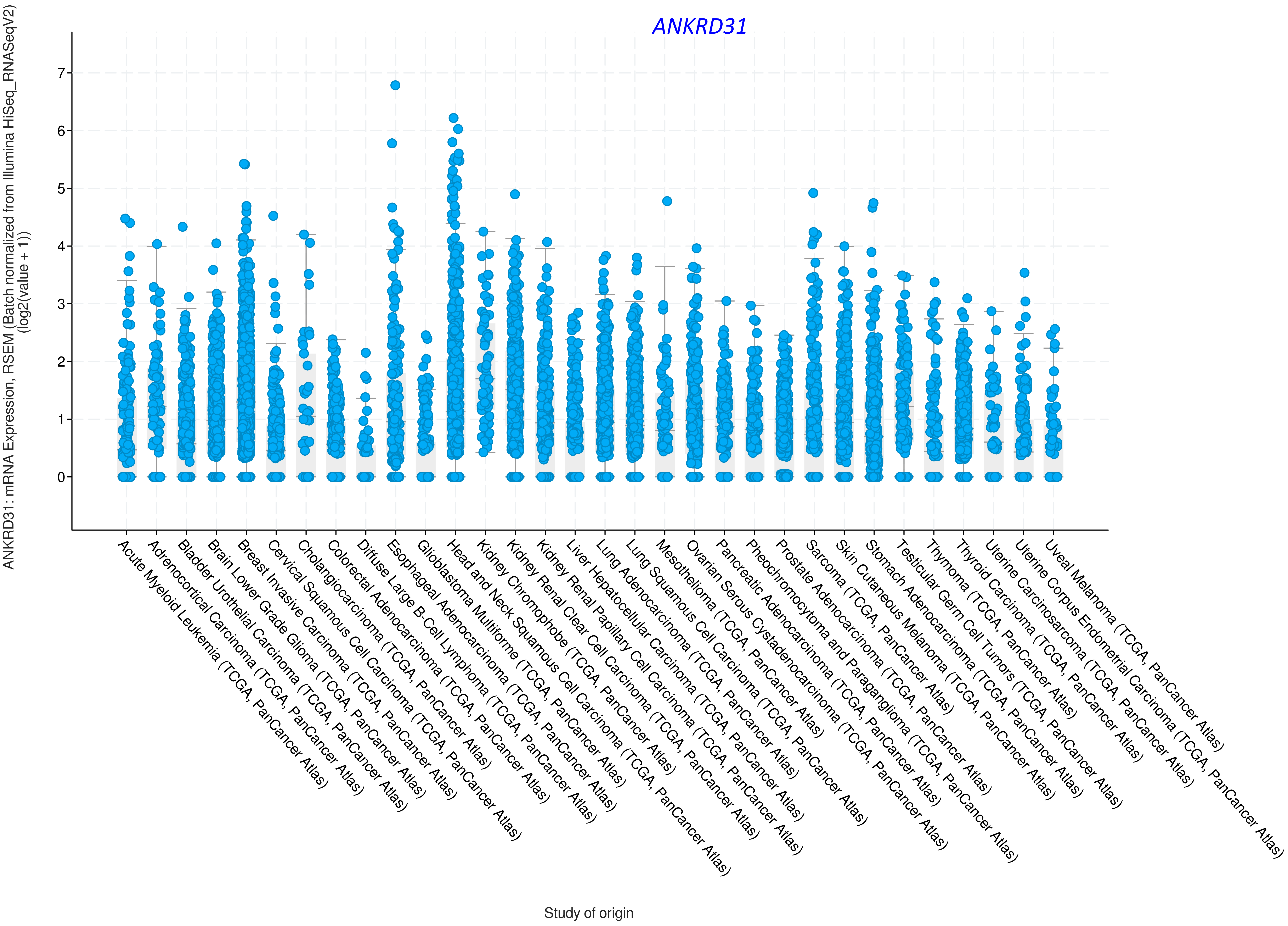

ANKRD31
ANKRD31: mRNA Expression, RSEM (Batch normalized from Illumina HiSeq_RNASeqV2) (log2(value + 1))
Study of origin
0
1
2
3
4
5
6
7
Acute Myeloid Leukemia (TCGA, PanCancer Atlas)
Adrenocortical Carcinoma (TCGA, PanCancer Atlas)
Bladder Urothelial Carcinoma (TCGA, PanCancer Atlas)
Brain Lower Grade Glioma (TCGA, PanCancer Atlas)
Breast Invasive Carcinoma (TCGA, PanCancer Atlas)
Cervical Squamous Cell Carcinoma (TCGA, PanCancer Atlas)
Cholangiocarcinoma (TCGA, PanCancer Atlas)
Colorectal Adenocarcinoma (TCGA, PanCancer Atlas)
Diffuse Large B-Cell Lymphoma (TCGA, PanCancer Atlas)
Esophageal Adenocarcinoma (TCGA, PanCancer Atlas)
Glioblastoma Multiforme (TCGA, PanCancer Atlas)
Head and Neck Squamous Cell Carcinoma (TCGA, PanCancer Atlas)
Kidney Chromophobe (TCGA, PanCancer Atlas)
Kidney Renal Clear Cell Carcinoma (TCGA, PanCancer Atlas)
Kidney Renal Papillary Cell Carcinoma (TCGA, PanCancer Atlas)
Liver Hepatocellular Carcinoma (TCGA, PanCancer Atlas)
Lung Adenocarcinoma (TCGA, PanCancer Atlas)
Lung Squamous Cell Carcinoma (TCGA, PanCancer Atlas)
Mesothelioma (TCGA, PanCancer Atlas)
Ovarian Serous Cystadenocarcinoma (TCGA, PanCancer Atlas)
Pancreatic Adenocarcinoma (TCGA, PanCancer Atlas)
Pheochromocytoma and Paraganglioma (TCGA, PanCancer Atlas)
Prostate Adenocarcinoma (TCGA, PanCancer Atlas)
Sarcoma (TCGA, PanCancer Atlas)
Skin Cutaneous Melanoma (TCGA, PanCancer Atlas)
Stomach Adenocarcinoma (TCGA, PanCancer Atlas)
Testicular Germ Cell Tumors (TCGA, PanCancer Atlas)
Thymoma (TCGA, PanCancer Atlas)
Thyroid Carcinoma (TCGA, PanCancer Atlas)
Uterine Carcinosarcoma (TCGA, PanCancer Atlas)
Uterine Corpus Endometrial Carcinoma (TCGA, PanCancer Atlas)
Uveal Melanoma (TCGA, PanCancer Atlas)

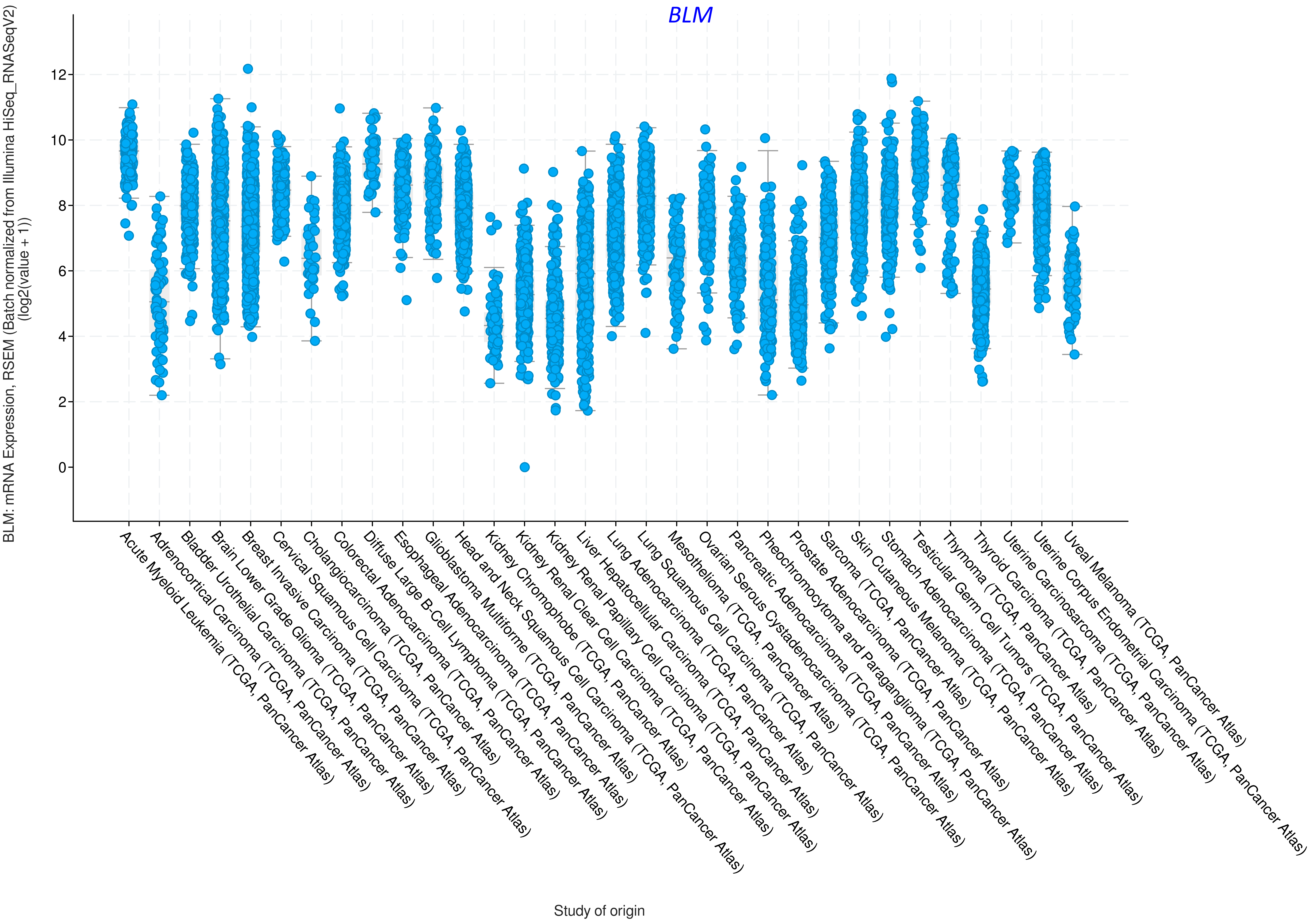

BLM
BLM: mRNA Expression, RSEM (Batch normalized from Illumina HiSeq_RNASeqV2) (log2(value + 1))
12
10
8
6
4
2
0
Study of origin
Acute Myeloid Leukemia (TCGA, PanCancer Atlas)
Adrenocortical Carcinoma (TCGA, PanCancer Atlas)
Bladder Urothelial Carcinoma (TCGA, PanCancer Atlas)
Brain Lower Grade Glioma (TCGA, PanCancer Atlas)
Breast Invasive Carcinoma (TCGA, PanCancer Atlas)
Cervical Squamous Cell Carcinoma (TCGA, PanCancer Atlas)
Cholangiocarcinoma (TCGA, PanCancer Atlas)
Colorectal Adenocarcinoma (TCGA, PanCancer Atlas)
Diffuse Large B-Cell Lymphoma (TCGA, PanCancer Atlas)
Esophageal Adenocarcinoma (TCGA, PanCancer Atlas)
Glioblastoma Multiforme (TCGA, PanCancer Atlas)
Head and Neck Squamous Cell Carcinoma (TCGA, PanCancer Atlas)
Kidney Chromophobe (TCGA, PanCancer Atlas)
Kidney Renal Clear Cell Carcinoma (TCGA, PanCancer Atlas)
Kidney Renal Papillary Cell Carcinoma (TCGA, PanCancer Atlas)
Liver Hepatocellular Carcinoma (TCGA, PanCancer Atlas)
Lung Adenocarcinoma (TCGA, PanCancer Atlas)
Lung Squamous Cell Carcinoma (TCGA, PanCancer Atlas)
Mesothelioma (TCGA, PanCancer Atlas)
Ovarian Serous Cystadenocarcinoma (TCGA, PanCancer Atlas)
Pancreatic Adenocarcinoma (TCGA, PanCancer Atlas)
Pheochromocytoma and Paraganglioma (TCGA, PanCancer Atlas)
Prostate Adenocarcinoma (TCGA, PanCancer Atlas)
Sarcoma (TCGA, PanCancer Atlas)
Skin Cutaneous Melanoma (TCGA, PanCancer Atlas)
Stomach Adenocarcinoma (TCGA, PanCancer Atlas)
Testicular Germ Cell Tumors (TCGA, PanCancer Atlas)
Thymoma (TCGA, PanCancer Atlas)
Thyroid Carcinoma (TCGA, PanCancer Atlas)
Uterine Carcinosarcoma (TCGA, PanCancer Atlas)
Uterine Corpus Endometrial Carcinoma (TCGA, PanCancer Atlas)
Uveal Melanoma (TCGA, PanCancer Atlas)

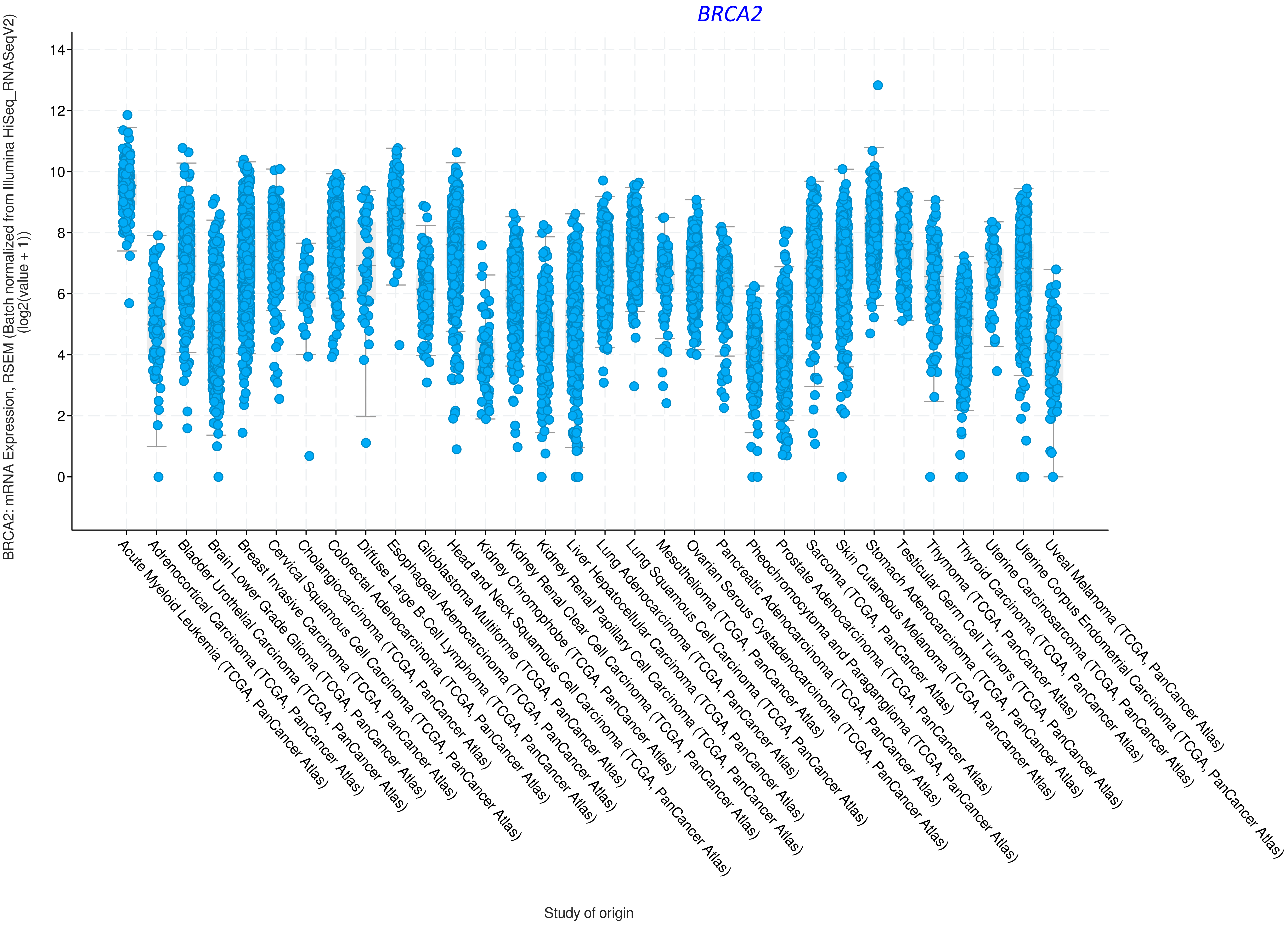

BRCA2
BRCA2: mRNA Expression, RSEM (Batch normalized from Illumina HiSeq_RNASeqV2) (log2(value + 1))
0
2
4
6
8
10
12
14
Study of origin
Acute Myeloid Leukemia (TCGA, PanCancer Atlas)
Adrenocortical Carcinoma (TCGA, PanCancer Atlas)
Bladder Urothelial Carcinoma (TCGA, PanCancer Atlas)
Brain Lower Grade Glioma (TCGA, PanCancer Atlas)
Breast Invasive Carcinoma (TCGA, PanCancer Atlas)
Cervical Squamous Cell Carcinoma (TCGA, PanCancer Atlas)
Cholangiocarcinoma (TCGA, PanCancer Atlas)
Colorectal Adenocarcinoma (TCGA, PanCancer Atlas)
Diffuse Large B-Cell Lymphoma (TCGA, PanCancer Atlas)
Esophageal Adenocarcinoma (TCGA, PanCancer Atlas)
Glioblastoma Multiforme (TCGA, PanCancer Atlas)
Head and Neck Squamous Cell Carcinoma (TCGA, PanCancer Atlas)
Kidney Chromophobe (TCGA, PanCancer Atlas)
Kidney Renal Clear Cell Carcinoma (TCGA, PanCancer Atlas)
Kidney Renal Papillary Cell Carcinoma (TCGA, PanCancer Atlas)
Liver Hepatocellular Carcinoma (TCGA, PanCancer Atlas)
Lung Adenocarcinoma (TCGA, PanCancer Atlas)
Lung Squamous Cell Carcinoma (TCGA, PanCancer Atlas)
Mesothelioma (TCGA, PanCancer Atlas)
Ovarian Serous Cystadenocarcinoma (TCGA, PanCancer Atlas)
Pancreatic Adenocarcinoma (TCGA, PanCancer Atlas)
Pheochromocytoma and Paraganglioma (TCGA, PanCancer Atlas)
Prostate Adenocarcinoma (TCGA, PanCancer Atlas)
Sarcoma (TCGA, PanCancer Atlas)
Skin Cutaneous Melanoma (TCGA, PanCancer Atlas)
Stomach Adenocarcinoma (TCGA, PanCancer Atlas)
Testicular Germ Cell Tumors (TCGA, PanCancer Atlas)
Thymoma (TCGA, PanCancer Atlas)
Thyroid Carcinoma (TCGA, PanCancer Atlas)
Uterine Carcinosarcoma (TCGA, PanCancer Atlas)
Uterine Corpus Endometrial Carcinoma (TCGA, PanCancer Atlas)
Uveal Melanoma (TCGA, PanCancer Atlas)

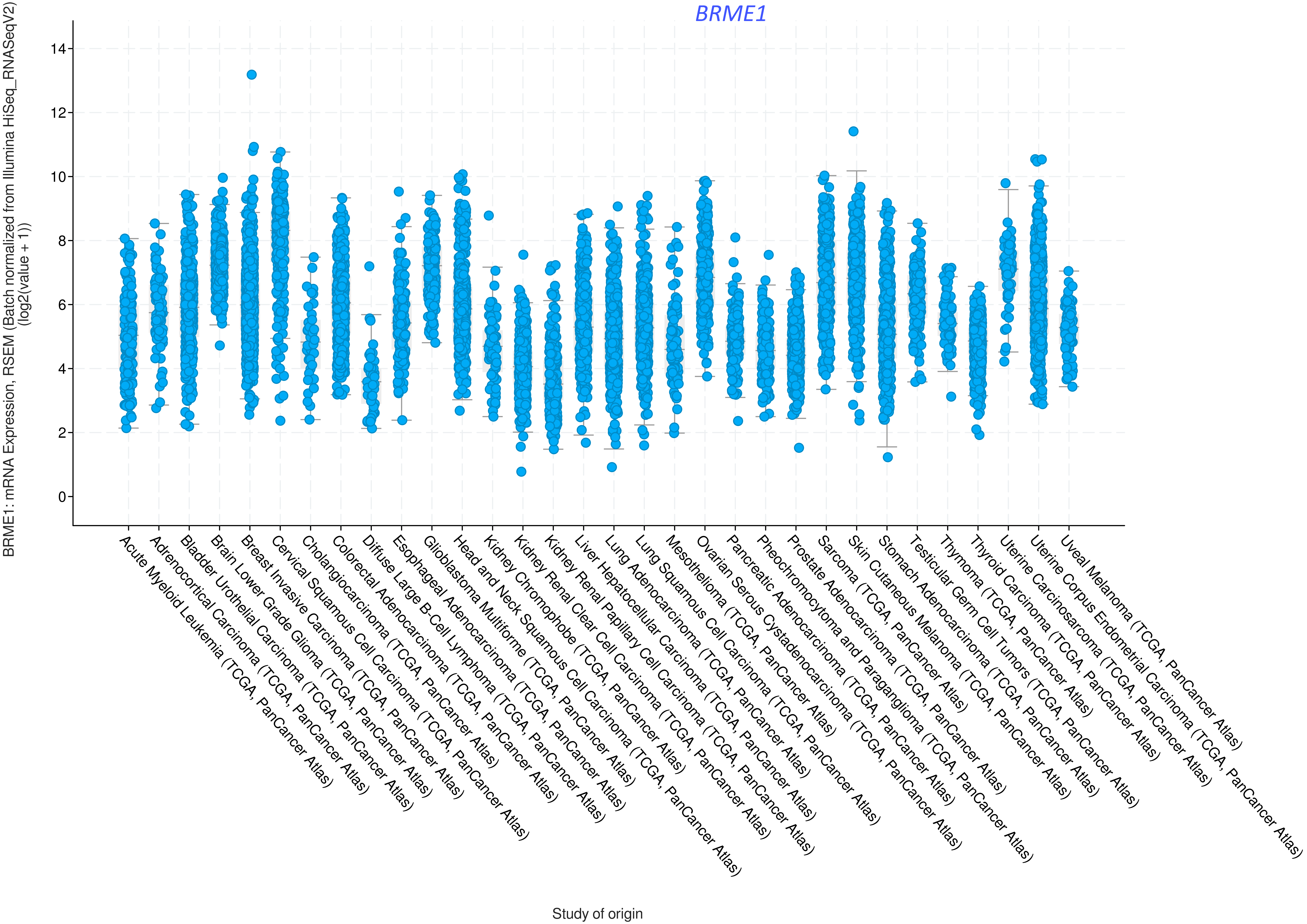

BRME1
BRME1: mRNA Expression, RSEM (Batch normalized from Illumina HiSeq_RNASeqV2) (log2(value + 1))
14
12
10
8
6
4
2
0
Acute Myeloid Leukemia (TCGA, PanCancer Atlas)
Adrenocortical Carcinoma (TCGA, PanCancer Atlas)
Bladder Urothelial Carcinoma (TCGA, PanCancer Atlas)
Brain Lower Grade Glioma (TCGA, PanCancer Atlas)
Breast Invasive Carcinoma (TCGA, PanCancer Atlas)
Cervical Squamous Cell Carcinoma (TCGA, PanCancer Atlas)
Cholangiocarcinoma (TCGA, PanCancer Atlas)
Colorectal Adenocarcinoma (TCGA, PanCancer Atlas)
Diffuse Large B-Cell Lymphoma (TCGA, PanCancer Atlas)
Esophageal Adenocarcinoma (TCGA, PanCancer Atlas)
Glioblastoma Multiforme (TCGA, PanCancer Atlas)
Head and Neck Squamous Cell Carcinoma (TCGA, PanCancer Atlas)
Kidney Chromophobe (TCGA, PanCancer Atlas)
Kidney Renal Clear Cell Carcinoma (TCGA, PanCancer Atlas)
Kidney Renal Papillary Cell Carcinoma (TCGA, PanCancer Atlas)
Liver Hepatocellular Carcinoma (TCGA, PanCancer Atlas)
Lung Adenocarcinoma (TCGA, PanCancer Atlas)
Lung Squamous Cell Carcinoma (TCGA, PanCancer Atlas)
Mesothelioma (TCGA, PanCancer Atlas)
Ovarian Serous Cystadenocarcinoma (TCGA, PanCancer Atlas)
Pancreatic Adenocarcinoma (TCGA, PanCancer Atlas)
Pheochromocytoma and Paraganglioma (TCGA, PanCancer Atlas)
Prostate Adenocarcinoma (TCGA, PanCancer Atlas)
Sarcoma (TCGA, PanCancer Atlas)
Skin Cutaneous Melanoma (TCGA, PanCancer Atlas)
Stomach Adenocarcinoma (TCGA, PanCancer Atlas)
Testicular Germ Cell Tumors (TCGA, PanCancer Atlas)
Thymoma (TCGA, PanCancer Atlas)
Thyroid Carcinoma (TCGA, PanCancer Atlas)
Uterine Carcinosarcoma (TCGA, PanCancer Atlas)
Uterine Corpus Endometrial Carcinoma (TCGA, PanCancer Atlas)
Uveal Melanoma (TCGA, PanCancer Atlas)
Study of origin

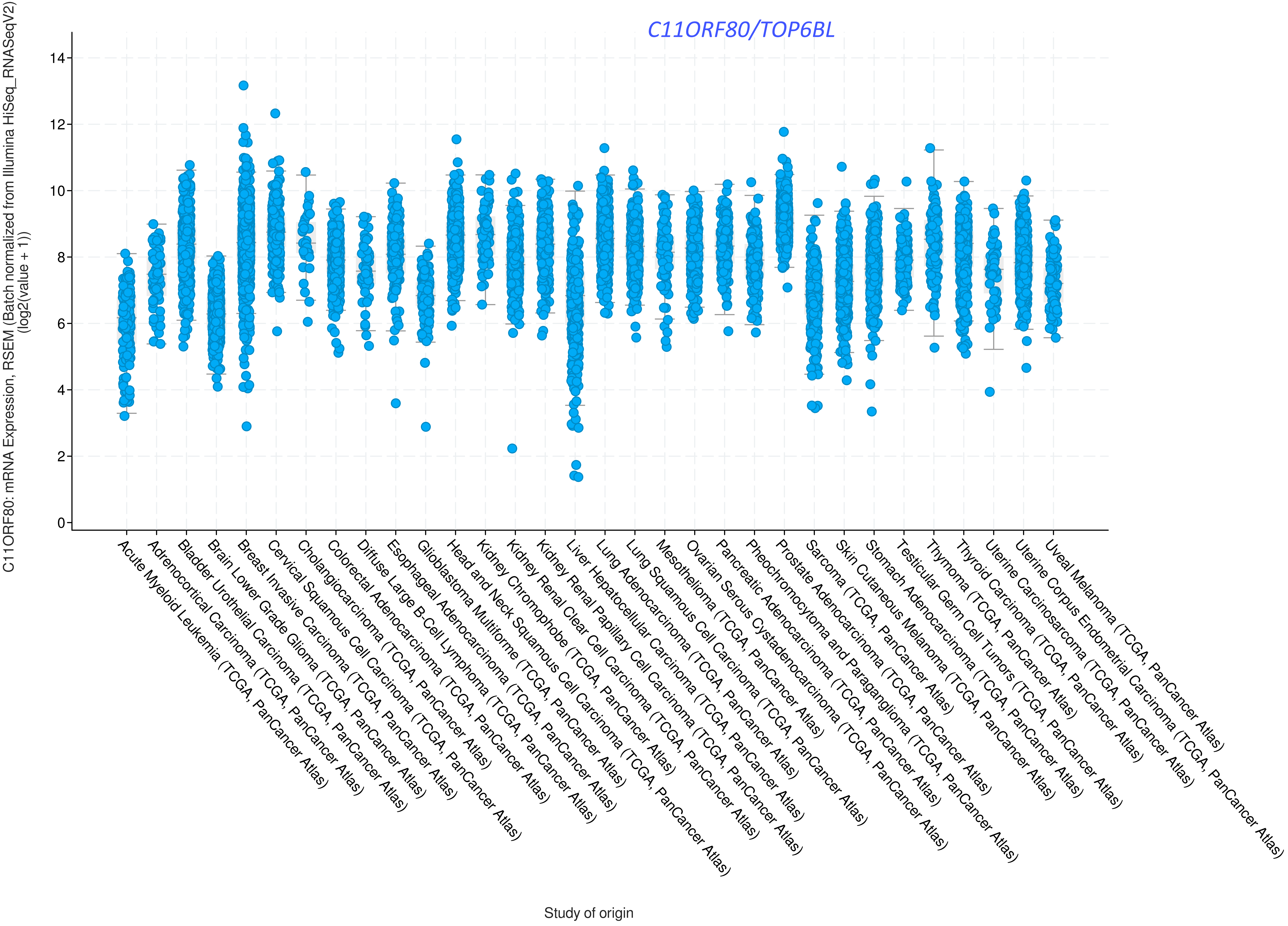
C11ORF80/TOP6BL
C11ORF80: mRNA Expression, RSEM (Batch normalized from Illumina HiSeq_RNASeqV2) (log2(value + 1))
Study of origin
Acute Myeloid Leukemia (TCGA, PanCancer Atlas)
Adrenocortical Carcinoma (TCGA, PanCancer Atlas)
Bladder Urothelial Carcinoma (TCGA, PanCancer Atlas)
Brain Lower Grade Glioma (TCGA, PanCancer Atlas)
Breast Invasive Carcinoma (TCGA, PanCancer Atlas)
Cervical Squamous Cell Carcinoma (TCGA, PanCancer Atlas)
Cholangiocarcinoma (TCGA, PanCancer Atlas)
Colorectal Adenocarcinoma (TCGA, PanCancer Atlas)
Diffuse Large B-Cell Lymphoma (TCGA, PanCancer Atlas)
Esophageal Adenocarcinoma (TCGA, PanCancer Atlas)
Glioblastoma Multiforme (TCGA, PanCancer Atlas)
Head and Neck Squamous Cell Carcinoma (TCGA, PanCancer Atlas)
Kidney Chromophobe (TCGA, PanCancer Atlas)
Kidney Renal Clear Cell Carcinoma (TCGA, PanCancer Atlas)
Kidney Renal Papillary Cell Carcinoma (TCGA, PanCancer Atlas)
Liver Hepatocellular Carcinoma (TCGA, PanCancer Atlas)
Lung Adenocarcinoma (TCGA, PanCancer Atlas)
Lung Squamous Cell Carcinoma (TCGA, PanCancer Atlas)
Mesothelioma (TCGA, PanCancer Atlas)
Ovarian Serous Cystadenocarcinoma (TCGA, PanCancer Atlas)
Pancreatic Adenocarcinoma (TCGA, PanCancer Atlas)
Pheochromocytoma and Paraganglioma (TCGA, PanCancer Atlas)
Prostate Adenocarcinoma (TCGA, PanCancer Atlas)
Sarcoma (TCGA, PanCancer Atlas)
Skin Cutaneous Melanoma (TCGA, PanCancer Atlas)
Stomach Adenocarcinoma (TCGA, PanCancer Atlas)
Testicular Germ Cell Tumors (TCGA, PanCancer Atlas)
Thymoma (TCGA, PanCancer Atlas)
Thyroid Carcinoma (TCGA, PanCancer Atlas)
Uterine Carcinosarcoma (TCGA, PanCancer Atlas)
Uterine Corpus Endometrial Carcinoma (TCGA, PanCancer Atlas)
Uveal Melanoma (TCGA, PanCancer Atlas)
0
2
4
6
8
10
12
14

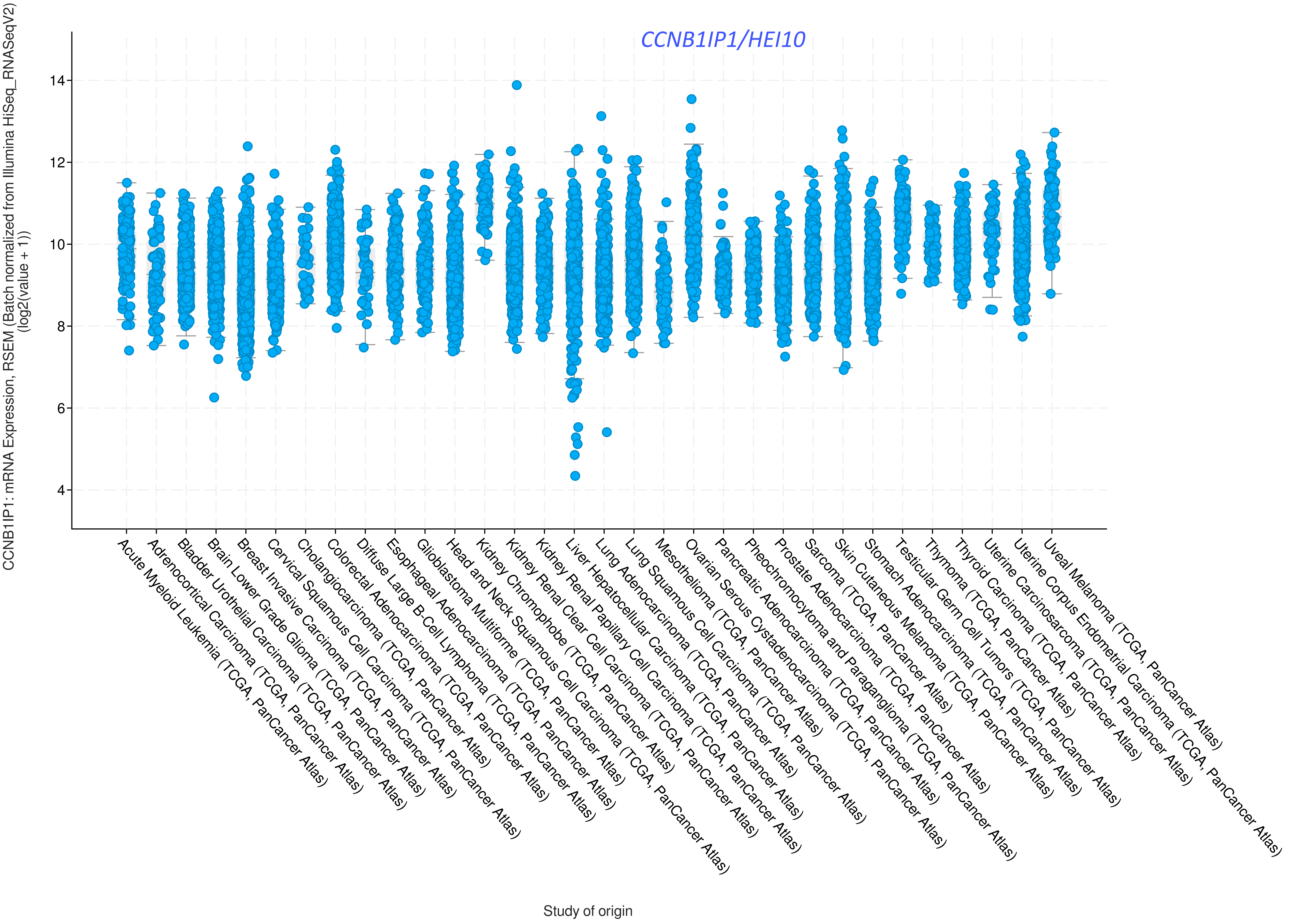

CCNB1IP1/HEI10
CCNB1IP1: mRNA Expression, RSEM (Batch normalized from Illumina HiSeq_RNASeqV2) (log2(value + 1))
4
6
8
10
12
14
Acute Myeloid Leukemia (TCGA, PanCancer Atlas)
Adrenocortical Carcinoma (TCGA, PanCancer Atlas)
Bladder Urothelial Carcinoma (TCGA, PanCancer Atlas)
Brain Lower Grade Glioma (TCGA, PanCancer Atlas)
Breast Invasive Carcinoma (TCGA, PanCancer Atlas)
Cervical Squamous Cell Carcinoma (TCGA, PanCancer Atlas)
Cholangiocarcinoma (TCGA, PanCancer Atlas)
Colorectal Adenocarcinoma (TCGA, PanCancer Atlas)
Diffuse Large B-Cell Lymphoma (TCGA, PanCancer Atlas)
Esophageal Adenocarcinoma (TCGA, PanCancer Atlas)
Glioblastoma Multiforme (TCGA, PanCancer Atlas)
Head and Neck Squamous Cell Carcinoma (TCGA, PanCancer Atlas)
Kidney Chromophobe (TCGA, PanCancer Atlas)
Kidney Renal Clear Cell Carcinoma (TCGA, PanCancer Atlas)
Kidney Renal Papillary Cell Carcinoma (TCGA, PanCancer Atlas)
Liver Hepatocellular Carcinoma (TCGA, PanCancer Atlas)
Lung Adenocarcinoma (TCGA, PanCancer Atlas)
Lung Squamous Cell Carcinoma (TCGA, PanCancer Atlas)
Mesothelioma (TCGA, PanCancer Atlas)
Ovarian Serous Cystadenocarcinoma (TCGA, PanCancer Atlas)
Pancreatic Adenocarcinoma (TCGA, PanCancer Atlas)
Pheochromocytoma and Paraganglioma (TCGA, PanCancer Atlas)
Prostate Adenocarcinoma (TCGA, PanCancer Atlas)
Sarcoma (TCGA, PanCancer Atlas)
Skin Cutaneous Melanoma (TCGA, PanCancer Atlas)
Stomach Adenocarcinoma (TCGA, PanCancer Atlas)
Testicular Germ Cell Tumors (TCGA, PanCancer Atlas)
Thymoma (TCGA, PanCancer Atlas)
Thyroid Carcinoma (TCGA, PanCancer Atlas)
Uterine Carcinosarcoma (TCGA, PanCancer Atlas)
Uterine Corpus Endometrial Carcinoma (TCGA, PanCancer Atlas)
Uveal Melanoma (TCGA, PanCancer Atlas)
Study of origin

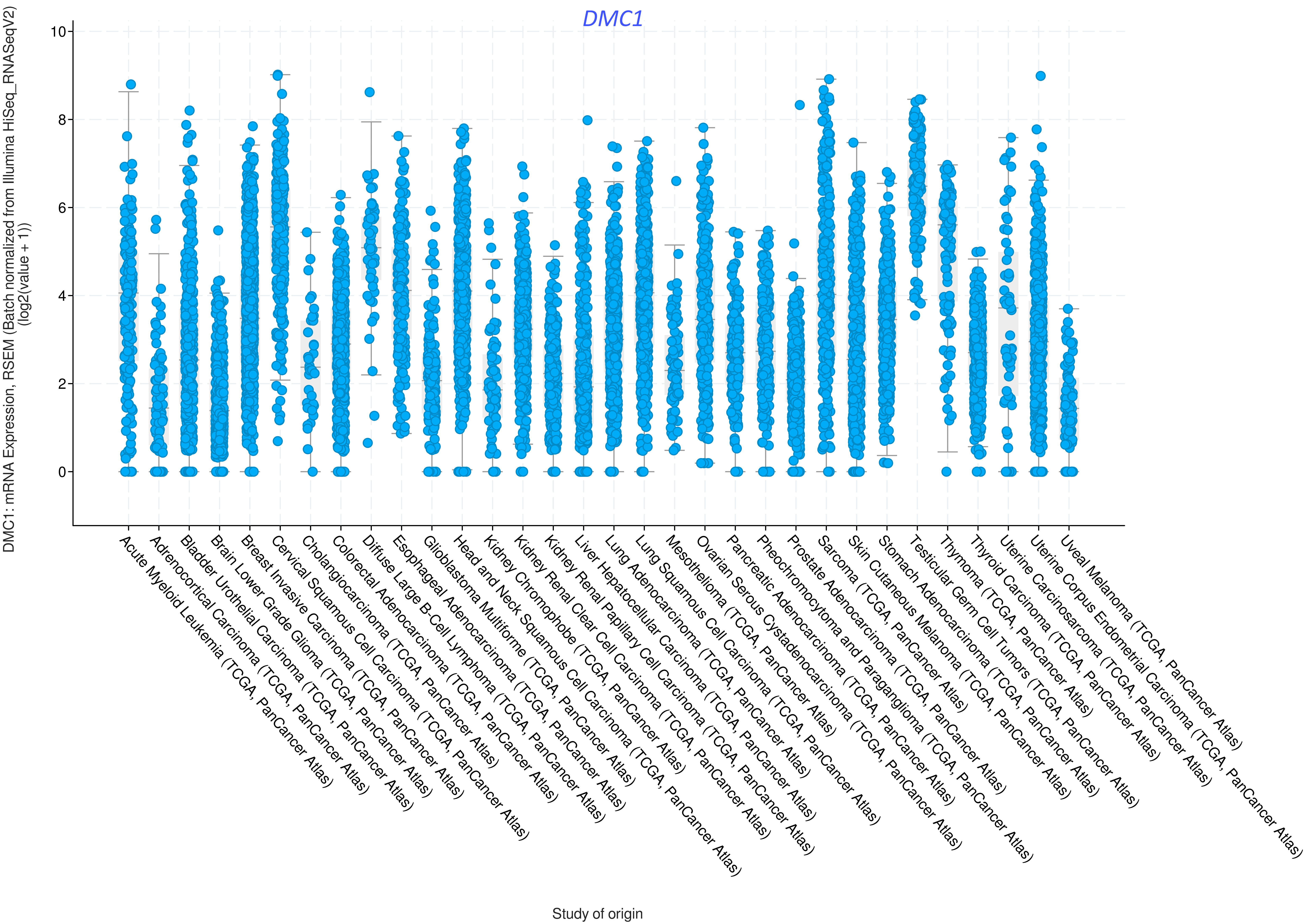

DMC1
DMC1: mRNA Expression, RSEM (Batch normalized from Illumina HiSeq_RNASeqV2) (log2(value + 1))
10
8
6
4
2
0
Study of origin
Acute Myeloid Leukemia (TCGA, PanCancer Atlas)
Adrenocortical Carcinoma (TCGA, PanCancer Atlas)
Bladder Urothelial Carcinoma (TCGA, PanCancer Atlas)
Brain Lower Grade Glioma (TCGA, PanCancer Atlas)
Breast Invasive Carcinoma (TCGA, PanCancer Atlas)
Cervical Squamous Cell Carcinoma (TCGA, PanCancer Atlas)
Cholangiocarcinoma (TCGA, PanCancer Atlas)
Colorectal Adenocarcinoma (TCGA, PanCancer Atlas)
Diffuse Large B-Cell Lymphoma (TCGA, PanCancer Atlas)
Esophageal Adenocarcinoma (TCGA, PanCancer Atlas)
Glioblastoma Multiforme (TCGA, PanCancer Atlas)
Head and Neck Squamous Cell Carcinoma (TCGA, PanCancer Atlas)
Kidney Chromophobe (TCGA, PanCancer Atlas)
Kidney Renal Clear Cell Carcinoma (TCGA, PanCancer Atlas)
Kidney Renal Papillary Cell Carcinoma (TCGA, PanCancer Atlas)
Liver Hepatocellular Carcinoma (TCGA, PanCancer Atlas)
Lung Adenocarcinoma (TCGA, PanCancer Atlas)
Lung Squamous Cell Carcinoma (TCGA, PanCancer Atlas)
Mesothelioma (TCGA, PanCancer Atlas)
Ovarian Serous Cystadenocarcinoma (TCGA, PanCancer Atlas)
Pancreatic Adenocarcinoma (TCGA, PanCancer Atlas)
Pheochromocytoma and Paraganglioma (TCGA, PanCancer Atlas)
Prostate Adenocarcinoma (TCGA, PanCancer Atlas)
Sarcoma (TCGA, PanCancer Atlas)
Skin Cutaneous Melanoma (TCGA, PanCancer Atlas)
Stomach Adenocarcinoma (TCGA, PanCancer Atlas)
Testicular Germ Cell Tumors (TCGA, PanCancer Atlas)
Thymoma (TCGA, PanCancer Atlas)
Thyroid Carcinoma (TCGA, PanCancer Atlas)
Uterine Carcinosarcoma (TCGA, PanCancer Atlas)
Uterine Corpus Endometrial Carcinoma (TCGA, PanCancer Atlas)
Uveal Melanoma (TCGA, PanCancer Atlas)

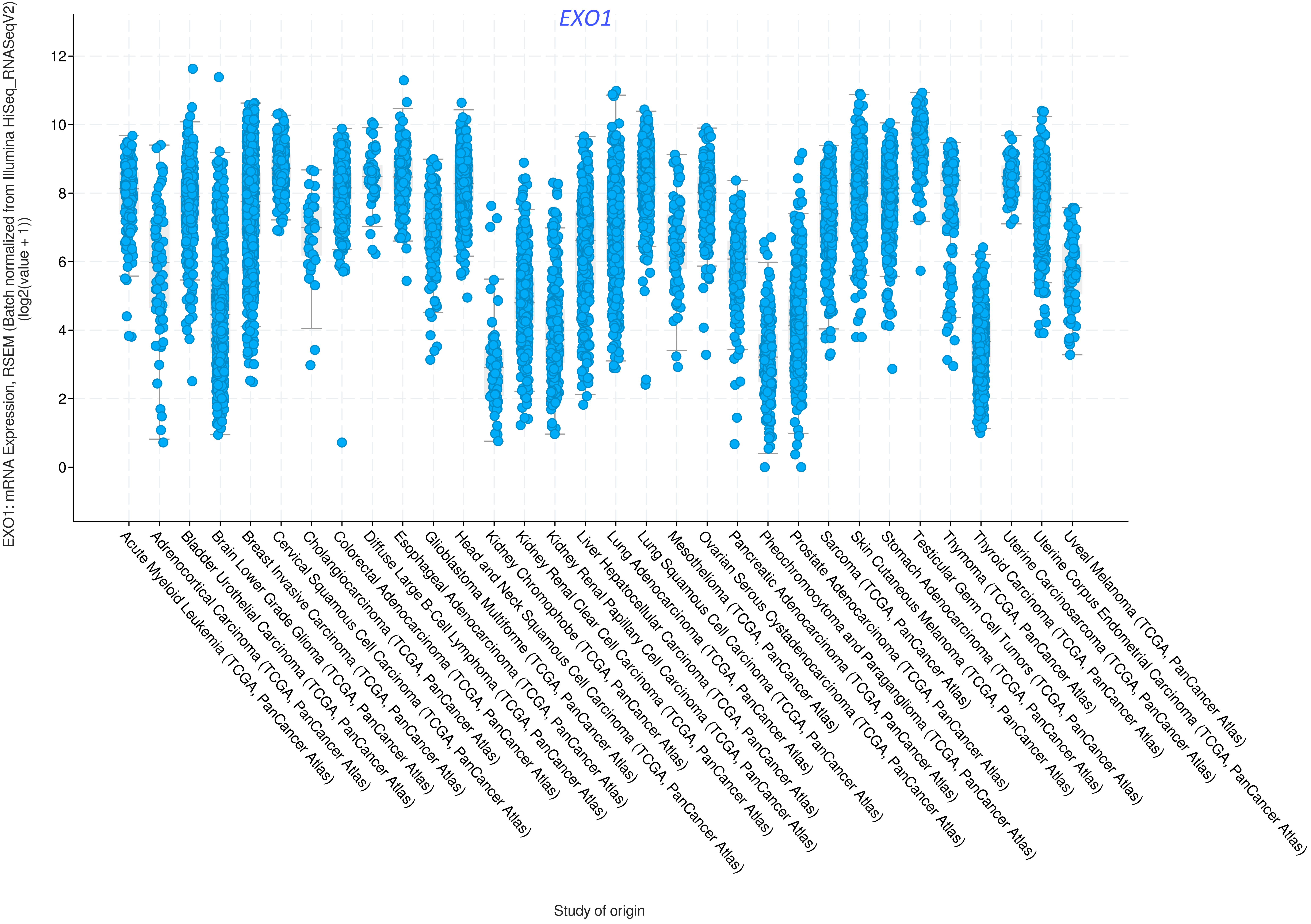

EXO1
EXO1: mRNA Expression, RSEM (Batch normalized from Illumina HiSeq_RNASeqV2) (log2(value + 1))
12
10
8
6
4
2
0
Acute Myeloid Leukemia (TCGA, PanCancer Atlas)
Adrenocortical Carcinoma (TCGA, PanCancer Atlas)
Bladder Urothelial Carcinoma (TCGA, PanCancer Atlas)
Brain Lower Grade Glioma (TCGA, PanCancer Atlas)
Breast Invasive Carcinoma (TCGA, PanCancer Atlas)
Cervical Squamous Cell Carcinoma (TCGA, PanCancer Atlas)
Cholangiocarcinoma (TCGA, PanCancer Atlas)
Colorectal Adenocarcinoma (TCGA, PanCancer Atlas)
Diffuse Large B-Cell Lymphoma (TCGA, PanCancer Atlas)
Esophageal Adenocarcinoma (TCGA, PanCancer Atlas)
Glioblastoma Multiforme (TCGA, PanCancer Atlas)
Head and Neck Squamous Cell Carcinoma (TCGA, PanCancer Atlas)
Kidney Chromophobe (TCGA, PanCancer Atlas)
Kidney Renal Clear Cell Carcinoma (TCGA, PanCancer Atlas)
Kidney Renal Papillary Cell Carcinoma (TCGA, PanCancer Atlas)
Liver Hepatocellular Carcinoma (TCGA, PanCancer Atlas)
Lung Adenocarcinoma (TCGA, PanCancer Atlas)
Lung Squamous Cell Carcinoma (TCGA, PanCancer Atlas)
Mesothelioma (TCGA, PanCancer Atlas)
Ovarian Serous Cystadenocarcinoma (TCGA, PanCancer Atlas)
Pancreatic Adenocarcinoma (TCGA, PanCancer Atlas)
Pheochromocytoma and Paraganglioma (TCGA, PanCancer Atlas)
Prostate Adenocarcinoma (TCGA, PanCancer Atlas)
Sarcoma (TCGA, PanCancer Atlas)
Skin Cutaneous Melanoma (TCGA, PanCancer Atlas)
Stomach Adenocarcinoma (TCGA, PanCancer Atlas)
Testicular Germ Cell Tumors (TCGA, PanCancer Atlas)
Thymoma (TCGA, PanCancer Atlas)
Thyroid Carcinoma (TCGA, PanCancer Atlas)
Uterine Carcinosarcoma (TCGA, PanCancer Atlas)
Uterine Corpus Endometrial Carcinoma (TCGA, PanCancer Atlas)
Uveal Melanoma (TCGA, PanCancer Atlas)
Study of origin

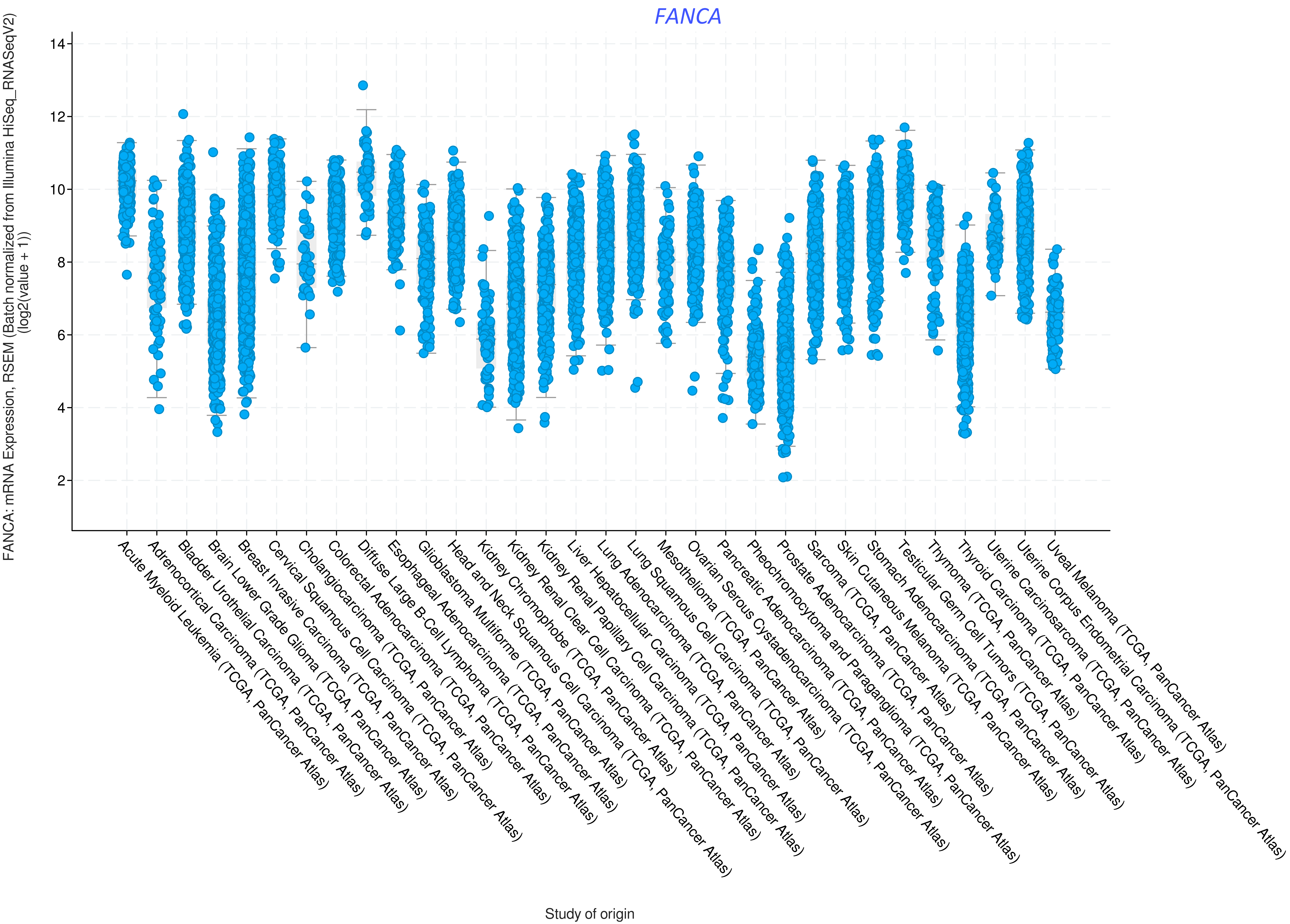

FANCA
FANCA: mRNA Expression, RSEM (Batch normalized from Illumina HiSeq_RNASeqV2) (log2(value + 1))
2
4
6
8
10
12
14
Study of origin
Acute Myeloid Leukemia (TCGA, PanCancer Atlas)
Adrenocortical Carcinoma (TCGA, PanCancer Atlas)
Bladder Urothelial Carcinoma (TCGA, PanCancer Atlas)
Brain Lower Grade Glioma (TCGA, PanCancer Atlas)
Breast Invasive Carcinoma (TCGA, PanCancer Atlas)
Cervical Squamous Cell Carcinoma (TCGA, PanCancer Atlas)
Cholangiocarcinoma (TCGA, PanCancer Atlas)
Colorectal Adenocarcinoma (TCGA, PanCancer Atlas)
Diffuse Large B-Cell Lymphoma (TCGA, PanCancer Atlas)
Esophageal Adenocarcinoma (TCGA, PanCancer Atlas)
Glioblastoma Multiforme (TCGA, PanCancer Atlas)
Head and Neck Squamous Cell Carcinoma (TCGA, PanCancer Atlas)
Kidney Chromophobe (TCGA, PanCancer Atlas)
Kidney Renal Clear Cell Carcinoma (TCGA, PanCancer Atlas)
Kidney Renal Papillary Cell Carcinoma (TCGA, PanCancer Atlas)
Liver Hepatocellular Carcinoma (TCGA, PanCancer Atlas)
Lung Adenocarcinoma (TCGA, PanCancer Atlas)
Lung Squamous Cell Carcinoma (TCGA, PanCancer Atlas)
Mesothelioma (TCGA, PanCancer Atlas)
Ovarian Serous Cystadenocarcinoma (TCGA, PanCancer Atlas)
Pancreatic Adenocarcinoma (TCGA, PanCancer Atlas)
Pheochromocytoma and Paraganglioma (TCGA, PanCancer Atlas)
Prostate Adenocarcinoma (TCGA, PanCancer Atlas)
Sarcoma (TCGA, PanCancer Atlas)
Skin Cutaneous Melanoma (TCGA, PanCancer Atlas)
Stomach Adenocarcinoma (TCGA, PanCancer Atlas)
Testicular Germ Cell Tumors (TCGA, PanCancer Atlas)
Thymoma (TCGA, PanCancer Atlas)
Thyroid Carcinoma (TCGA, PanCancer Atlas)
Uterine Carcinosarcoma (TCGA, PanCancer Atlas)
Uterine Corpus Endometrial Carcinoma (TCGA, PanCancer Atlas)
Uveal Melanoma (TCGA, PanCancer Atlas)

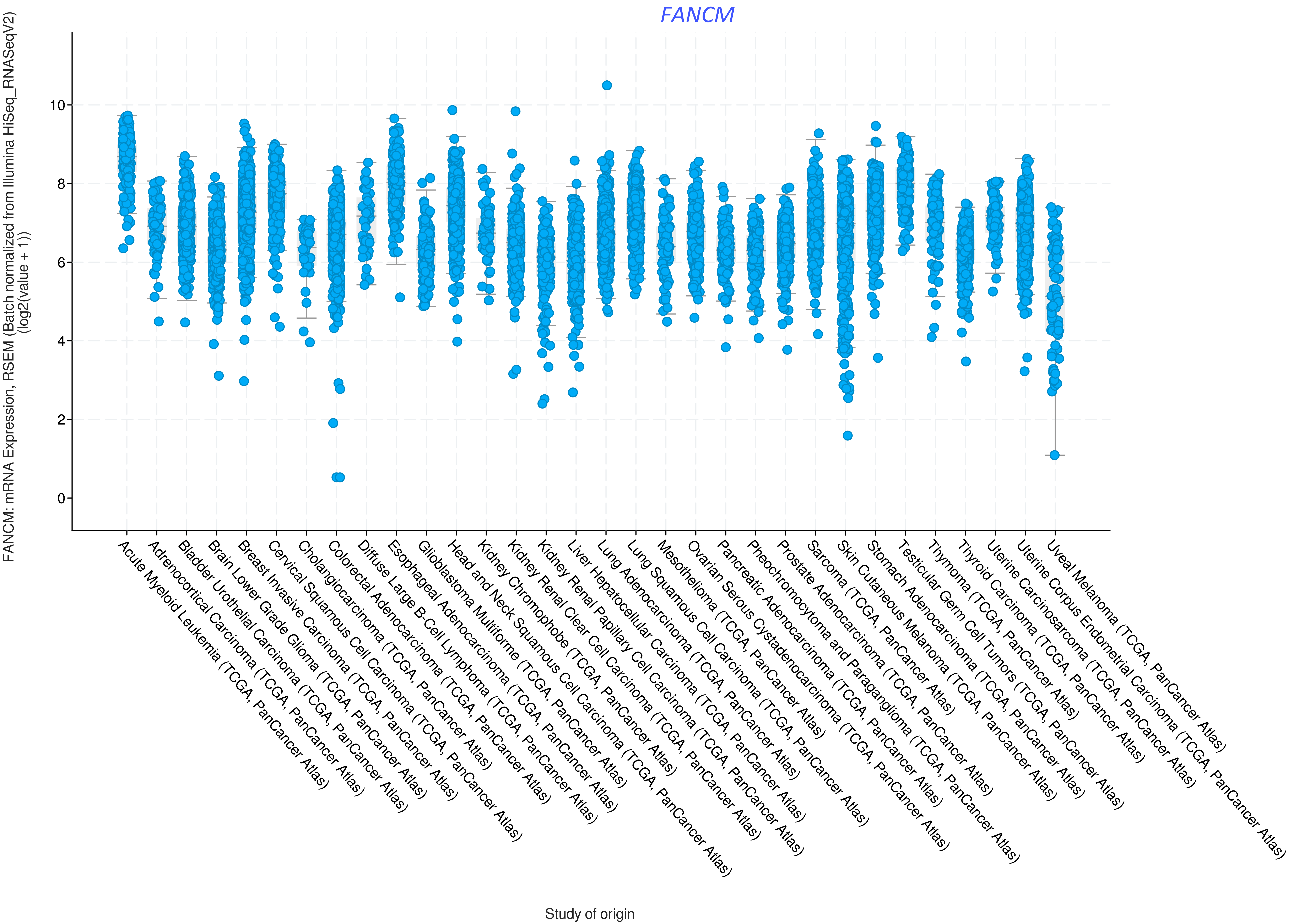

FANCM
FANCM: mRNA Expression, RSEM (Batch normalized from Illumina HiSeq_RNASeqV2) (log2(value + 1))
0
2
4
6
8
10
Study of origin
Acute Myeloid Leukemia (TCGA, PanCancer Atlas)
Adrenocortical Carcinoma (TCGA, PanCancer Atlas)
Bladder Urothelial Carcinoma (TCGA, PanCancer Atlas)
Brain Lower Grade Glioma (TCGA, PanCancer Atlas)
Breast Invasive Carcinoma (TCGA, PanCancer Atlas)
Cervical Squamous Cell Carcinoma (TCGA, PanCancer Atlas)
Cholangiocarcinoma (TCGA, PanCancer Atlas)
Colorectal Adenocarcinoma (TCGA, PanCancer Atlas)
Diffuse Large B-Cell Lymphoma (TCGA, PanCancer Atlas)
Esophageal Adenocarcinoma (TCGA, PanCancer Atlas)
Glioblastoma Multiforme (TCGA, PanCancer Atlas)
Head and Neck Squamous Cell Carcinoma (TCGA, PanCancer Atlas)
Kidney Chromophobe (TCGA, PanCancer Atlas)
Kidney Renal Clear Cell Carcinoma (TCGA, PanCancer Atlas)
Kidney Renal Papillary Cell Carcinoma (TCGA, PanCancer Atlas)
Liver Hepatocellular Carcinoma (TCGA, PanCancer Atlas)
Lung Adenocarcinoma (TCGA, PanCancer Atlas)
Lung Squamous Cell Carcinoma (TCGA, PanCancer Atlas)
Mesothelioma (TCGA, PanCancer Atlas)
Ovarian Serous Cystadenocarcinoma (TCGA, PanCancer Atlas)
Pancreatic Adenocarcinoma (TCGA, PanCancer Atlas)
Pheochromocytoma and Paraganglioma (TCGA, PanCancer Atlas)
Prostate Adenocarcinoma (TCGA, PanCancer Atlas)
Sarcoma (TCGA, PanCancer Atlas)
Skin Cutaneous Melanoma (TCGA, PanCancer Atlas)
Stomach Adenocarcinoma (TCGA, PanCancer Atlas)
Testicular Germ Cell Tumors (TCGA, PanCancer Atlas)
Thymoma (TCGA, PanCancer Atlas)
Thyroid Carcinoma (TCGA, PanCancer Atlas)
Uterine Carcinosarcoma (TCGA, PanCancer Atlas)
Uterine Corpus Endometrial Carcinoma (TCGA, PanCancer Atlas)
Uveal Melanoma (TCGA, PanCancer Atlas)

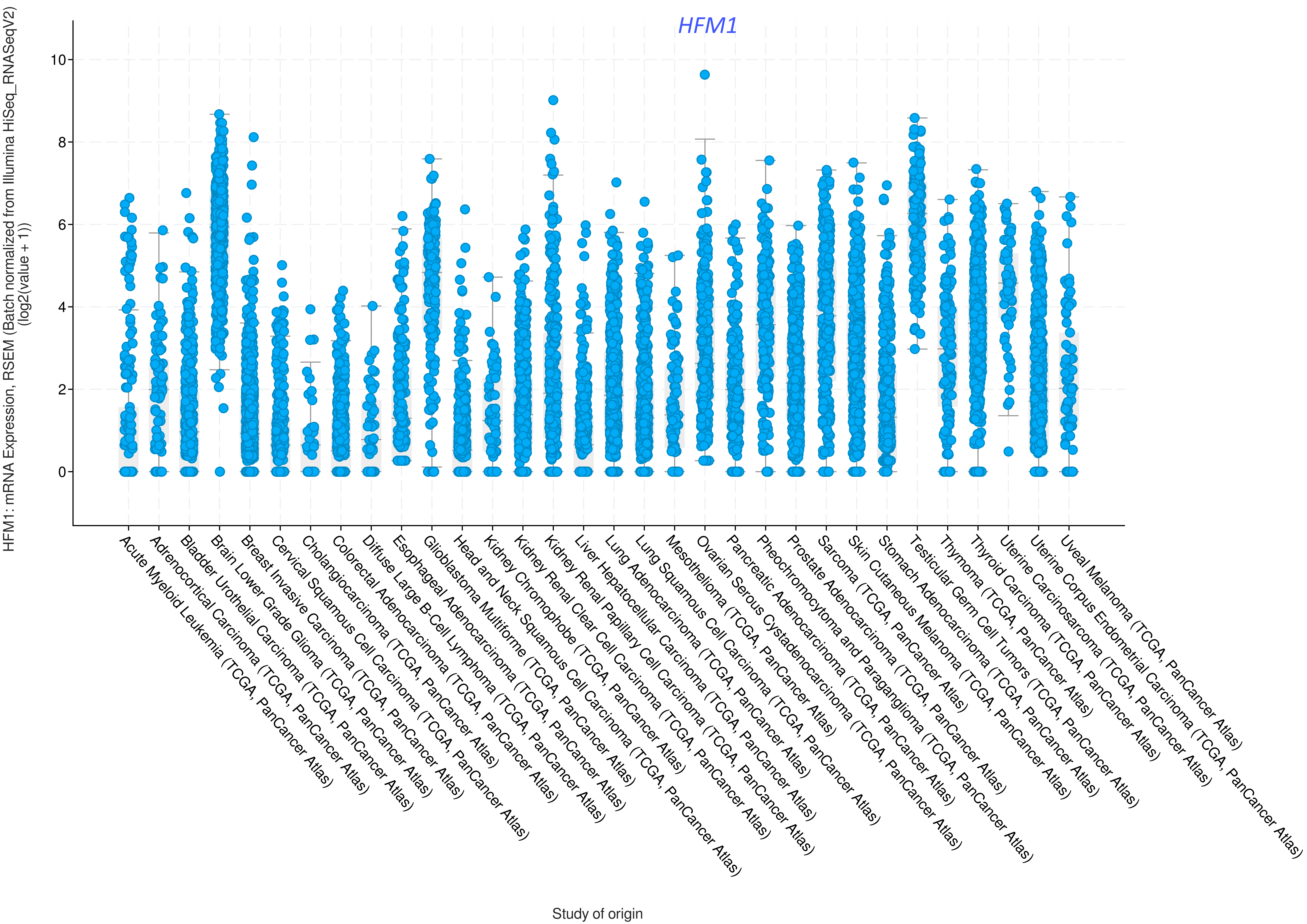

HFM1
HFM1: mRNA Expression, RSEM (Batch normalized from Illumina HiSeq_RNASeqV2) (log2(value + 1))
0
2
4
6
8
10
Acute Myeloid Leukemia (TCGA, PanCancer Atlas)
Adrenocortical Carcinoma (TCGA, PanCancer Atlas)
Bladder Urothelial Carcinoma (TCGA, PanCancer Atlas)
Brain Lower Grade Glioma (TCGA, PanCancer Atlas)
Breast Invasive Carcinoma (TCGA, PanCancer Atlas)
Cervical Squamous Cell Carcinoma (TCGA, PanCancer Atlas)
Cholangiocarcinoma (TCGA, PanCancer Atlas)
Colorectal Adenocarcinoma (TCGA, PanCancer Atlas)
Diffuse Large B-Cell Lymphoma (TCGA, PanCancer Atlas)
Esophageal Adenocarcinoma (TCGA, PanCancer Atlas)
Glioblastoma Multiforme (TCGA, PanCancer Atlas)
Head and Neck Squamous Cell Carcinoma (TCGA, PanCancer Atlas)
Kidney Chromophobe (TCGA, PanCancer Atlas)
Kidney Renal Clear Cell Carcinoma (TCGA, PanCancer Atlas)
Kidney Renal Papillary Cell Carcinoma (TCGA, PanCancer Atlas)
Liver Hepatocellular Carcinoma (TCGA, PanCancer Atlas)
Lung Adenocarcinoma (TCGA, PanCancer Atlas)
Lung Squamous Cell Carcinoma (TCGA, PanCancer Atlas)
Mesothelioma (TCGA, PanCancer Atlas)
Ovarian Serous Cystadenocarcinoma (TCGA, PanCancer Atlas)
Pancreatic Adenocarcinoma (TCGA, PanCancer Atlas)
Pheochromocytoma and Paraganglioma (TCGA, PanCancer Atlas)
Prostate Adenocarcinoma (TCGA, PanCancer Atlas)
Sarcoma (TCGA, PanCancer Atlas)
Skin Cutaneous Melanoma (TCGA, PanCancer Atlas)
Stomach Adenocarcinoma (TCGA, PanCancer Atlas)
Testicular Germ Cell Tumors (TCGA, PanCancer Atlas)
Thymoma (TCGA, PanCancer Atlas)
Thyroid Carcinoma (TCGA, PanCancer Atlas)
Uterine Carcinosarcoma (TCGA, PanCancer Atlas)
Uterine Corpus Endometrial Carcinoma (TCGA, PanCancer Atlas)
Uveal Melanoma (TCGA, PanCancer Atlas)
Study of origin

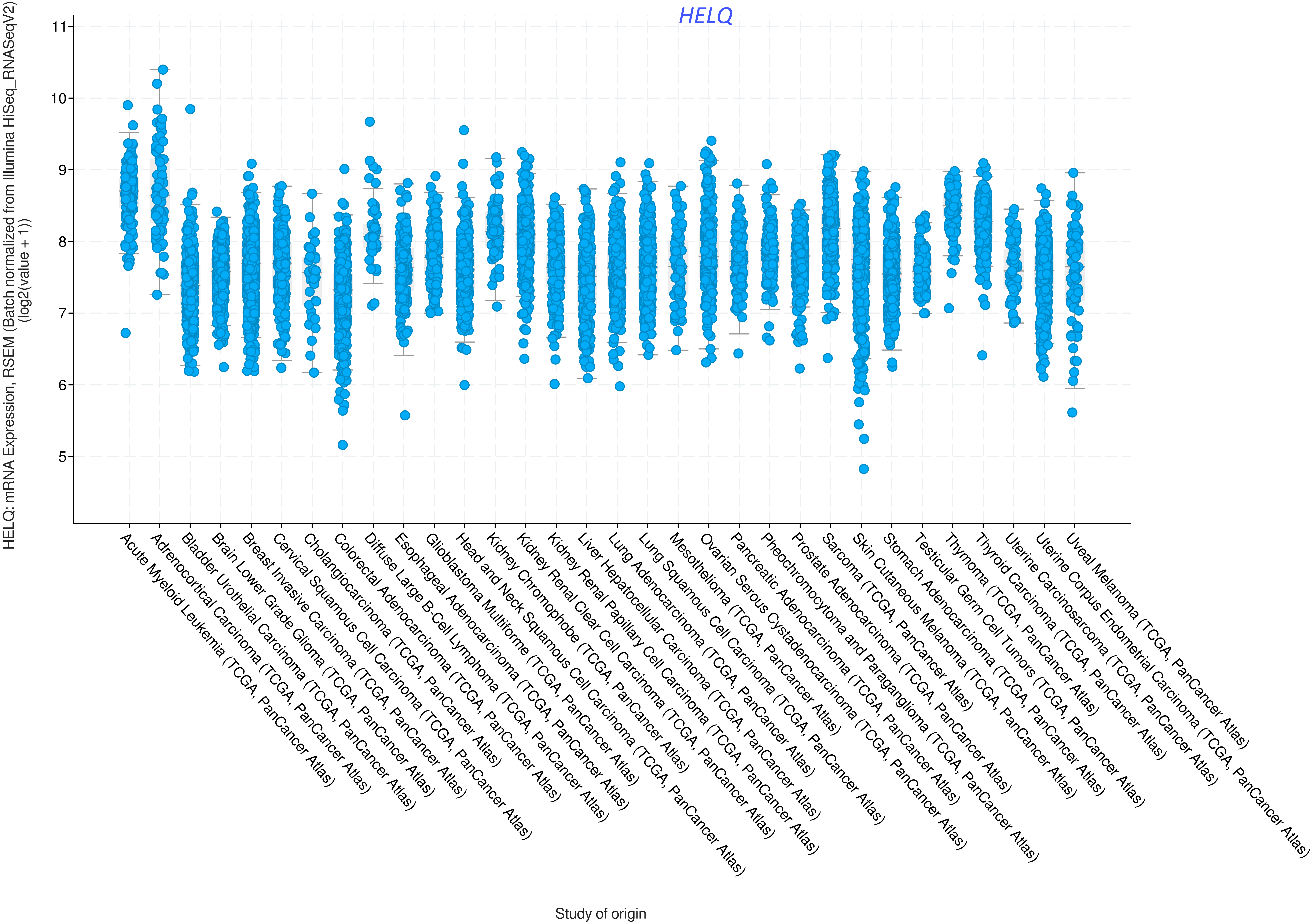
HELQ
HELQ: mRNA Expression, RSEM (Batch normalized from Illumina HiSeq_RNASeqV2) (log2(value + 1))
Study of origin
11
10
9
8
7
6
5
Acute Myeloid Leukemia (TCGA, PanCancer Atlas)
Adrenocortical Carcinoma (TCGA, PanCancer Atlas)
Bladder Urothelial Carcinoma (TCGA, PanCancer Atlas)
Brain Lower Grade Glioma (TCGA, PanCancer Atlas)
Breast Invasive Carcinoma (TCGA, PanCancer Atlas)
Cervical Squamous Cell Carcinoma (TCGA, PanCancer Atlas)
Cholangiocarcinoma (TCGA, PanCancer Atlas)
Colorectal Adenocarcinoma (TCGA, PanCancer Atlas)
Diffuse Large B-Cell Lymphoma (TCGA, PanCancer Atlas)
Esophageal Adenocarcinoma (TCGA, PanCancer Atlas)
Glioblastoma Multiforme (TCGA, PanCancer Atlas)
Head and Neck Squamous Cell Carcinoma (TCGA, PanCancer Atlas)
Kidney Chromophobe (TCGA, PanCancer Atlas)
Kidney Renal Clear Cell Carcinoma (TCGA, PanCancer Atlas)
Kidney Renal Papillary Cell Carcinoma (TCGA, PanCancer Atlas)
Liver Hepatocellular Carcinoma (TCGA, PanCancer Atlas)
Lung Adenocarcinoma (TCGA, PanCancer Atlas)
Lung Squamous Cell Carcinoma (TCGA, PanCancer Atlas)
Mesothelioma (TCGA, PanCancer Atlas)
Ovarian Serous Cystadenocarcinoma (TCGA, PanCancer Atlas)
Pancreatic Adenocarcinoma (TCGA, PanCancer Atlas)
Pheochromocytoma and Paraganglioma (TCGA, PanCancer Atlas)
Prostate Adenocarcinoma (TCGA, PanCancer Atlas)
Sarcoma (TCGA, PanCancer Atlas)
Skin Cutaneous Melanoma (TCGA, PanCancer Atlas)
Stomach Adenocarcinoma (TCGA, PanCancer Atlas)
Testicular Germ Cell Tumors (TCGA, PanCancer Atlas)
Thymoma (TCGA, PanCancer Atlas)
Thyroid Carcinoma (TCGA, PanCancer Atlas)
Uterine Carcinosarcoma (TCGA, PanCancer Atlas)
Uterine Corpus Endometrial Carcinoma (TCGA, PanCancer Atlas)
Uveal Melanoma (TCGA, PanCancer Atlas)

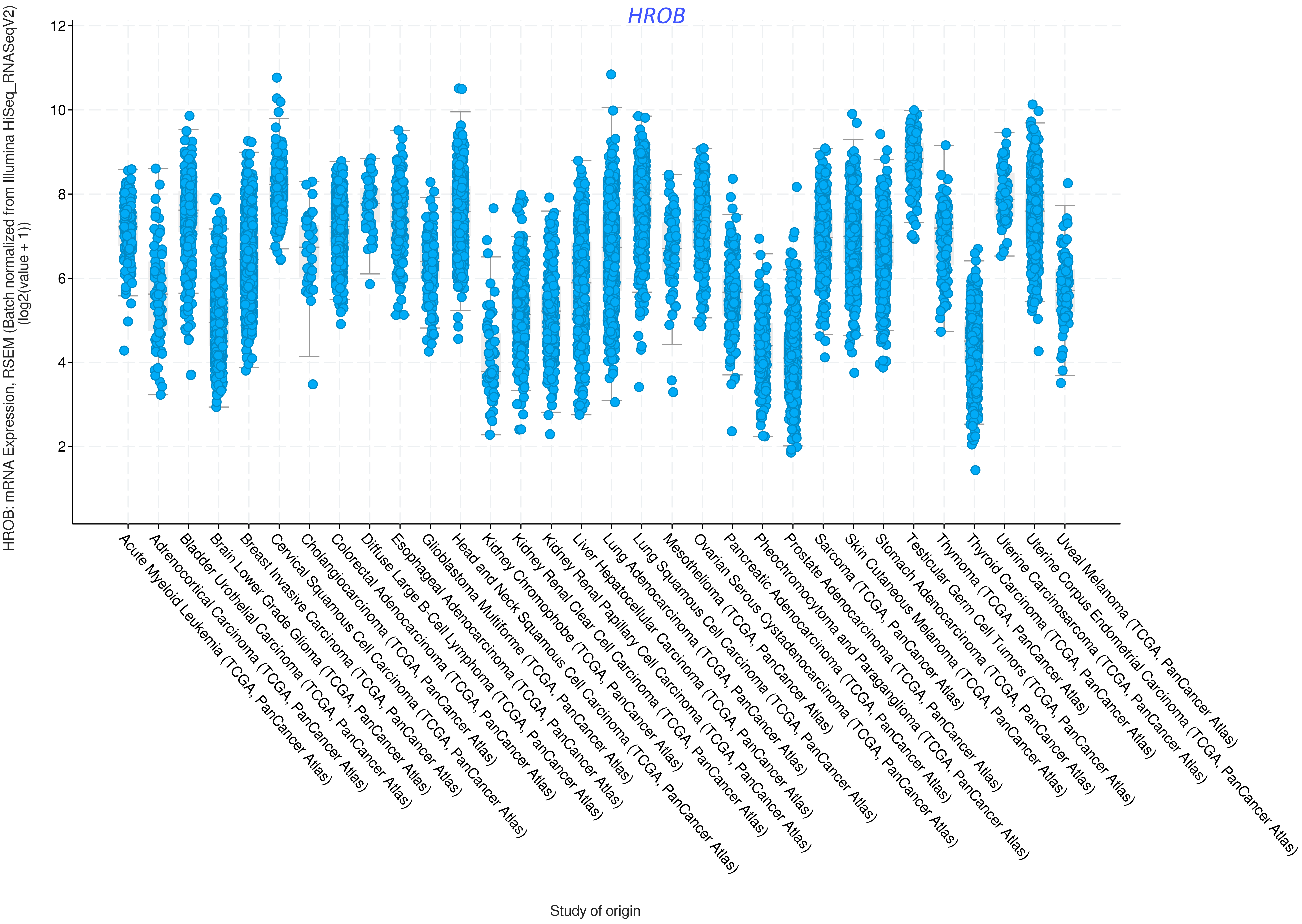

HROB
HROB: mRNA Expression, RSEM (Batch normalized from Illumina HiSeq_RNASeqV2) (log2(value + 1))
12
10
8
6
4
2
Study of origin
Acute Myeloid Leukemia (TCGA, PanCancer Atlas)
Adrenocortical Carcinoma (TCGA, PanCancer Atlas)
Bladder Urothelial Carcinoma (TCGA, PanCancer Atlas)
Brain Lower Grade Glioma (TCGA, PanCancer Atlas)
Breast Invasive Carcinoma (TCGA, PanCancer Atlas)
Cervical Squamous Cell Carcinoma (TCGA, PanCancer Atlas)
Cholangiocarcinoma (TCGA, PanCancer Atlas)
Colorectal Adenocarcinoma (TCGA, PanCancer Atlas)
Diffuse Large B-Cell Lymphoma (TCGA, PanCancer Atlas)
Esophageal Adenocarcinoma (TCGA, PanCancer Atlas)
Glioblastoma Multiforme (TCGA, PanCancer Atlas)
Head and Neck Squamous Cell Carcinoma (TCGA, PanCancer Atlas)
Kidney Chromophobe (TCGA, PanCancer Atlas)
Kidney Renal Clear Cell Carcinoma (TCGA, PanCancer Atlas)
Kidney Renal Papillary Cell Carcinoma (TCGA, PanCancer Atlas)
Liver Hepatocellular Carcinoma (TCGA, PanCancer Atlas)
Lung Adenocarcinoma (TCGA, PanCancer Atlas)
Lung Squamous Cell Carcinoma (TCGA, PanCancer Atlas)
Mesothelioma (TCGA, PanCancer Atlas)
Ovarian Serous Cystadenocarcinoma (TCGA, PanCancer Atlas)
Pancreatic Adenocarcinoma (TCGA, PanCancer Atlas)
Pheochromocytoma and Paraganglioma (TCGA, PanCancer Atlas)
Prostate Adenocarcinoma (TCGA, PanCancer Atlas)
Sarcoma (TCGA, PanCancer Atlas)
Skin Cutaneous Melanoma (TCGA, PanCancer Atlas)
Stomach Adenocarcinoma (TCGA, PanCancer Atlas)
Testicular Germ Cell Tumors (TCGA, PanCancer Atlas)
Thymoma (TCGA, PanCancer Atlas)
Thyroid Carcinoma (TCGA, PanCancer Atlas)
Uterine Carcinosarcoma (TCGA, PanCancer Atlas)
Uterine Corpus Endometrial Carcinoma (TCGA, PanCancer Atlas)
Uveal Melanoma (TCGA, PanCancer Atlas)

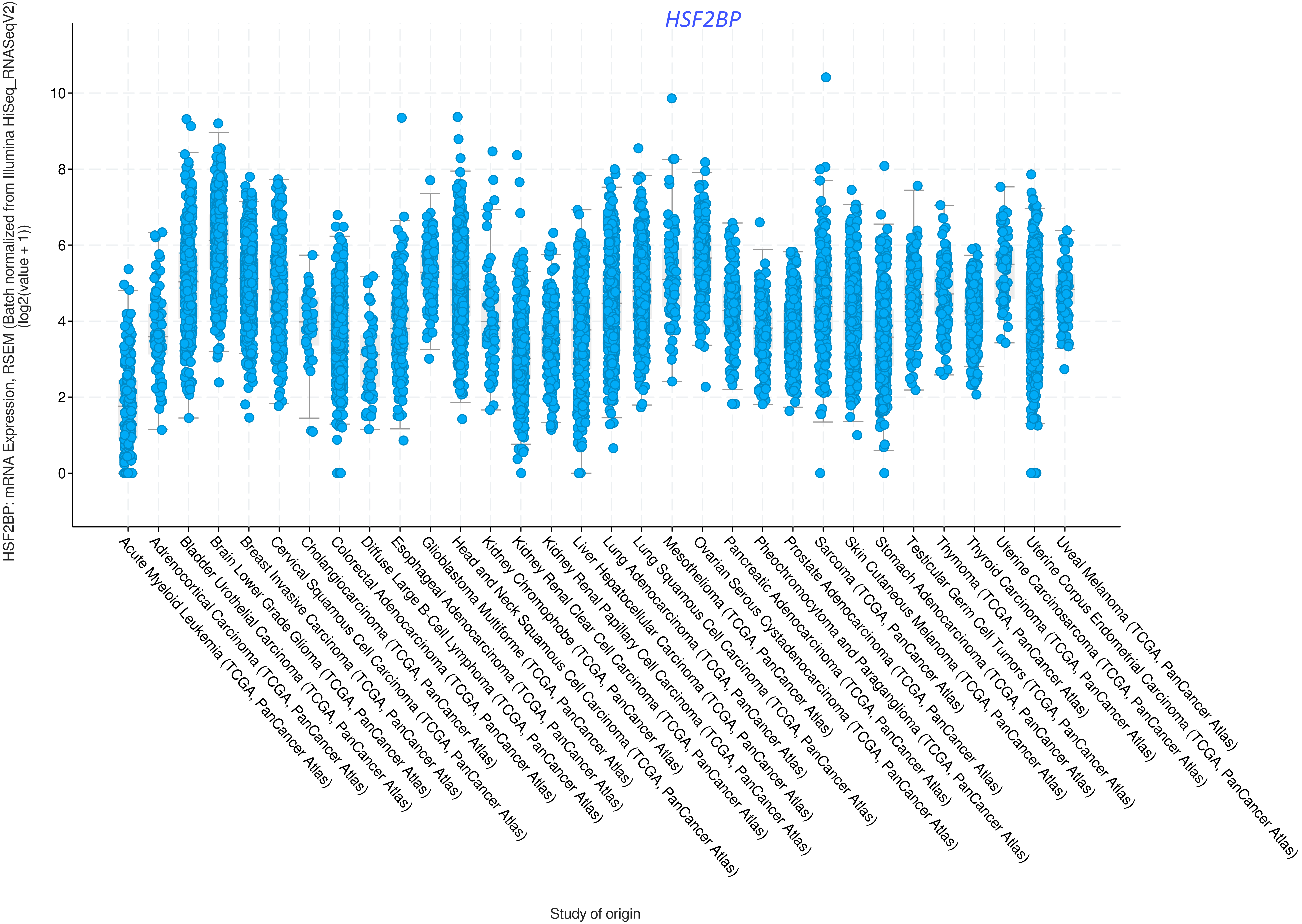

HSF2BP
HSF2BP: mRNA Expression, RSEM (Batch normalized from Illumina HiSeq_RNASeqV2) (log2(value + 1))
Study of origin
0
2
4
6
8
10
Acute Myeloid Leukemia (TCGA, PanCancer Atlas)
Adrenocortical Carcinoma (TCGA, PanCancer Atlas)
Bladder Urothelial Carcinoma (TCGA, PanCancer Atlas)
Brain Lower Grade Glioma (TCGA, PanCancer Atlas)
Breast Invasive Carcinoma (TCGA, PanCancer Atlas)
Cervical Squamous Cell Carcinoma (TCGA, PanCancer Atlas)
Cholangiocarcinoma (TCGA, PanCancer Atlas)
Colorectal Adenocarcinoma (TCGA, PanCancer Atlas)
Diffuse Large B-Cell Lymphoma (TCGA, PanCancer Atlas)
Esophageal Adenocarcinoma (TCGA, PanCancer Atlas)
Glioblastoma Multiforme (TCGA, PanCancer Atlas)
Head and Neck Squamous Cell Carcinoma (TCGA, PanCancer Atlas)
Kidney Chromophobe (TCGA, PanCancer Atlas)
Kidney Renal Clear Cell Carcinoma (TCGA, PanCancer Atlas)
Kidney Renal Papillary Cell Carcinoma (TCGA, PanCancer Atlas)
Liver Hepatocellular Carcinoma (TCGA, PanCancer Atlas)
Lung Adenocarcinoma (TCGA, PanCancer Atlas)
Lung Squamous Cell Carcinoma (TCGA, PanCancer Atlas)
Mesothelioma (TCGA, PanCancer Atlas)
Ovarian Serous Cystadenocarcinoma (TCGA, PanCancer Atlas)
Pancreatic Adenocarcinoma (TCGA, PanCancer Atlas)
Pheochromocytoma and Paraganglioma (TCGA, PanCancer Atlas)
Prostate Adenocarcinoma (TCGA, PanCancer Atlas)
Sarcoma (TCGA, PanCancer Atlas)
Skin Cutaneous Melanoma (TCGA, PanCancer Atlas)
Stomach Adenocarcinoma (TCGA, PanCancer Atlas)
Testicular Germ Cell Tumors (TCGA, PanCancer Atlas)
Thymoma (TCGA, PanCancer Atlas)
Thyroid Carcinoma (TCGA, PanCancer Atlas)
Uterine Carcinosarcoma (TCGA, PanCancer Atlas)
Uterine Corpus Endometrial Carcinoma (TCGA, PanCancer Atlas)
Uveal Melanoma (TCGA, PanCancer Atlas)

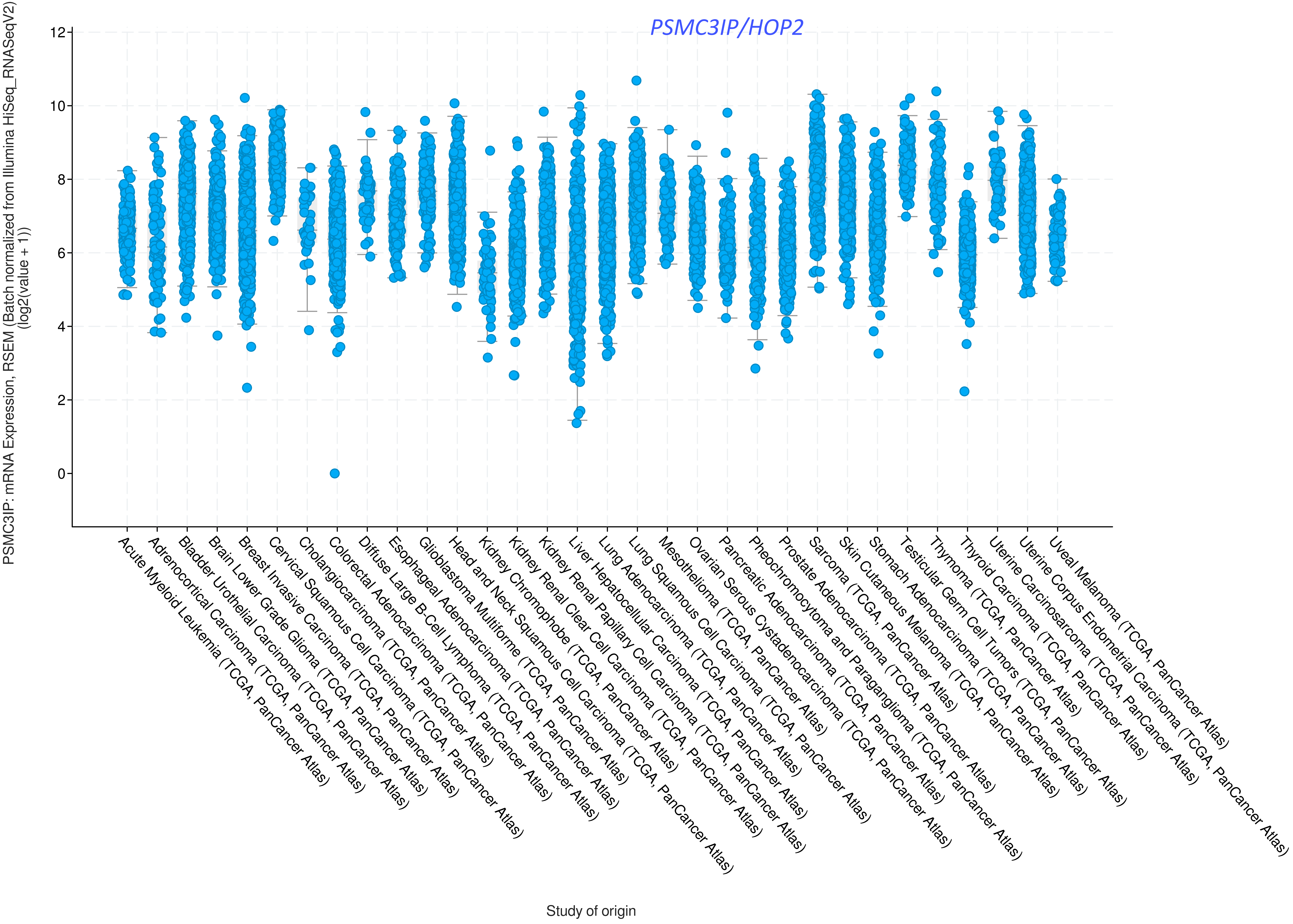

PSMC3IP/HOP2
PSMC3IP: mRNA Expression, RSEM (Batch normalized from Illumina HiSeq_RNASeqV2) (log2(value + 1))
12
10
8
6
4
2
0
Study of origin
Acute Myeloid Leukemia (TCGA, PanCancer Atlas)
Adrenocortical Carcinoma (TCGA, PanCancer Atlas)
Bladder Urothelial Carcinoma (TCGA, PanCancer Atlas)
Brain Lower Grade Glioma (TCGA, PanCancer Atlas)
Breast Invasive Carcinoma (TCGA, PanCancer Atlas)
Cervical Squamous Cell Carcinoma (TCGA, PanCancer Atlas)
Cholangiocarcinoma (TCGA, PanCancer Atlas)
Colorectal Adenocarcinoma (TCGA, PanCancer Atlas)
Diffuse Large B-Cell Lymphoma (TCGA, PanCancer Atlas)
Esophageal Adenocarcinoma (TCGA, PanCancer Atlas)
Glioblastoma Multiforme (TCGA, PanCancer Atlas)
Head and Neck Squamous Cell Carcinoma (TCGA, PanCancer Atlas)
Kidney Chromophobe (TCGA, PanCancer Atlas)
Kidney Renal Clear Cell Carcinoma (TCGA, PanCancer Atlas)
Kidney Renal Papillary Cell Carcinoma (TCGA, PanCancer Atlas)
Liver Hepatocellular Carcinoma (TCGA, PanCancer Atlas)
Lung Adenocarcinoma (TCGA, PanCancer Atlas)
Lung Squamous Cell Carcinoma (TCGA, PanCancer Atlas)
Mesothelioma (TCGA, PanCancer Atlas)
Ovarian Serous Cystadenocarcinoma (TCGA, PanCancer Atlas)
Pancreatic Adenocarcinoma (TCGA, PanCancer Atlas)
Pheochromocytoma and Paraganglioma (TCGA, PanCancer Atlas)
Prostate Adenocarcinoma (TCGA, PanCancer Atlas)
Sarcoma (TCGA, PanCancer Atlas)
Skin Cutaneous Melanoma (TCGA, PanCancer Atlas)
Stomach Adenocarcinoma (TCGA, PanCancer Atlas)
Testicular Germ Cell Tumors (TCGA, PanCancer Atlas)
Thymoma (TCGA, PanCancer Atlas)
Thyroid Carcinoma (TCGA, PanCancer Atlas)
Uterine Carcinosarcoma (TCGA, PanCancer Atlas)
Uterine Corpus Endometrial Carcinoma (TCGA, PanCancer Atlas)
Uveal Melanoma (TCGA, PanCancer Atlas)

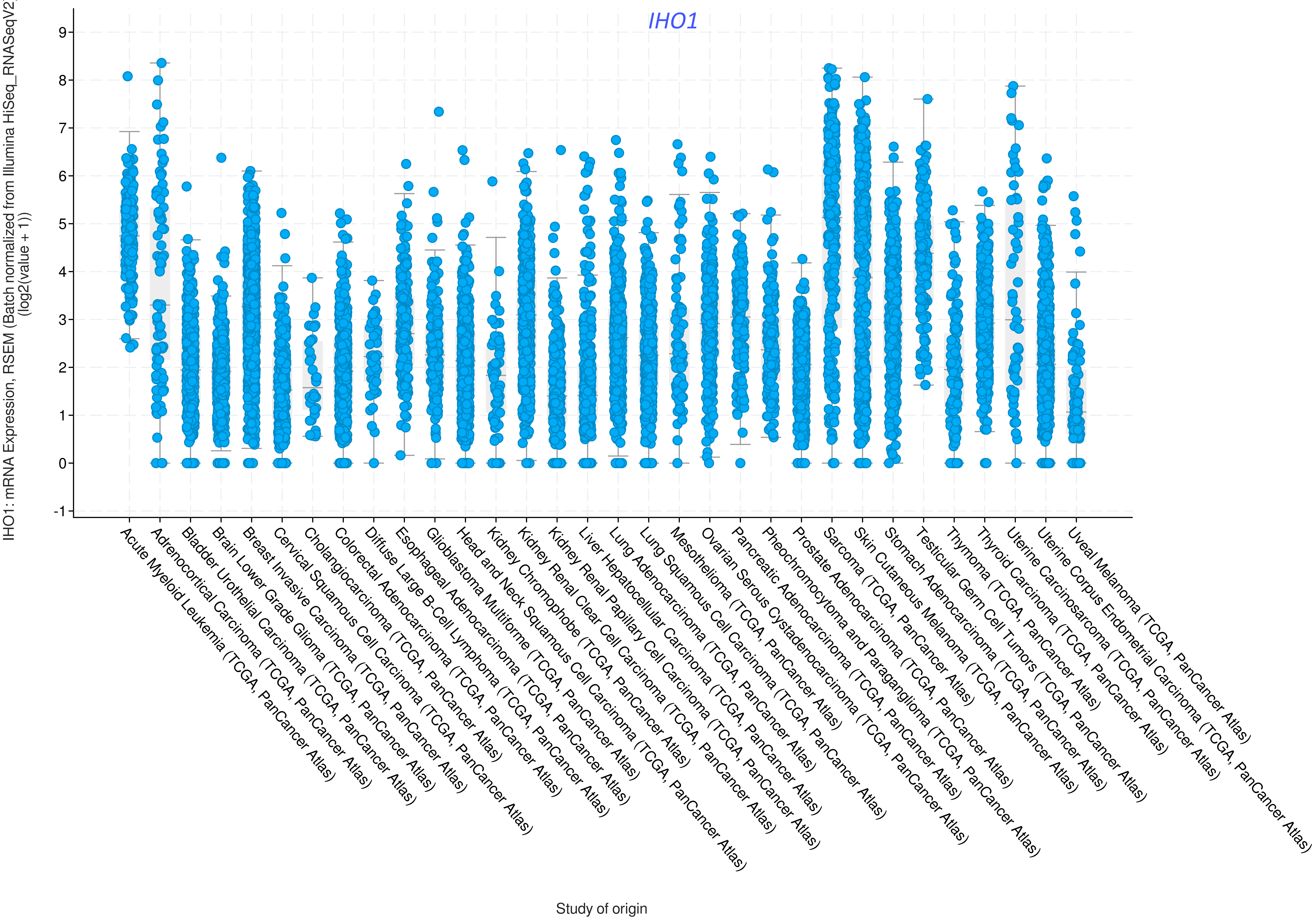

IHO1
IHO1: mRNA Expression, RSEM (Batch normalized from Illumina HiSeq_RNASeqV2) (log2(value + 1))
9
8
7
6
5
4
3
2
1
0
-1
Acute Myeloid Leukemia (TCGA, PanCancer Atlas)
Adrenocortical Carcinoma (TCGA, PanCancer Atlas)
Bladder Urothelial Carcinoma (TCGA, PanCancer Atlas)
Brain Lower Grade Glioma (TCGA, PanCancer Atlas)
Breast Invasive Carcinoma (TCGA, PanCancer Atlas)
Cervical Squamous Cell Carcinoma (TCGA, PanCancer Atlas)
Cholangiocarcinoma (TCGA, PanCancer Atlas)
Colorectal Adenocarcinoma (TCGA, PanCancer Atlas)
Diffuse Large B-Cell Lymphoma (TCGA, PanCancer Atlas)
Esophageal Adenocarcinoma (TCGA, PanCancer Atlas)
Glioblastoma Multiforme (TCGA, PanCancer Atlas)
Head and Neck Squamous Cell Carcinoma (TCGA, PanCancer Atlas)
Kidney Chromophobe (TCGA, PanCancer Atlas)
Kidney Renal Clear Cell Carcinoma (TCGA, PanCancer Atlas)
Kidney Renal Papillary Cell Carcinoma (TCGA, PanCancer Atlas)
Liver Hepatocellular Carcinoma (TCGA, PanCancer Atlas)
Lung Adenocarcinoma (TCGA, PanCancer Atlas)
Lung Squamous Cell Carcinoma (TCGA, PanCancer Atlas)
Mesothelioma (TCGA, PanCancer Atlas)
Ovarian Serous Cystadenocarcinoma (TCGA, PanCancer Atlas)
Pancreatic Adenocarcinoma (TCGA, PanCancer Atlas)
Pheochromocytoma and Paraganglioma (TCGA, PanCancer Atlas)
Prostate Adenocarcinoma (TCGA, PanCancer Atlas)
Sarcoma (TCGA, PanCancer Atlas)
Skin Cutaneous Melanoma (TCGA, PanCancer Atlas)
Stomach Adenocarcinoma (TCGA, PanCancer Atlas)
Testicular Germ Cell Tumors (TCGA, PanCancer Atlas)
Thymoma (TCGA, PanCancer Atlas)
Thyroid Carcinoma (TCGA, PanCancer Atlas)
Uterine Carcinosarcoma (TCGA, PanCancer Atlas)
Uterine Corpus Endometrial Carcinoma (TCGA, PanCancer Atlas)
Uveal Melanoma (TCGA, PanCancer Atlas)
Study of origin

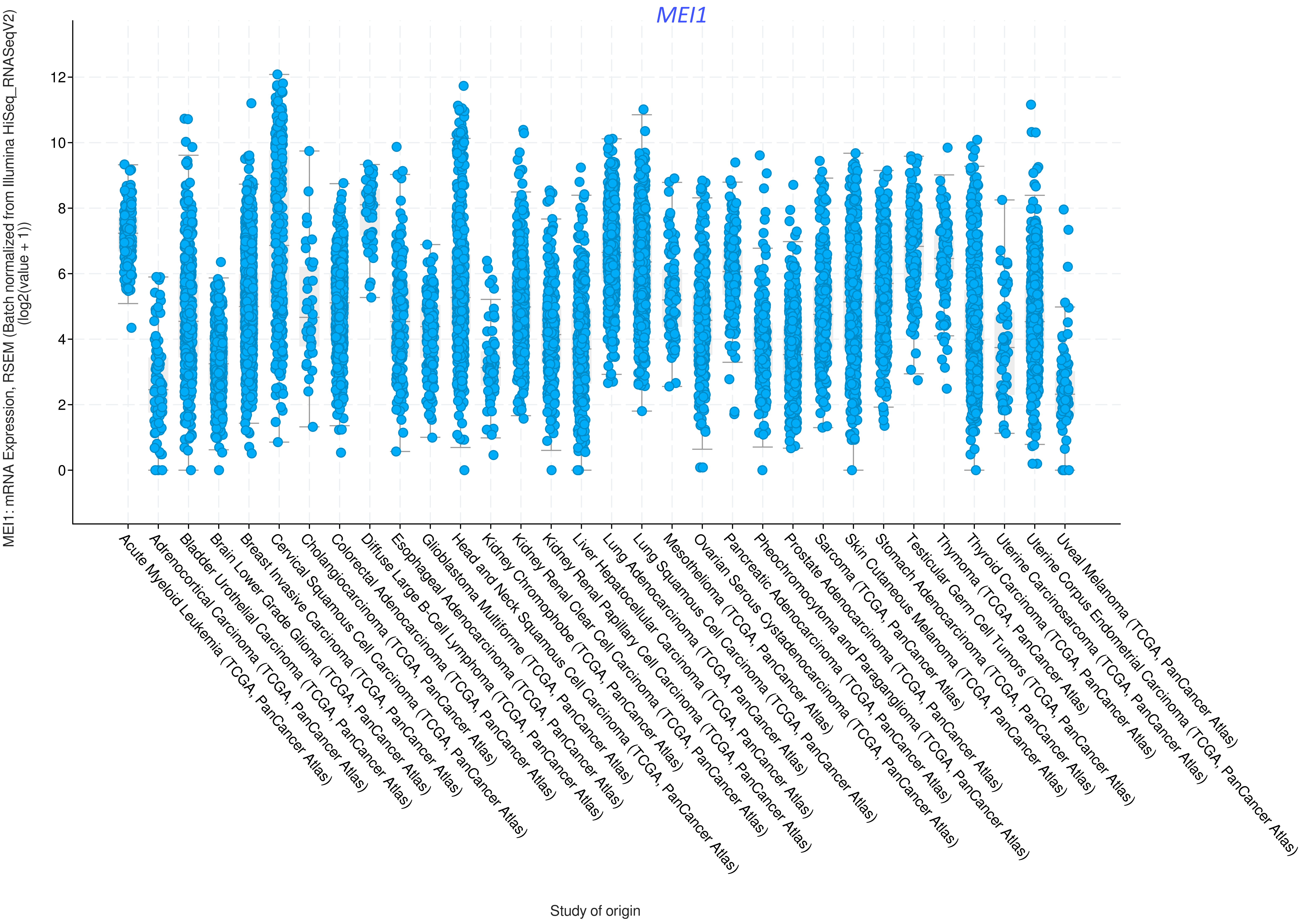

MEI1
MEI1: mRNA Expression, RSEM (Batch normalized from Illumina HiSeq_RNASeqV2) (log2(value + 1))
12
10
8
6
4
2
0
Acute Myeloid Leukemia (TCGA, PanCancer Atlas)
Adrenocortical Carcinoma (TCGA, PanCancer Atlas)
Bladder Urothelial Carcinoma (TCGA, PanCancer Atlas)
Brain Lower Grade Glioma (TCGA, PanCancer Atlas)
Breast Invasive Carcinoma (TCGA, PanCancer Atlas)
Cervical Squamous Cell Carcinoma (TCGA, PanCancer Atlas)
Cholangiocarcinoma (TCGA, PanCancer Atlas)
Colorectal Adenocarcinoma (TCGA, PanCancer Atlas)
Diffuse Large B-Cell Lymphoma (TCGA, PanCancer Atlas)
Esophageal Adenocarcinoma (TCGA, PanCancer Atlas)
Glioblastoma Multiforme (TCGA, PanCancer Atlas)
Head and Neck Squamous Cell Carcinoma (TCGA, PanCancer Atlas)
Kidney Chromophobe (TCGA, PanCancer Atlas)
Kidney Renal Clear Cell Carcinoma (TCGA, PanCancer Atlas)
Kidney Renal Papillary Cell Carcinoma (TCGA, PanCancer Atlas)
Liver Hepatocellular Carcinoma (TCGA, PanCancer Atlas)
Lung Adenocarcinoma (TCGA, PanCancer Atlas)
Lung Squamous Cell Carcinoma (TCGA, PanCancer Atlas)
Mesothelioma (TCGA, PanCancer Atlas)
Ovarian Serous Cystadenocarcinoma (TCGA, PanCancer Atlas)
Pancreatic Adenocarcinoma (TCGA, PanCancer Atlas)
Pheochromocytoma and Paraganglioma (TCGA, PanCancer Atlas)
Prostate Adenocarcinoma (TCGA, PanCancer Atlas)
Sarcoma (TCGA, PanCancer Atlas)
Skin Cutaneous Melanoma (TCGA, PanCancer Atlas)
Stomach Adenocarcinoma (TCGA, PanCancer Atlas)
Testicular Germ Cell Tumors (TCGA, PanCancer Atlas)
Thymoma (TCGA, PanCancer Atlas)
Thyroid Carcinoma (TCGA, PanCancer Atlas)
Uterine Carcinosarcoma (TCGA, PanCancer Atlas)
Uterine Corpus Endometrial Carcinoma (TCGA, PanCancer Atlas)
Uveal Melanoma (TCGA, PanCancer Atlas)
Study of origin

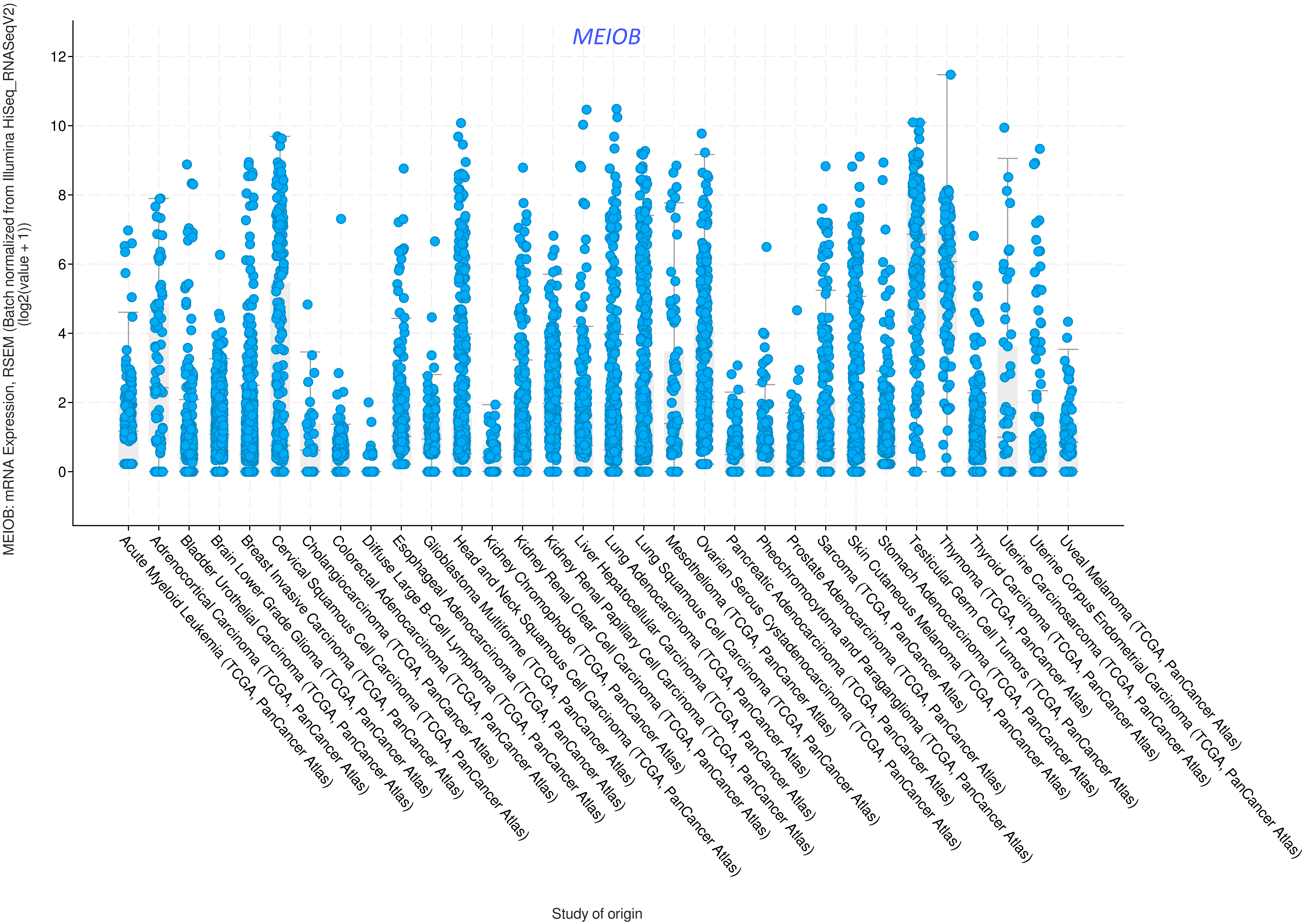
MEIOB
MEIOB: mRNA Expression, RSEM (Batch normalized from Illumina HiSeq_RNASeqV2) (log2(value + 1))
Study of origin
Acute Myeloid Leukemia (TCGA, PanCancer Atlas)
Adrenocortical Carcinoma (TCGA, PanCancer Atlas)
Bladder Urothelial Carcinoma (TCGA, PanCancer Atlas)
Brain Lower Grade Glioma (TCGA, PanCancer Atlas)
Breast Invasive Carcinoma (TCGA, PanCancer Atlas)
Cervical Squamous Cell Carcinoma (TCGA, PanCancer Atlas)
Cholangiocarcinoma (TCGA, PanCancer Atlas)
Colorectal Adenocarcinoma (TCGA, PanCancer Atlas)
Diffuse Large B-Cell Lymphoma (TCGA, PanCancer Atlas)
Esophageal Adenocarcinoma (TCGA, PanCancer Atlas)
Glioblastoma Multiforme (TCGA, PanCancer Atlas)
Head and Neck Squamous Cell Carcinoma (TCGA, PanCancer Atlas)
Kidney Chromophobe (TCGA, PanCancer Atlas)
Kidney Renal Clear Cell Carcinoma (TCGA, PanCancer Atlas)
Kidney Renal Papillary Cell Carcinoma (TCGA, PanCancer Atlas)
Liver Hepatocellular Carcinoma (TCGA, PanCancer Atlas)
Lung Adenocarcinoma (TCGA, PanCancer Atlas)
Lung Squamous Cell Carcinoma (TCGA, PanCancer Atlas)
Mesothelioma (TCGA, PanCancer Atlas)
Ovarian Serous Cystadenocarcinoma (TCGA, PanCancer Atlas)
Pancreatic Adenocarcinoma (TCGA, PanCancer Atlas)
Pheochromocytoma and Paraganglioma (TCGA, PanCancer Atlas)
Prostate Adenocarcinoma (TCGA, PanCancer Atlas)
Sarcoma (TCGA, PanCancer Atlas)
Skin Cutaneous Melanoma (TCGA, PanCancer Atlas)
Stomach Adenocarcinoma (TCGA, PanCancer Atlas)
Testicular Germ Cell Tumors (TCGA, PanCancer Atlas)
Thymoma (TCGA, PanCancer Atlas)
Thyroid Carcinoma (TCGA, PanCancer Atlas)
Uterine Carcinosarcoma (TCGA, PanCancer Atlas)
Uterine Corpus Endometrial Carcinoma (TCGA, PanCancer Atlas)
Uveal Melanoma (TCGA, PanCancer Atlas)
0
2
4
6
8
10
12

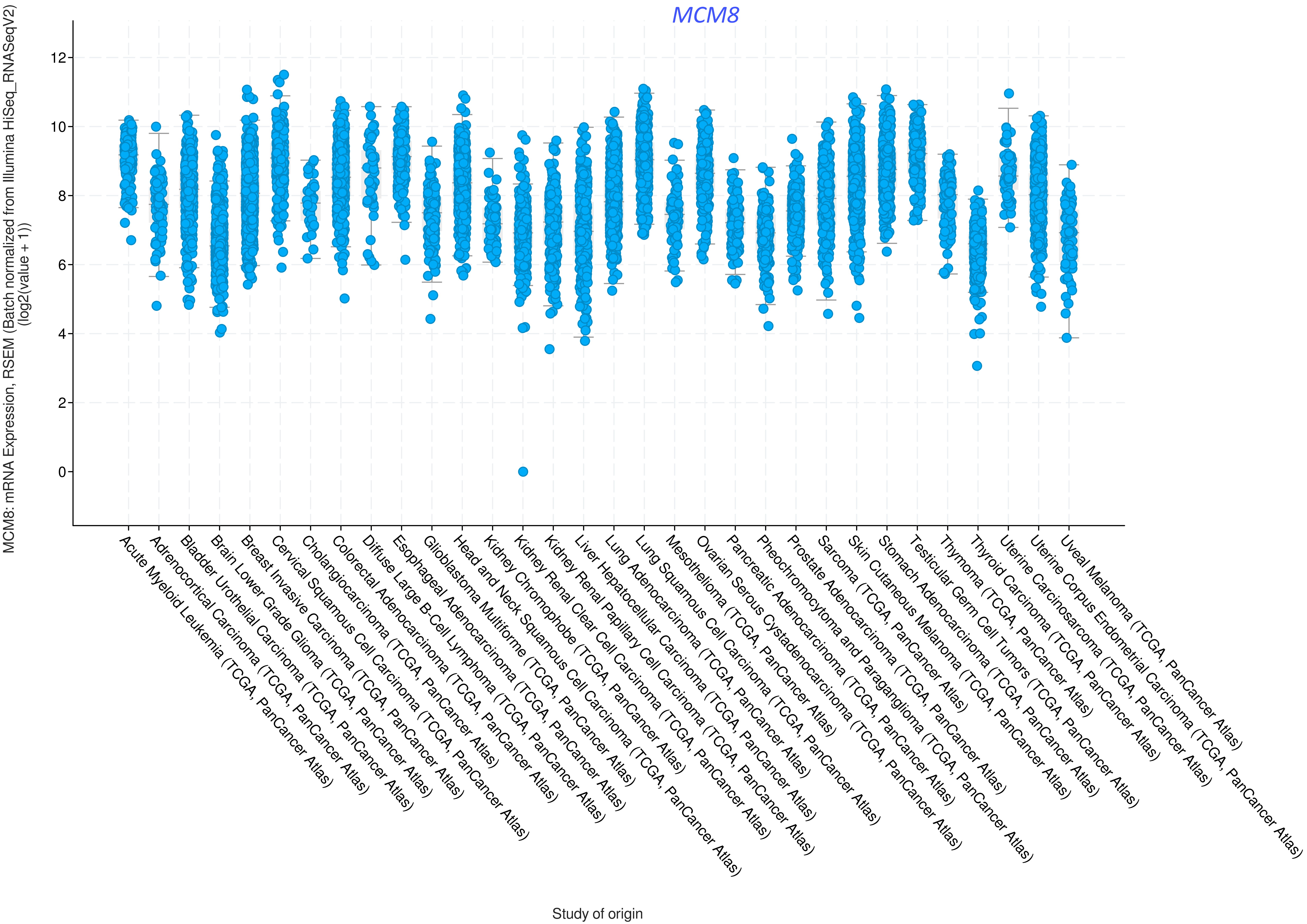

MCM8
MCM8: mRNA Expression, RSEM (Batch normalized from Illumina HiSeq_RNASeqV2) (log2(value + 1))
12
10
8
6
4
2
0
Acute Myeloid Leukemia (TCGA, PanCancer Atlas)
Adrenocortical Carcinoma (TCGA, PanCancer Atlas)
Bladder Urothelial Carcinoma (TCGA, PanCancer Atlas)
Brain Lower Grade Glioma (TCGA, PanCancer Atlas)
Breast Invasive Carcinoma (TCGA, PanCancer Atlas)
Cervical Squamous Cell Carcinoma (TCGA, PanCancer Atlas)
Cholangiocarcinoma (TCGA, PanCancer Atlas)
Colorectal Adenocarcinoma (TCGA, PanCancer Atlas)
Diffuse Large B-Cell Lymphoma (TCGA, PanCancer Atlas)
Esophageal Adenocarcinoma (TCGA, PanCancer Atlas)
Glioblastoma Multiforme (TCGA, PanCancer Atlas)
Head and Neck Squamous Cell Carcinoma (TCGA, PanCancer Atlas)
Kidney Chromophobe (TCGA, PanCancer Atlas)
Kidney Renal Clear Cell Carcinoma (TCGA, PanCancer Atlas)
Kidney Renal Papillary Cell Carcinoma (TCGA, PanCancer Atlas)
Liver Hepatocellular Carcinoma (TCGA, PanCancer Atlas)
Lung Adenocarcinoma (TCGA, PanCancer Atlas)
Lung Squamous Cell Carcinoma (TCGA, PanCancer Atlas)
Mesothelioma (TCGA, PanCancer Atlas)
Ovarian Serous Cystadenocarcinoma (TCGA, PanCancer Atlas)
Pancreatic Adenocarcinoma (TCGA, PanCancer Atlas)
Pheochromocytoma and Paraganglioma (TCGA, PanCancer Atlas)
Prostate Adenocarcinoma (TCGA, PanCancer Atlas)
Sarcoma (TCGA, PanCancer Atlas)
Skin Cutaneous Melanoma (TCGA, PanCancer Atlas)
Stomach Adenocarcinoma (TCGA, PanCancer Atlas)
Testicular Germ Cell Tumors (TCGA, PanCancer Atlas)
Thymoma (TCGA, PanCancer Atlas)
Thyroid Carcinoma (TCGA, PanCancer Atlas)
Uterine Carcinosarcoma (TCGA, PanCancer Atlas)
Uterine Corpus Endometrial Carcinoma (TCGA, PanCancer Atlas)
Uveal Melanoma (TCGA, PanCancer Atlas)
Study of origin

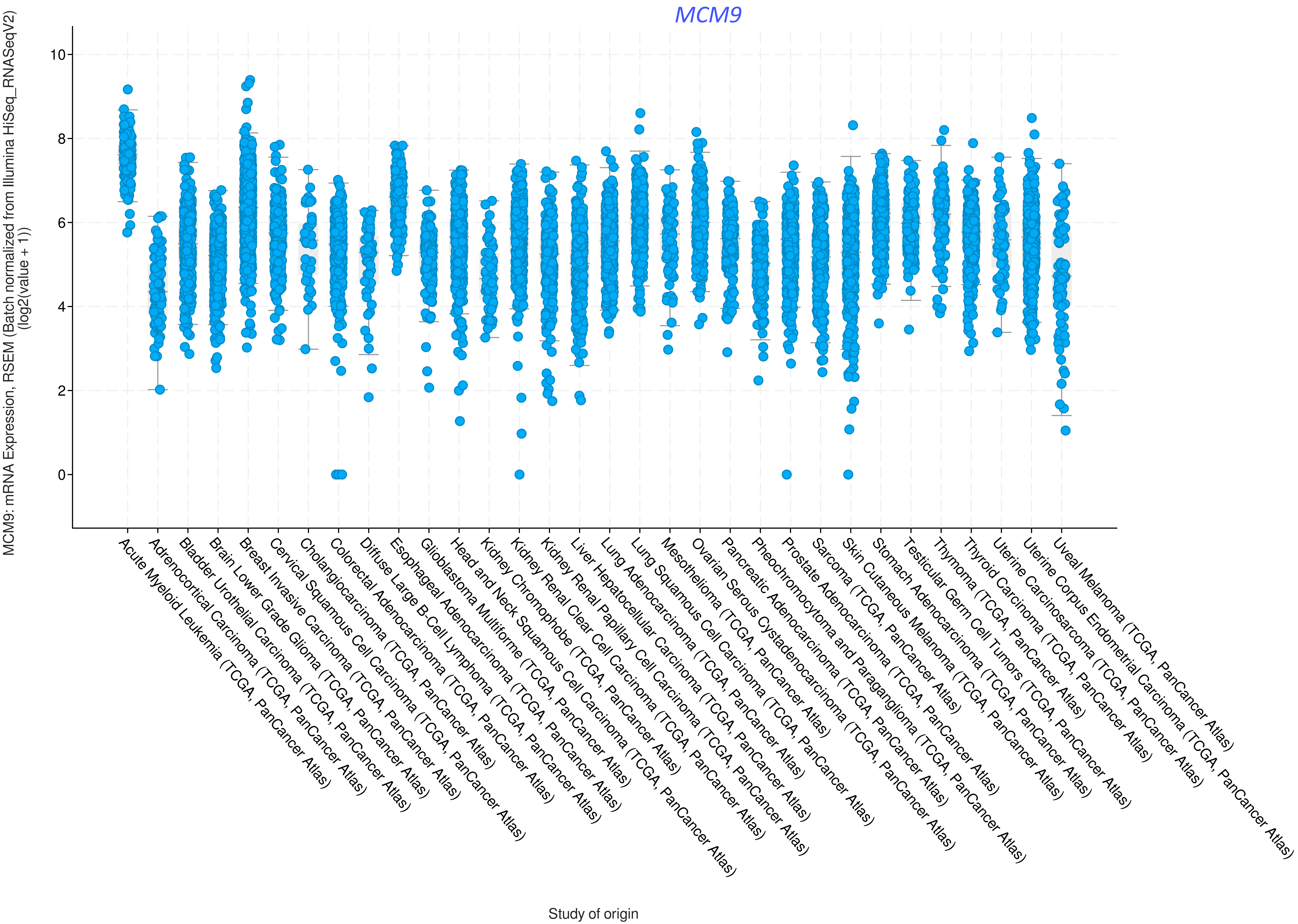

MCM9
MCM9: mRNA Expression, RSEM (Batch normalized from Illumina HiSeq_RNASeqV2) (log2(value + 1))
10
8
6
4
2
0
Study of origin
Acute Myeloid Leukemia (TCGA, PanCancer Atlas)
Adrenocortical Carcinoma (TCGA, PanCancer Atlas)
Bladder Urothelial Carcinoma (TCGA, PanCancer Atlas)
Brain Lower Grade Glioma (TCGA, PanCancer Atlas)
Breast Invasive Carcinoma (TCGA, PanCancer Atlas)
Cervical Squamous Cell Carcinoma (TCGA, PanCancer Atlas)
Cholangiocarcinoma (TCGA, PanCancer Atlas)
Colorectal Adenocarcinoma (TCGA, PanCancer Atlas)
Diffuse Large B-Cell Lymphoma (TCGA, PanCancer Atlas)
Esophageal Adenocarcinoma (TCGA, PanCancer Atlas)
Glioblastoma Multiforme (TCGA, PanCancer Atlas)
Head and Neck Squamous Cell Carcinoma (TCGA, PanCancer Atlas)
Kidney Chromophobe (TCGA, PanCancer Atlas)
Kidney Renal Clear Cell Carcinoma (TCGA, PanCancer Atlas)
Kidney Renal Papillary Cell Carcinoma (TCGA, PanCancer Atlas)
Liver Hepatocellular Carcinoma (TCGA, PanCancer Atlas)
Lung Adenocarcinoma (TCGA, PanCancer Atlas)
Lung Squamous Cell Carcinoma (TCGA, PanCancer Atlas)
Mesothelioma (TCGA, PanCancer Atlas)
Ovarian Serous Cystadenocarcinoma (TCGA, PanCancer Atlas)
Pancreatic Adenocarcinoma (TCGA, PanCancer Atlas)
Pheochromocytoma and Paraganglioma (TCGA, PanCancer Atlas)
Prostate Adenocarcinoma (TCGA, PanCancer Atlas)
Sarcoma (TCGA, PanCancer Atlas)
Skin Cutaneous Melanoma (TCGA, PanCancer Atlas)
Stomach Adenocarcinoma (TCGA, PanCancer Atlas)
Testicular Germ Cell Tumors (TCGA, PanCancer Atlas)
Thymoma (TCGA, PanCancer Atlas)
Thyroid Carcinoma (TCGA, PanCancer Atlas)
Uterine Carcinosarcoma (TCGA, PanCancer Atlas)
Uterine Corpus Endometrial Carcinoma (TCGA, PanCancer Atlas)
Uveal Melanoma (TCGA, PanCancer Atlas)

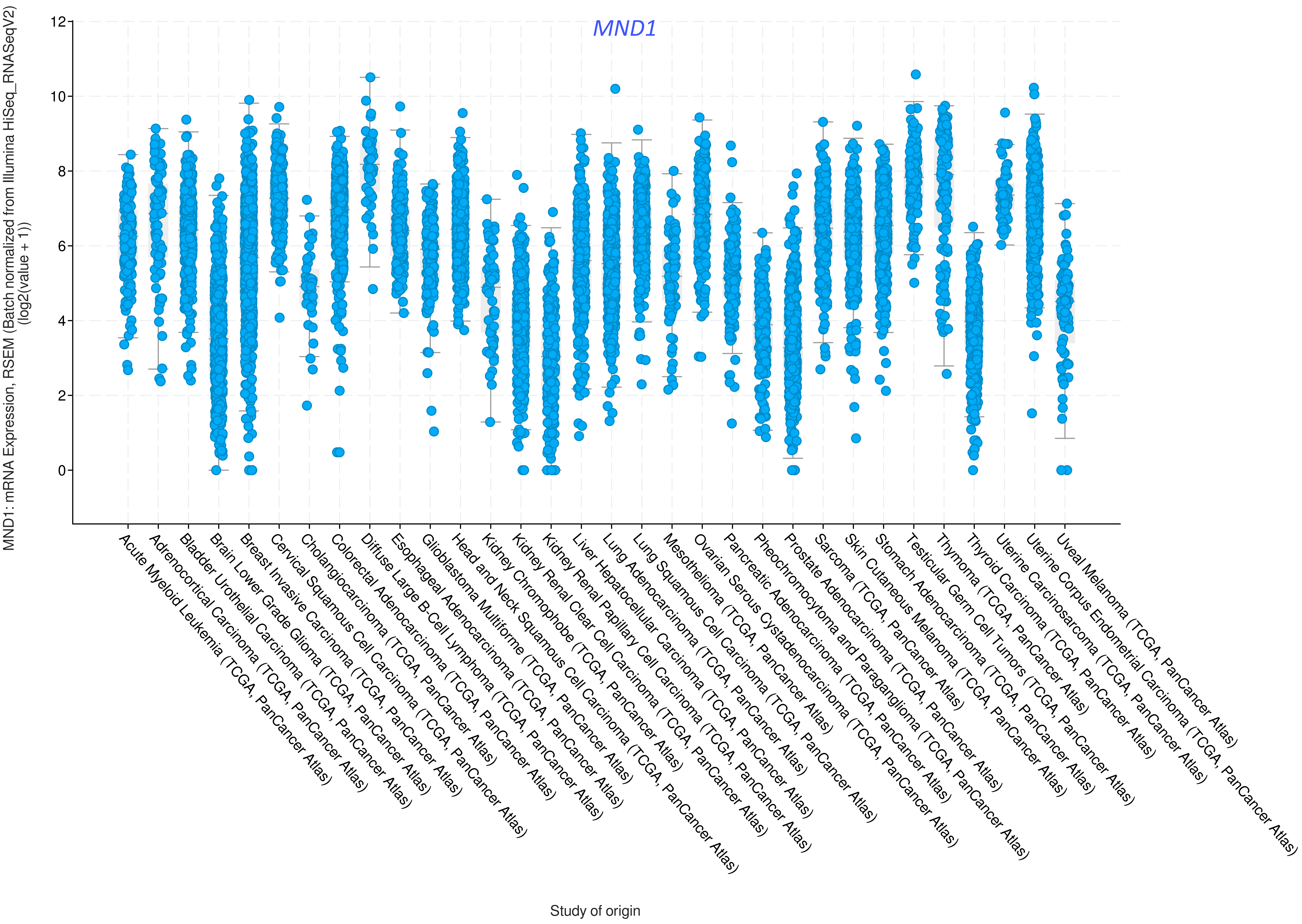

MND1
MND1: mRNA Expression, RSEM (Batch normalized from Illumina HiSeq_RNASeqV2) (log2(value + 1))
12
10
8
6
4
2
0
Acute Myeloid Leukemia (TCGA, PanCancer Atlas)
Adrenocortical Carcinoma (TCGA, PanCancer Atlas)
Bladder Urothelial Carcinoma (TCGA, PanCancer Atlas)
Brain Lower Grade Glioma (TCGA, PanCancer Atlas)
Breast Invasive Carcinoma (TCGA, PanCancer Atlas)
Cervical Squamous Cell Carcinoma (TCGA, PanCancer Atlas)
Cholangiocarcinoma (TCGA, PanCancer Atlas)
Colorectal Adenocarcinoma (TCGA, PanCancer Atlas)
Diffuse Large B-Cell Lymphoma (TCGA, PanCancer Atlas)
Esophageal Adenocarcinoma (TCGA, PanCancer Atlas)
Glioblastoma Multiforme (TCGA, PanCancer Atlas)
Head and Neck Squamous Cell Carcinoma (TCGA, PanCancer Atlas)
Kidney Chromophobe (TCGA, PanCancer Atlas)
Kidney Renal Clear Cell Carcinoma (TCGA, PanCancer Atlas)
Kidney Renal Papillary Cell Carcinoma (TCGA, PanCancer Atlas)
Liver Hepatocellular Carcinoma (TCGA, PanCancer Atlas)
Lung Adenocarcinoma (TCGA, PanCancer Atlas)
Lung Squamous Cell Carcinoma (TCGA, PanCancer Atlas)
Mesothelioma (TCGA, PanCancer Atlas)
Ovarian Serous Cystadenocarcinoma (TCGA, PanCancer Atlas)
Pancreatic Adenocarcinoma (TCGA, PanCancer Atlas)
Pheochromocytoma and Paraganglioma (TCGA, PanCancer Atlas)
Prostate Adenocarcinoma (TCGA, PanCancer Atlas)
Sarcoma (TCGA, PanCancer Atlas)
Skin Cutaneous Melanoma (TCGA, PanCancer Atlas)
Stomach Adenocarcinoma (TCGA, PanCancer Atlas)
Testicular Germ Cell Tumors (TCGA, PanCancer Atlas)
Thymoma (TCGA, PanCancer Atlas)
Thyroid Carcinoma (TCGA, PanCancer Atlas)
Uterine Carcinosarcoma (TCGA, PanCancer Atlas)
Uterine Corpus Endometrial Carcinoma (TCGA, PanCancer Atlas)
Uveal Melanoma (TCGA, PanCancer Atlas)
Study of origin

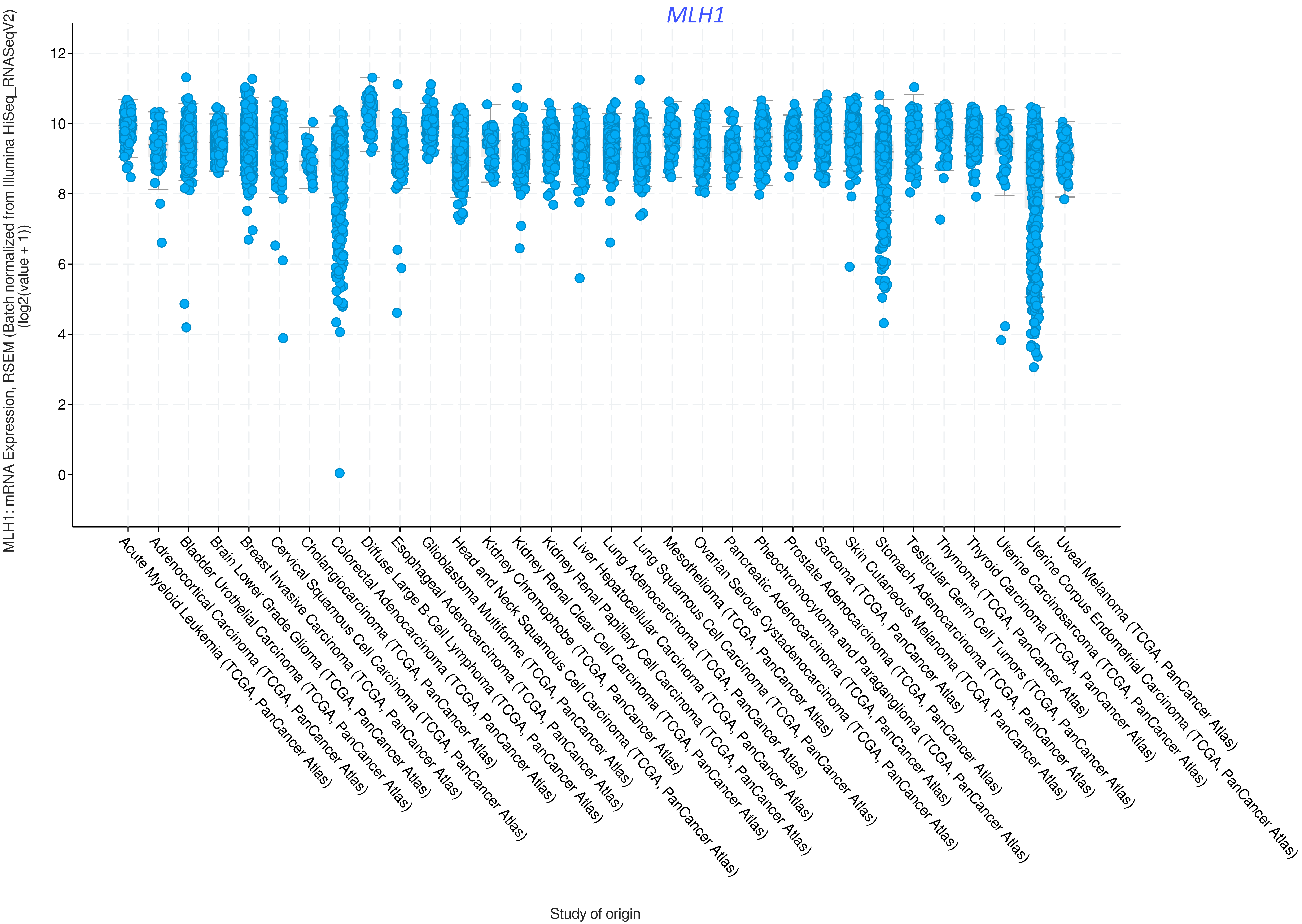
MLH1
MLH1: mRNA Expression, RSEM (Batch normalized from Illumina HiSeq_RNASeqV2) (log2(value + 1))
Study of origin
12
10
8
6
4
2
0
Acute Myeloid Leukemia (TCGA, PanCancer Atlas)
Adrenocortical Carcinoma (TCGA, PanCancer Atlas)
Bladder Urothelial Carcinoma (TCGA, PanCancer Atlas)
Brain Lower Grade Glioma (TCGA, PanCancer Atlas)
Breast Invasive Carcinoma (TCGA, PanCancer Atlas)
Cervical Squamous Cell Carcinoma (TCGA, PanCancer Atlas)
Cholangiocarcinoma (TCGA, PanCancer Atlas)
Colorectal Adenocarcinoma (TCGA, PanCancer Atlas)
Diffuse Large B-Cell Lymphoma (TCGA, PanCancer Atlas)
Esophageal Adenocarcinoma (TCGA, PanCancer Atlas)
Glioblastoma Multiforme (TCGA, PanCancer Atlas)
Head and Neck Squamous Cell Carcinoma (TCGA, PanCancer Atlas)
Kidney Chromophobe (TCGA, PanCancer Atlas)
Kidney Renal Clear Cell Carcinoma (TCGA, PanCancer Atlas)
Kidney Renal Papillary Cell Carcinoma (TCGA, PanCancer Atlas)
Liver Hepatocellular Carcinoma (TCGA, PanCancer Atlas)
Lung Adenocarcinoma (TCGA, PanCancer Atlas)
Lung Squamous Cell Carcinoma (TCGA, PanCancer Atlas)
Mesothelioma (TCGA, PanCancer Atlas)
Ovarian Serous Cystadenocarcinoma (TCGA, PanCancer Atlas)
Pancreatic Adenocarcinoma (TCGA, PanCancer Atlas)
Pheochromocytoma and Paraganglioma (TCGA, PanCancer Atlas)
Prostate Adenocarcinoma (TCGA, PanCancer Atlas)
Sarcoma (TCGA, PanCancer Atlas)
Skin Cutaneous Melanoma (TCGA, PanCancer Atlas)
Stomach Adenocarcinoma (TCGA, PanCancer Atlas)
Testicular Germ Cell Tumors (TCGA, PanCancer Atlas)
Thymoma (TCGA, PanCancer Atlas)
Thyroid Carcinoma (TCGA, PanCancer Atlas)
Uterine Carcinosarcoma (TCGA, PanCancer Atlas)
Uterine Corpus Endometrial Carcinoma (TCGA, PanCancer Atlas)
Uveal Melanoma (TCGA, PanCancer Atlas)

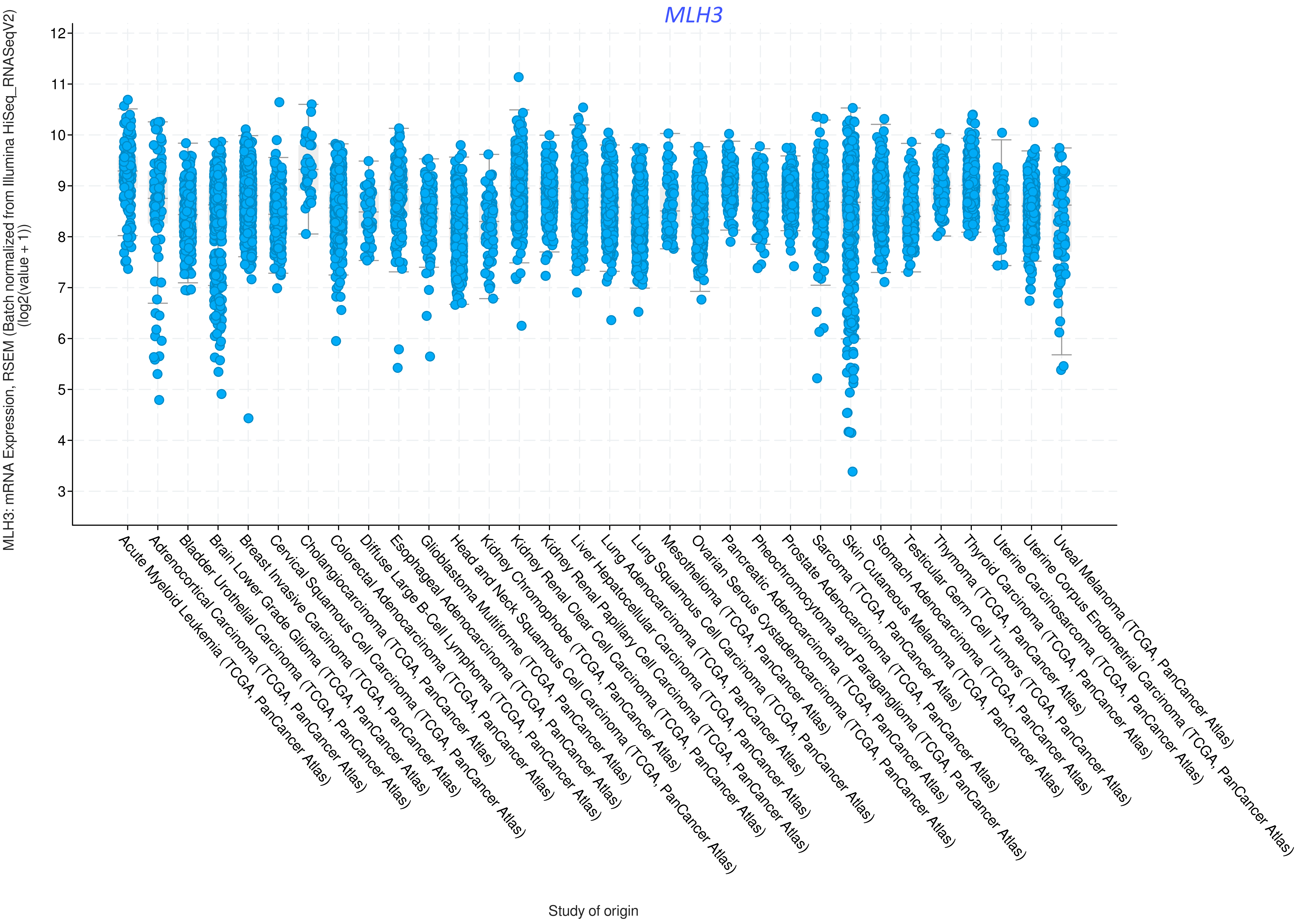
MLH3
MLH3: mRNA Expression, RSEM (Batch normalized from Illumina HiSeq_RNASeqV2) (log2(value + 1))
Study of origin
Acute Myeloid Leukemia (TCGA, PanCancer Atlas)
Adrenocortical Carcinoma (TCGA, PanCancer Atlas)
Bladder Urothelial Carcinoma (TCGA, PanCancer Atlas)
Brain Lower Grade Glioma (TCGA, PanCancer Atlas)
Breast Invasive Carcinoma (TCGA, PanCancer Atlas)
Cervical Squamous Cell Carcinoma (TCGA, PanCancer Atlas)
Cholangiocarcinoma (TCGA, PanCancer Atlas)
Colorectal Adenocarcinoma (TCGA, PanCancer Atlas)
Diffuse Large B-Cell Lymphoma (TCGA, PanCancer Atlas)
Esophageal Adenocarcinoma (TCGA, PanCancer Atlas)
Glioblastoma Multiforme (TCGA, PanCancer Atlas)
Head and Neck Squamous Cell Carcinoma (TCGA, PanCancer Atlas)
Kidney Chromophobe (TCGA, PanCancer Atlas)
Kidney Renal Clear Cell Carcinoma (TCGA, PanCancer Atlas)
Kidney Renal Papillary Cell Carcinoma (TCGA, PanCancer Atlas)
Liver Hepatocellular Carcinoma (TCGA, PanCancer Atlas)
Lung Adenocarcinoma (TCGA, PanCancer Atlas)
Lung Squamous Cell Carcinoma (TCGA, PanCancer Atlas)
Mesothelioma (TCGA, PanCancer Atlas)
Ovarian Serous Cystadenocarcinoma (TCGA, PanCancer Atlas)
Pancreatic Adenocarcinoma (TCGA, PanCancer Atlas)
Pheochromocytoma and Paraganglioma (TCGA, PanCancer Atlas)
Prostate Adenocarcinoma (TCGA, PanCancer Atlas)
Sarcoma (TCGA, PanCancer Atlas)
Skin Cutaneous Melanoma (TCGA, PanCancer Atlas)
Stomach Adenocarcinoma (TCGA, PanCancer Atlas)
Testicular Germ Cell Tumors (TCGA, PanCancer Atlas)
Thymoma (TCGA, PanCancer Atlas)
Thyroid Carcinoma (TCGA, PanCancer Atlas)
Uterine Carcinosarcoma (TCGA, PanCancer Atlas)
Uterine Corpus Endometrial Carcinoma (TCGA, PanCancer Atlas)
Uveal Melanoma (TCGA, PanCancer Atlas)
3
4
5
6
7
8
9
10
11
12

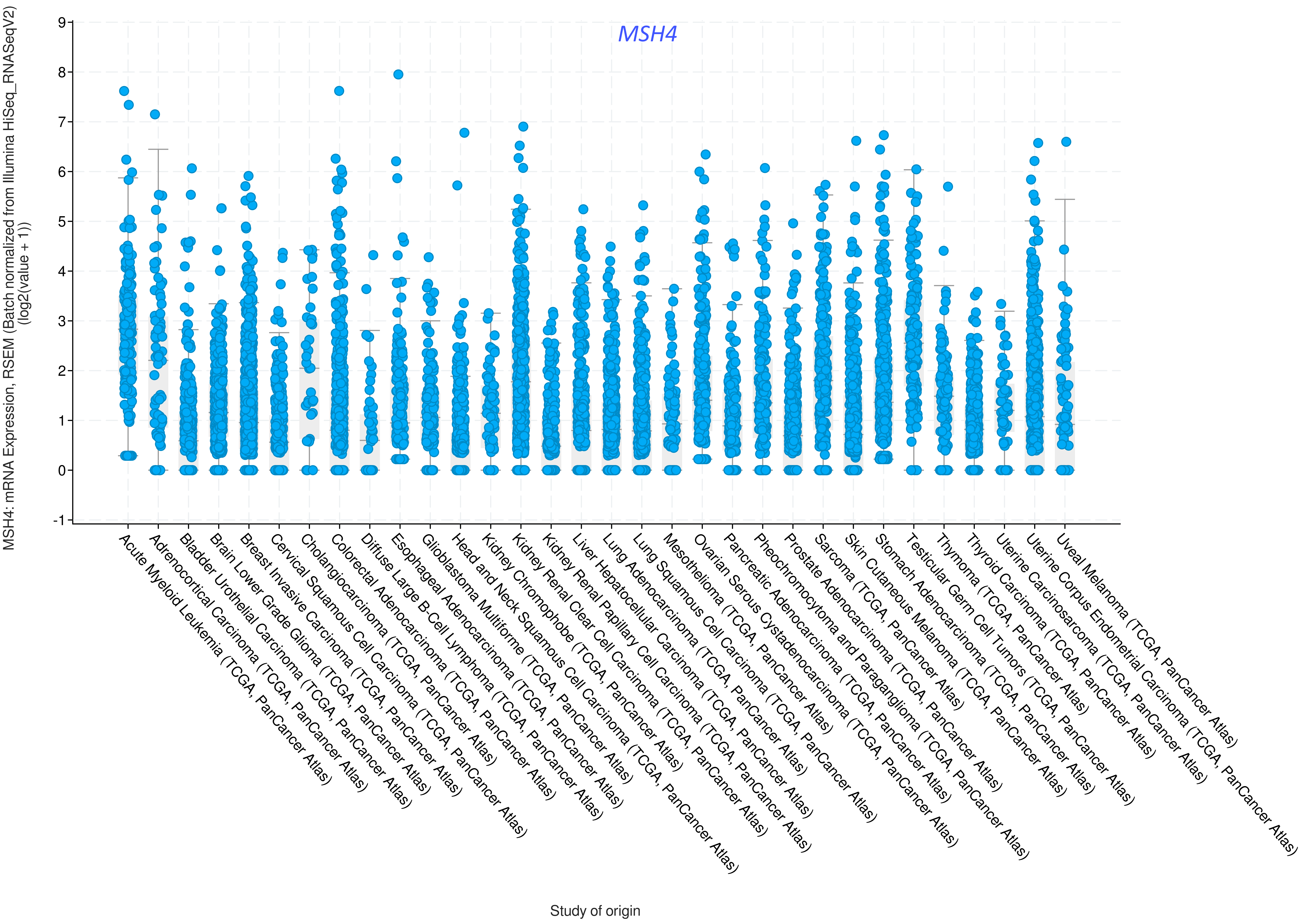

MSH4
MSH4: mRNA Expression, RSEM (Batch normalized from Illumina HiSeq_RNASeqV2) (log2(value + 1))
9
8
7
6
5
4
3
2
1
0
-1
Acute Myeloid Leukemia (TCGA, PanCancer Atlas)
Adrenocortical Carcinoma (TCGA, PanCancer Atlas)
Bladder Urothelial Carcinoma (TCGA, PanCancer Atlas)
Brain Lower Grade Glioma (TCGA, PanCancer Atlas)
Breast Invasive Carcinoma (TCGA, PanCancer Atlas)
Cervical Squamous Cell Carcinoma (TCGA, PanCancer Atlas)
Cholangiocarcinoma (TCGA, PanCancer Atlas)
Colorectal Adenocarcinoma (TCGA, PanCancer Atlas)
Diffuse Large B-Cell Lymphoma (TCGA, PanCancer Atlas)
Esophageal Adenocarcinoma (TCGA, PanCancer Atlas)
Glioblastoma Multiforme (TCGA, PanCancer Atlas)
Head and Neck Squamous Cell Carcinoma (TCGA, PanCancer Atlas)
Kidney Chromophobe (TCGA, PanCancer Atlas)
Kidney Renal Clear Cell Carcinoma (TCGA, PanCancer Atlas)
Kidney Renal Papillary Cell Carcinoma (TCGA, PanCancer Atlas)
Liver Hepatocellular Carcinoma (TCGA, PanCancer Atlas)
Lung Adenocarcinoma (TCGA, PanCancer Atlas)
Lung Squamous Cell Carcinoma (TCGA, PanCancer Atlas)
Mesothelioma (TCGA, PanCancer Atlas)
Ovarian Serous Cystadenocarcinoma (TCGA, PanCancer Atlas)
Pancreatic Adenocarcinoma (TCGA, PanCancer Atlas)
Pheochromocytoma and Paraganglioma (TCGA, PanCancer Atlas)
Prostate Adenocarcinoma (TCGA, PanCancer Atlas)
Sarcoma (TCGA, PanCancer Atlas)
Skin Cutaneous Melanoma (TCGA, PanCancer Atlas)
Stomach Adenocarcinoma (TCGA, PanCancer Atlas)
Testicular Germ Cell Tumors (TCGA, PanCancer Atlas)
Thymoma (TCGA, PanCancer Atlas)
Thyroid Carcinoma (TCGA, PanCancer Atlas)
Uterine Carcinosarcoma (TCGA, PanCancer Atlas)
Uterine Corpus Endometrial Carcinoma (TCGA, PanCancer Atlas)
Uveal Melanoma (TCGA, PanCancer Atlas)
Study of origin

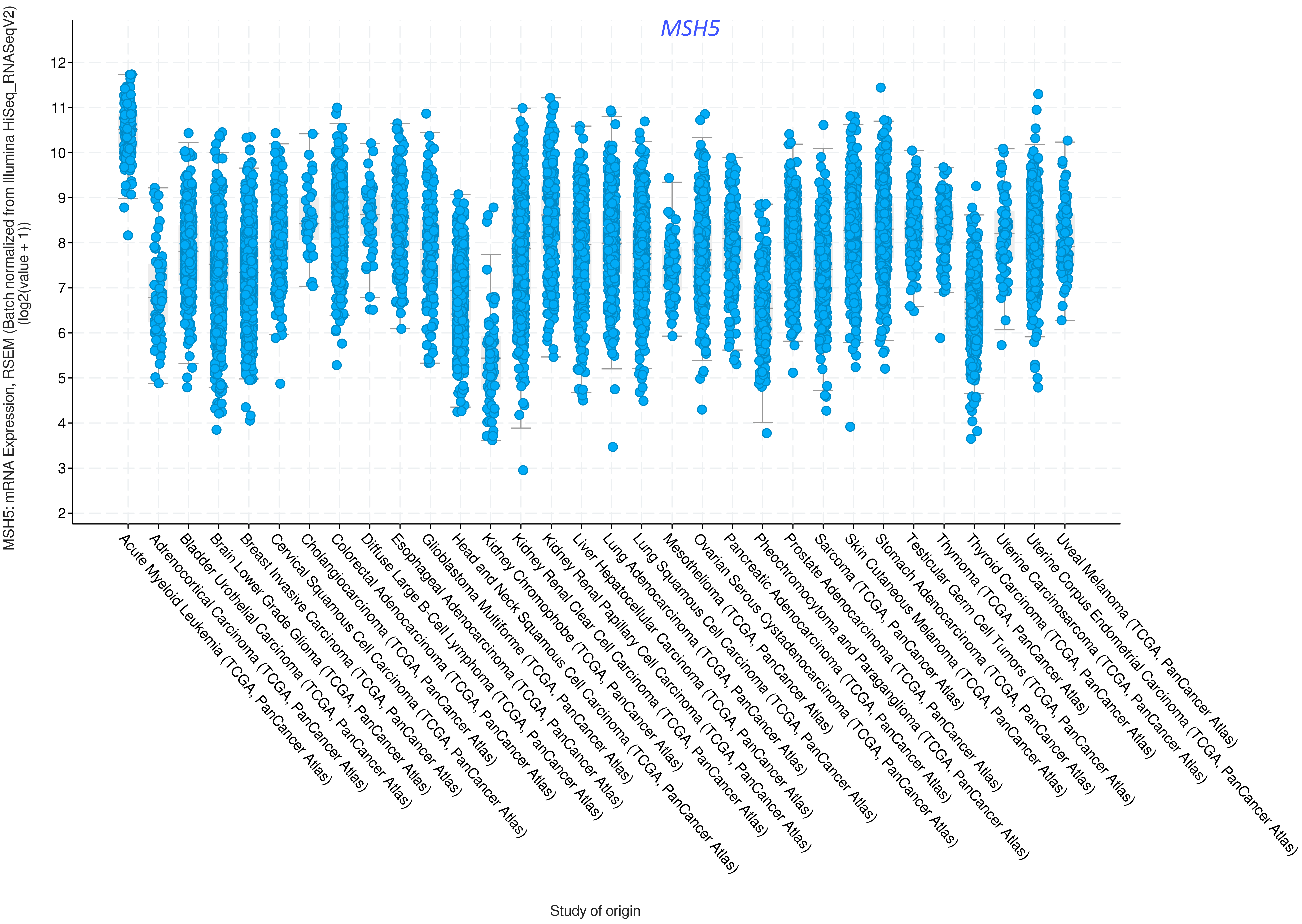
MSH5
MSH5: mRNA Expression, RSEM (Batch normalized from Illumina HiSeq_RNASeqV2) (log2(value + 1))
12
11
10
9
8
7
6
5
4
3
2
Acute Myeloid Leukemia (TCGA, PanCancer Atlas)
Adrenocortical Carcinoma (TCGA, PanCancer Atlas)
Bladder Urothelial Carcinoma (TCGA, PanCancer Atlas)
Brain Lower Grade Glioma (TCGA, PanCancer Atlas)
Breast Invasive Carcinoma (TCGA, PanCancer Atlas)
Cervical Squamous Cell Carcinoma (TCGA, PanCancer Atlas)
Cholangiocarcinoma (TCGA, PanCancer Atlas)
Colorectal Adenocarcinoma (TCGA, PanCancer Atlas)
Diffuse Large B-Cell Lymphoma (TCGA, PanCancer Atlas)
Esophageal Adenocarcinoma (TCGA, PanCancer Atlas)
Glioblastoma Multiforme (TCGA, PanCancer Atlas)
Head and Neck Squamous Cell Carcinoma (TCGA, PanCancer Atlas)
Kidney Chromophobe (TCGA, PanCancer Atlas)
Kidney Renal Clear Cell Carcinoma (TCGA, PanCancer Atlas)
Kidney Renal Papillary Cell Carcinoma (TCGA, PanCancer Atlas)
Liver Hepatocellular Carcinoma (TCGA, PanCancer Atlas)
Lung Adenocarcinoma (TCGA, PanCancer Atlas)
Lung Squamous Cell Carcinoma (TCGA, PanCancer Atlas)
Mesothelioma (TCGA, PanCancer Atlas)
Ovarian Serous Cystadenocarcinoma (TCGA, PanCancer Atlas)
Pancreatic Adenocarcinoma (TCGA, PanCancer Atlas)
Pheochromocytoma and Paraganglioma (TCGA, PanCancer Atlas)
Prostate Adenocarcinoma (TCGA, PanCancer Atlas)
Sarcoma (TCGA, PanCancer Atlas)
Skin Cutaneous Melanoma (TCGA, PanCancer Atlas)
Stomach Adenocarcinoma (TCGA, PanCancer Atlas)
Testicular Germ Cell Tumors (TCGA, PanCancer Atlas)
Thymoma (TCGA, PanCancer Atlas)
Thyroid Carcinoma (TCGA, PanCancer Atlas)
Uterine Carcinosarcoma (TCGA, PanCancer Atlas)
Uterine Corpus Endometrial Carcinoma (TCGA, PanCancer Atlas)
Uveal Melanoma (TCGA, PanCancer Atlas)
Study of origin

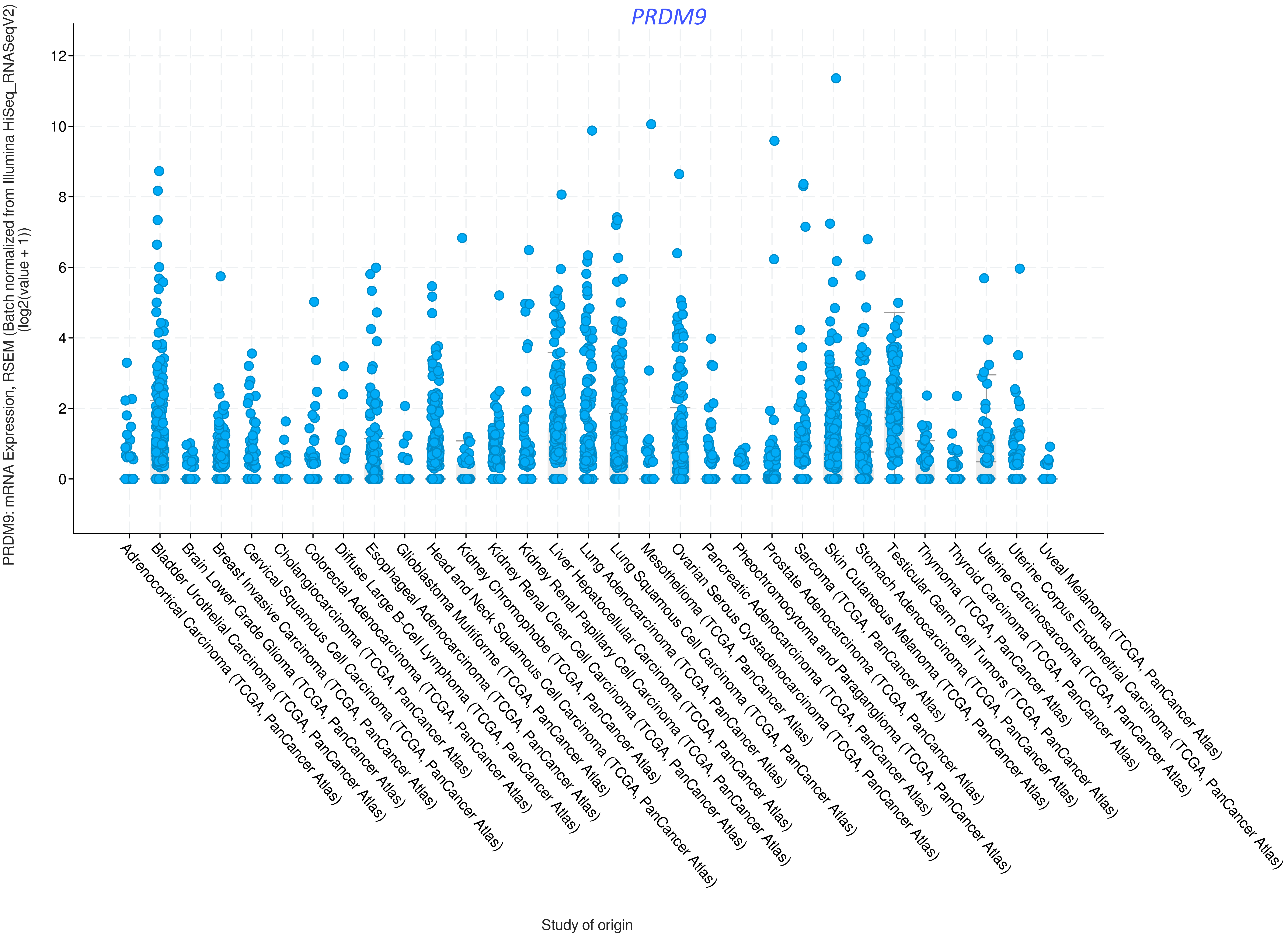

PRDM9
PRDM9: mRNA Expression, RSEM (Batch normalized from Illumina HiSeq_RNASeqV2) (log2(value + 1))
0
2
4
6
8
10
12
Adrenocortical Carcinoma (TCGA, PanCancer Atlas)
Bladder Urothelial Carcinoma (TCGA, PanCancer Atlas)
Brain Lower Grade Glioma (TCGA, PanCancer Atlas)
Breast Invasive Carcinoma (TCGA, PanCancer Atlas)
Cervical Squamous Cell Carcinoma (TCGA, PanCancer Atlas)
Cholangiocarcinoma (TCGA, PanCancer Atlas)
Colorectal Adenocarcinoma (TCGA, PanCancer Atlas)
Diffuse Large B-Cell Lymphoma (TCGA, PanCancer Atlas)
Esophageal Adenocarcinoma (TCGA, PanCancer Atlas)
Glioblastoma Multiforme (TCGA, PanCancer Atlas)
Head and Neck Squamous Cell Carcinoma (TCGA, PanCancer Atlas)
Kidney Chromophobe (TCGA, PanCancer Atlas)
Kidney Renal Clear Cell Carcinoma (TCGA, PanCancer Atlas)
Kidney Renal Papillary Cell Carcinoma (TCGA, PanCancer Atlas)
Liver Hepatocellular Carcinoma (TCGA, PanCancer Atlas)
Lung Adenocarcinoma (TCGA, PanCancer Atlas)
Lung Squamous Cell Carcinoma (TCGA, PanCancer Atlas)
Mesothelioma (TCGA, PanCancer Atlas)
Ovarian Serous Cystadenocarcinoma (TCGA, PanCancer Atlas)
Pancreatic Adenocarcinoma (TCGA, PanCancer Atlas)
Pheochromocytoma and Paraganglioma (TCGA, PanCancer Atlas)
Prostate Adenocarcinoma (TCGA, PanCancer Atlas)
Sarcoma (TCGA, PanCancer Atlas)
Skin Cutaneous Melanoma (TCGA, PanCancer Atlas)
Stomach Adenocarcinoma (TCGA, PanCancer Atlas)
Testicular Germ Cell Tumors (TCGA, PanCancer Atlas)
Thymoma (TCGA, PanCancer Atlas)
Thyroid Carcinoma (TCGA, PanCancer Atlas)
Uterine Carcinosarcoma (TCGA, PanCancer Atlas)
Uterine Corpus Endometrial Carcinoma (TCGA, PanCancer Atlas)
Uveal Melanoma (TCGA, PanCancer Atlas)
Study of origin

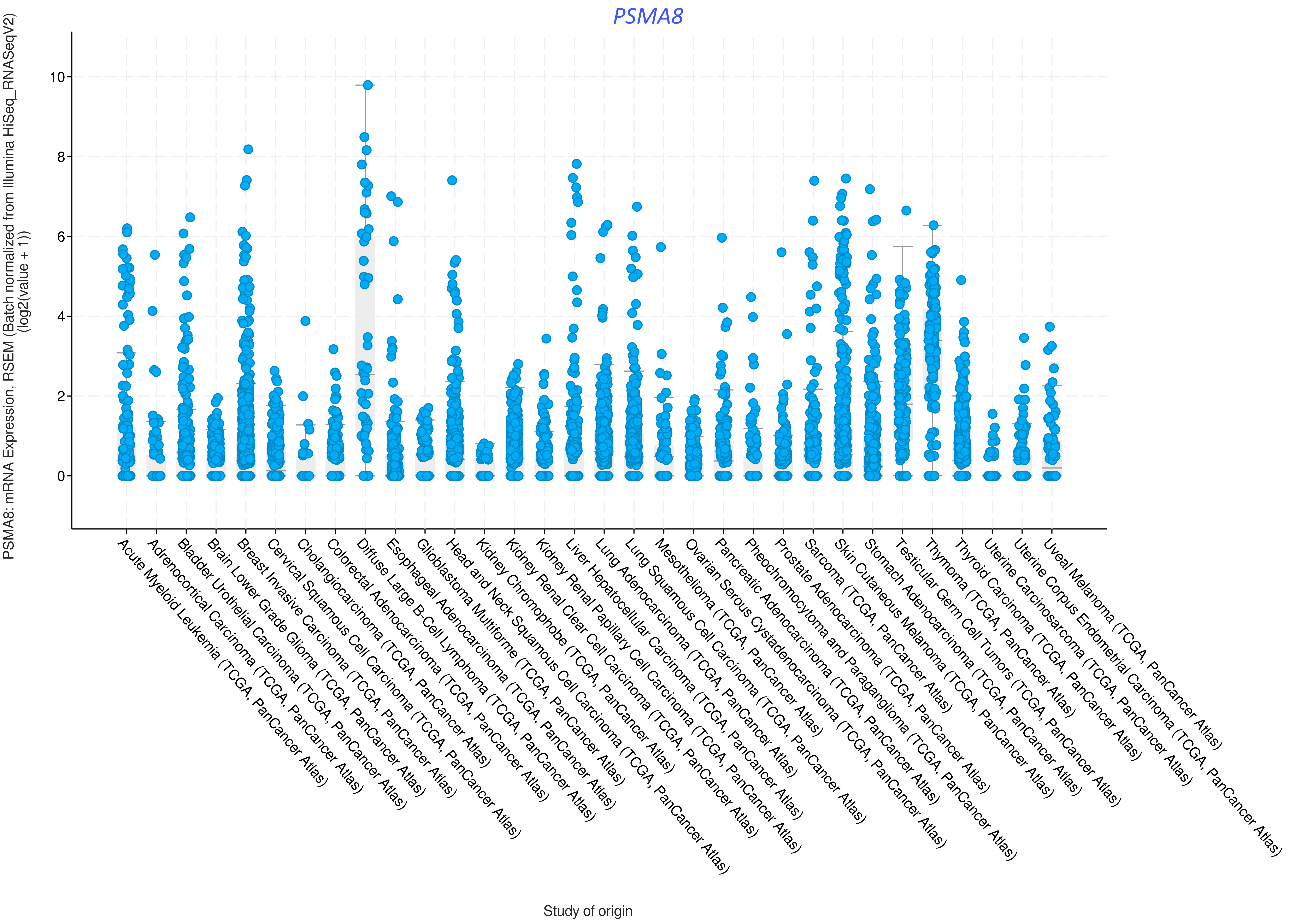

PSMA8
PSMA8: mRNA Expression, RSEM (Batch normalized from Illumina HiSeq_RNASeqV2) (log2(value + 1))
10
8
6
4
2
0
Acute Myeloid Leukemia (TCGA, PanCancer Atlas)
Adrenocortical Carcinoma (TCGA, PanCancer Atlas)
Bladder Urothelial Carcinoma (TCGA, PanCancer Atlas)
Brain Lower Grade Glioma (TCGA, PanCancer Atlas)
Breast Invasive Carcinoma (TCGA, PanCancer Atlas)
Cervical Squamous Cell Carcinoma (TCGA, PanCancer Atlas)
Cholangiocarcinoma (TCGA, PanCancer Atlas)
Colorectal Adenocarcinoma (TCGA, PanCancer Atlas)
Diffuse Large B-Cell Lymphoma (TCGA, PanCancer Atlas)
Esophageal Adenocarcinoma (TCGA, PanCancer Atlas)
Glioblastoma Multiforme (TCGA, PanCancer Atlas)
Head and Neck Squamous Cell Carcinoma (TCGA, PanCancer Atlas)
Kidney Chromophobe (TCGA, PanCancer Atlas)
Kidney Renal Clear Cell Carcinoma (TCGA, PanCancer Atlas)
Kidney Renal Papillary Cell Carcinoma (TCGA, PanCancer Atlas)
Liver Hepatocellular Carcinoma (TCGA, PanCancer Atlas)
Lung Adenocarcinoma (TCGA, PanCancer Atlas)
Lung Squamous Cell Carcinoma (TCGA, PanCancer Atlas)
Mesothelioma (TCGA, PanCancer Atlas)
Ovarian Serous Cystadenocarcinoma (TCGA, PanCancer Atlas)
Pancreatic Adenocarcinoma (TCGA, PanCancer Atlas)
Pheochromocytoma and Paraganglioma (TCGA, PanCancer Atlas)
Prostate Adenocarcinoma (TCGA, PanCancer Atlas)
Sarcoma (TCGA, PanCancer Atlas)
Skin Cutaneous Melanoma (TCGA, PanCancer Atlas)
Stomach Adenocarcinoma (TCGA, PanCancer Atlas)
Testicular Germ Cell Tumors (TCGA, PanCancer Atlas)
Thymoma (TCGA, PanCancer Atlas)
Thyroid Carcinoma (TCGA, PanCancer Atlas)
Uterine Carcinosarcoma (TCGA, PanCancer Atlas)
Uterine Corpus Endometrial Carcinoma (TCGA, PanCancer Atlas)
Uveal Melanoma (TCGA, PanCancer Atlas)
Study of origin

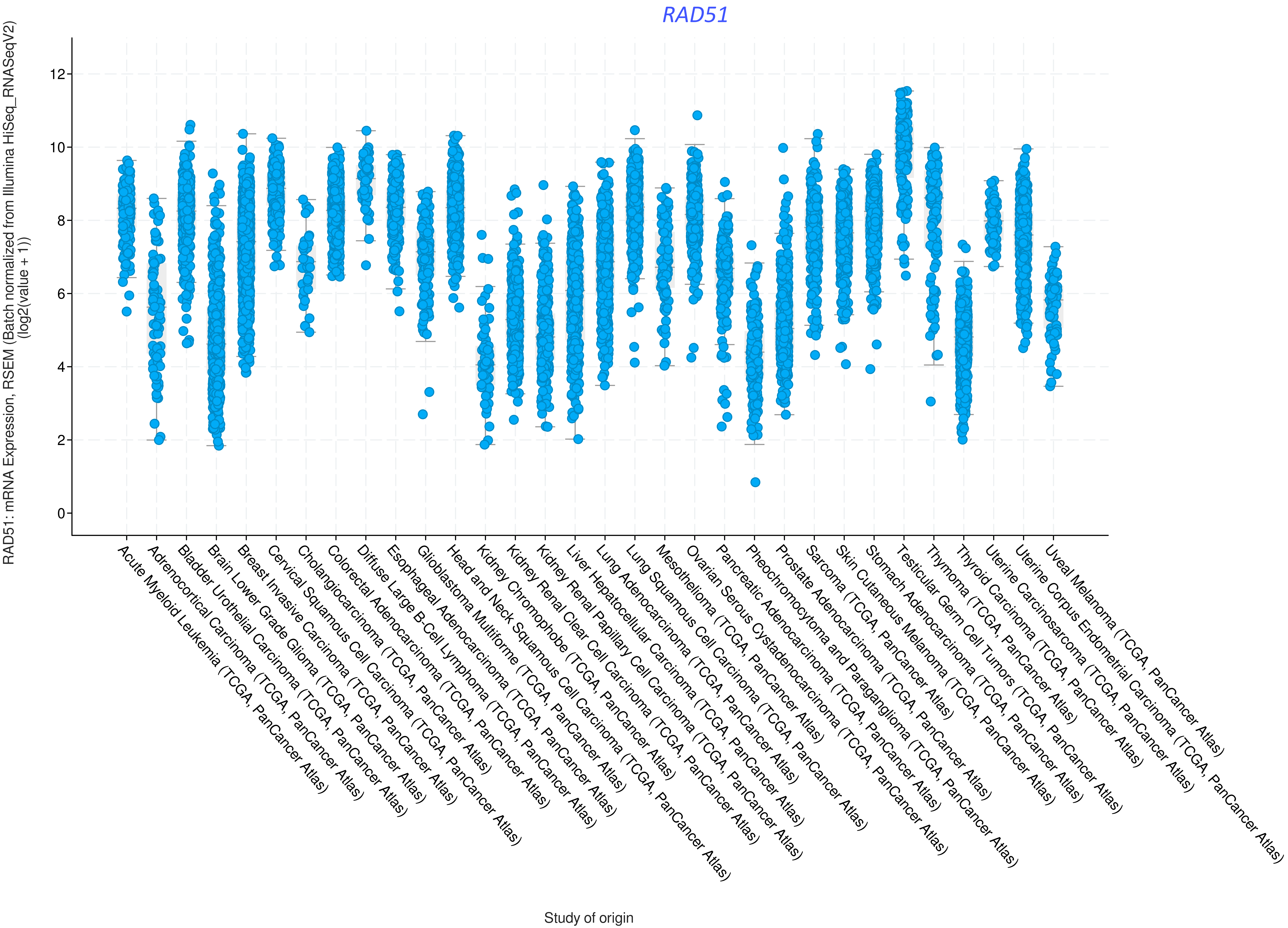
RAD51
RAD51: mRNA Expression, RSEM (Batch normalized from Illumina HiSeq_RNASeqV2) (log2(value + 1))
0
2
4
6
8
10
12
Study of origin
Acute Myeloid Leukemia (TCGA, PanCancer Atlas)
Adrenocortical Carcinoma (TCGA, PanCancer Atlas)
Bladder Urothelial Carcinoma (TCGA, PanCancer Atlas)
Brain Lower Grade Glioma (TCGA, PanCancer Atlas)
Breast Invasive Carcinoma (TCGA, PanCancer Atlas)
Cervical Squamous Cell Carcinoma (TCGA, PanCancer Atlas)
Cholangiocarcinoma (TCGA, PanCancer Atlas)
Colorectal Adenocarcinoma (TCGA, PanCancer Atlas)
Diffuse Large B-Cell Lymphoma (TCGA, PanCancer Atlas)
Esophageal Adenocarcinoma (TCGA, PanCancer Atlas)
Glioblastoma Multiforme (TCGA, PanCancer Atlas)
Head and Neck Squamous Cell Carcinoma (TCGA, PanCancer Atlas)
Kidney Chromophobe (TCGA, PanCancer Atlas)
Kidney Renal Clear Cell Carcinoma (TCGA, PanCancer Atlas)
Kidney Renal Papillary Cell Carcinoma (TCGA, PanCancer Atlas)
Liver Hepatocellular Carcinoma (TCGA, PanCancer Atlas)
Lung Adenocarcinoma (TCGA, PanCancer Atlas)
Lung Squamous Cell Carcinoma (TCGA, PanCancer Atlas)
Mesothelioma (TCGA, PanCancer Atlas)
Ovarian Serous Cystadenocarcinoma (TCGA, PanCancer Atlas)
Pancreatic Adenocarcinoma (TCGA, PanCancer Atlas)
Pheochromocytoma and Paraganglioma (TCGA, PanCancer Atlas)
Prostate Adenocarcinoma (TCGA, PanCancer Atlas)
Sarcoma (TCGA, PanCancer Atlas)
Skin Cutaneous Melanoma (TCGA, PanCancer Atlas)
Stomach Adenocarcinoma (TCGA, PanCancer Atlas)
Testicular Germ Cell Tumors (TCGA, PanCancer Atlas)
Thymoma (TCGA, PanCancer Atlas)
Thyroid Carcinoma (TCGA, PanCancer Atlas)
Uterine Carcinosarcoma (TCGA, PanCancer Atlas)
Uterine Corpus Endometrial Carcinoma (TCGA, PanCancer Atlas)
Uveal Melanoma (TCGA, PanCancer Atlas)

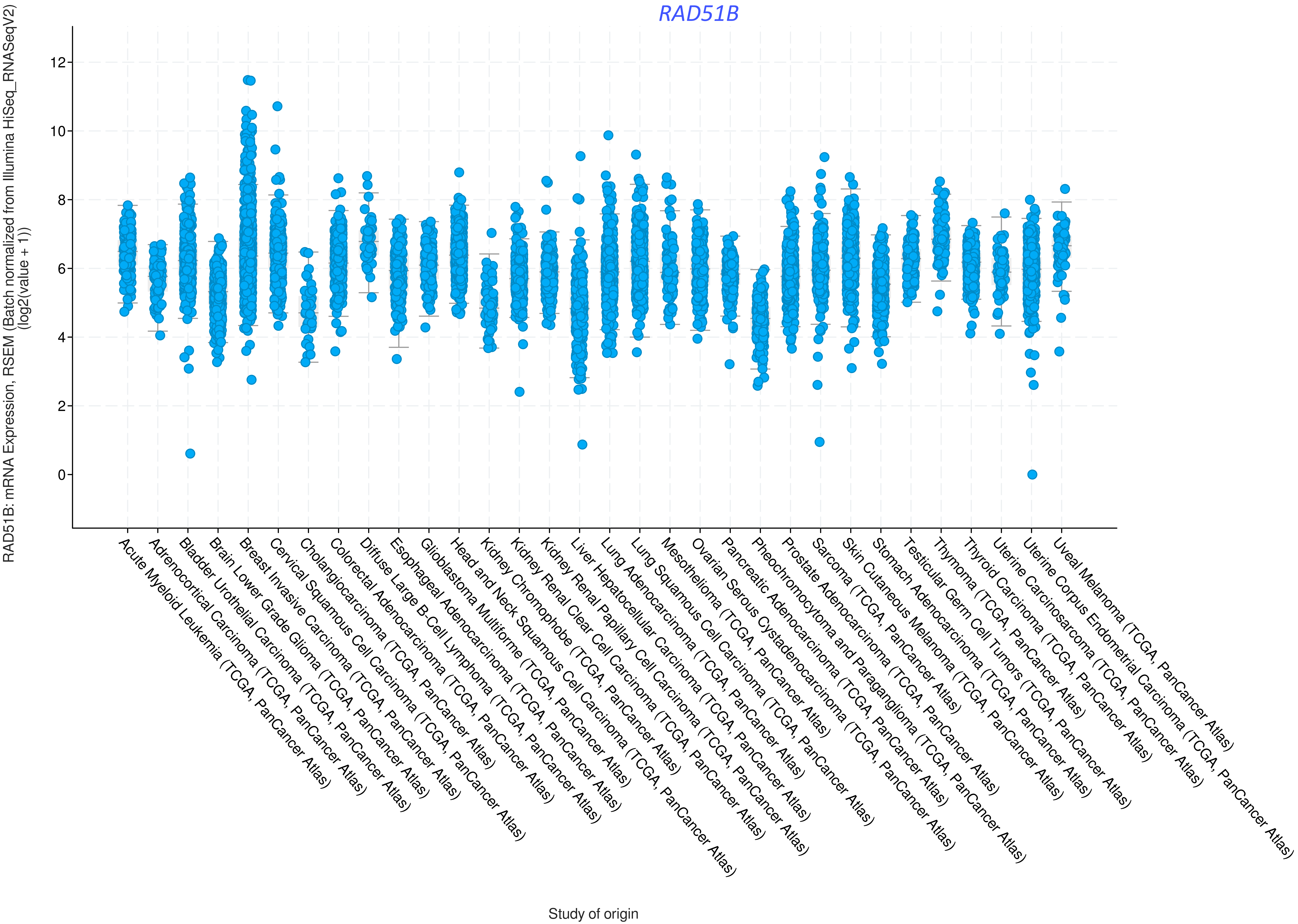

RAD51B
RAD51B: mRNA Expression, RSEM (Batch normalized from Illumina HiSeq_RNASeqV2) (log2(value + 1))
12
10
8
6
4
2
0
Acute Myeloid Leukemia (TCGA, PanCancer Atlas)
Adrenocortical Carcinoma (TCGA, PanCancer Atlas)
Bladder Urothelial Carcinoma (TCGA, PanCancer Atlas)
Brain Lower Grade Glioma (TCGA, PanCancer Atlas)
Breast Invasive Carcinoma (TCGA, PanCancer Atlas)
Cervical Squamous Cell Carcinoma (TCGA, PanCancer Atlas)
Cholangiocarcinoma (TCGA, PanCancer Atlas)
Colorectal Adenocarcinoma (TCGA, PanCancer Atlas)
Diffuse Large B-Cell Lymphoma (TCGA, PanCancer Atlas)
Esophageal Adenocarcinoma (TCGA, PanCancer Atlas)
Glioblastoma Multiforme (TCGA, PanCancer Atlas)
Head and Neck Squamous Cell Carcinoma (TCGA, PanCancer Atlas)
Kidney Chromophobe (TCGA, PanCancer Atlas)
Kidney Renal Clear Cell Carcinoma (TCGA, PanCancer Atlas)
Kidney Renal Papillary Cell Carcinoma (TCGA, PanCancer Atlas)
Liver Hepatocellular Carcinoma (TCGA, PanCancer Atlas)
Lung Adenocarcinoma (TCGA, PanCancer Atlas)
Lung Squamous Cell Carcinoma (TCGA, PanCancer Atlas)
Mesothelioma (TCGA, PanCancer Atlas)
Ovarian Serous Cystadenocarcinoma (TCGA, PanCancer Atlas)
Pancreatic Adenocarcinoma (TCGA, PanCancer Atlas)
Pheochromocytoma and Paraganglioma (TCGA, PanCancer Atlas)
Prostate Adenocarcinoma (TCGA, PanCancer Atlas)
Sarcoma (TCGA, PanCancer Atlas)
Skin Cutaneous Melanoma (TCGA, PanCancer Atlas)
Stomach Adenocarcinoma (TCGA, PanCancer Atlas)
Testicular Germ Cell Tumors (TCGA, PanCancer Atlas)
Thymoma (TCGA, PanCancer Atlas)
Thyroid Carcinoma (TCGA, PanCancer Atlas)
Uterine Carcinosarcoma (TCGA, PanCancer Atlas)
Uterine Corpus Endometrial Carcinoma (TCGA, PanCancer Atlas)
Uveal Melanoma (TCGA, PanCancer Atlas)
Study of origin

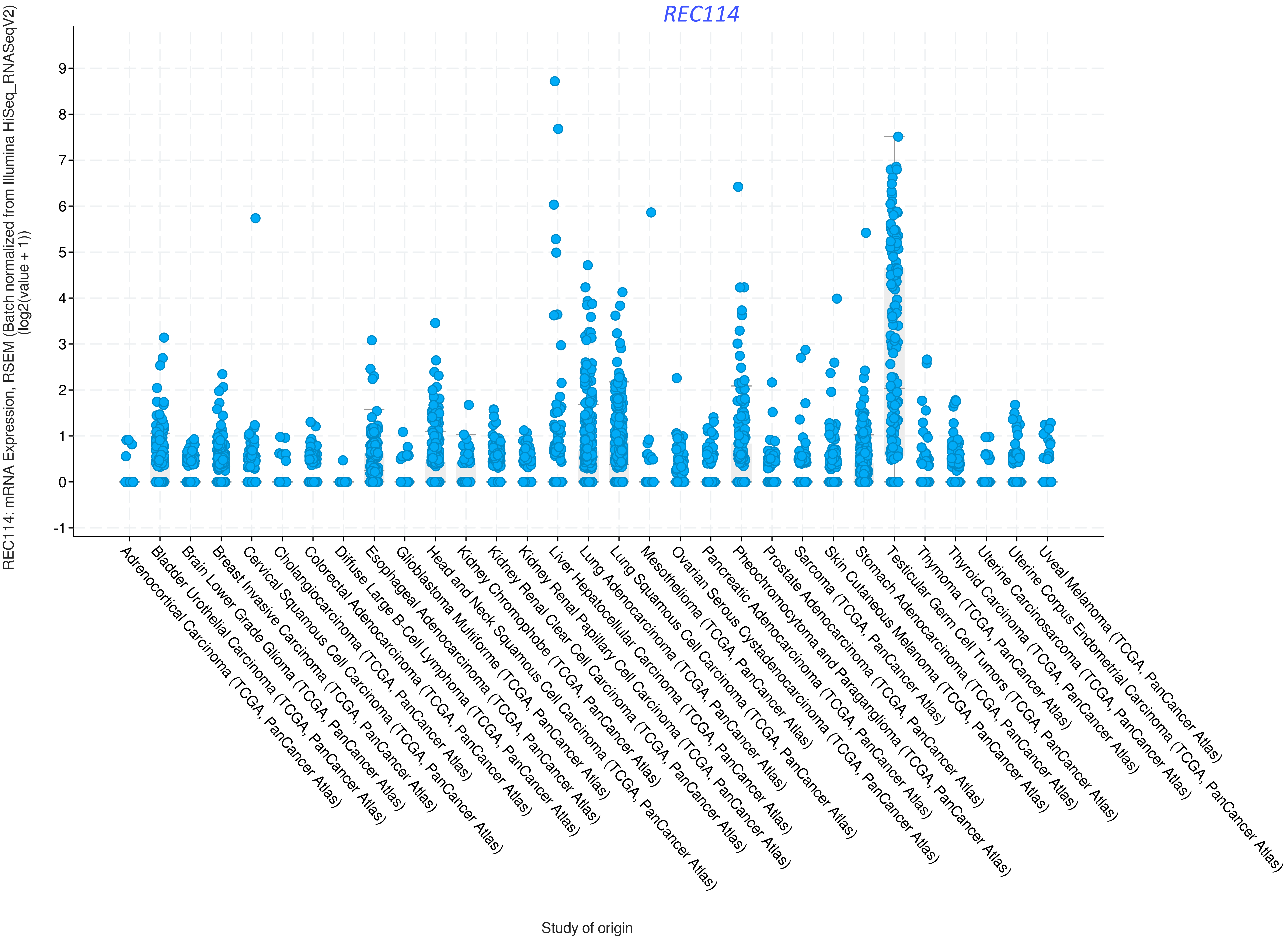
REC114
REC114: mRNA Expression, RSEM (Batch normalized from Illumina HiSeq_RNASeqV2) (log2(value + 1))
9
8
7
6
5
4
3
2
1
0
-1
Adrenocortical Carcinoma (TCGA, PanCancer Atlas)
Bladder Urothelial Carcinoma (TCGA, PanCancer Atlas)
Brain Lower Grade Glioma (TCGA, PanCancer Atlas)
Breast Invasive Carcinoma (TCGA, PanCancer Atlas)
Cervical Squamous Cell Carcinoma (TCGA, PanCancer Atlas)
Cholangiocarcinoma (TCGA, PanCancer Atlas)
Colorectal Adenocarcinoma (TCGA, PanCancer Atlas)
Diffuse Large B-Cell Lymphoma (TCGA, PanCancer Atlas)
Esophageal Adenocarcinoma (TCGA, PanCancer Atlas)
Glioblastoma Multiforme (TCGA, PanCancer Atlas)
Head and Neck Squamous Cell Carcinoma (TCGA, PanCancer Atlas)
Kidney Chromophobe (TCGA, PanCancer Atlas)
Kidney Renal Clear Cell Carcinoma (TCGA, PanCancer Atlas)
Kidney Renal Papillary Cell Carcinoma (TCGA, PanCancer Atlas)
Liver Hepatocellular Carcinoma (TCGA, PanCancer Atlas)
Lung Adenocarcinoma (TCGA, PanCancer Atlas)
Lung Squamous Cell Carcinoma (TCGA, PanCancer Atlas)
Mesothelioma (TCGA, PanCancer Atlas)
Ovarian Serous Cystadenocarcinoma (TCGA, PanCancer Atlas)
Pancreatic Adenocarcinoma (TCGA, PanCancer Atlas)
Pheochromocytoma and Paraganglioma (TCGA, PanCancer Atlas)
Prostate Adenocarcinoma (TCGA, PanCancer Atlas)
Sarcoma (TCGA, PanCancer Atlas)
Skin Cutaneous Melanoma (TCGA, PanCancer Atlas)
Stomach Adenocarcinoma (TCGA, PanCancer Atlas)
Testicular Germ Cell Tumors (TCGA, PanCancer Atlas)
Thymoma (TCGA, PanCancer Atlas)
Thyroid Carcinoma (TCGA, PanCancer Atlas)
Uterine Carcinosarcoma (TCGA, PanCancer Atlas)
Uterine Corpus Endometrial Carcinoma (TCGA, PanCancer Atlas)
Uveal Melanoma (TCGA, PanCancer Atlas)
Study of origin

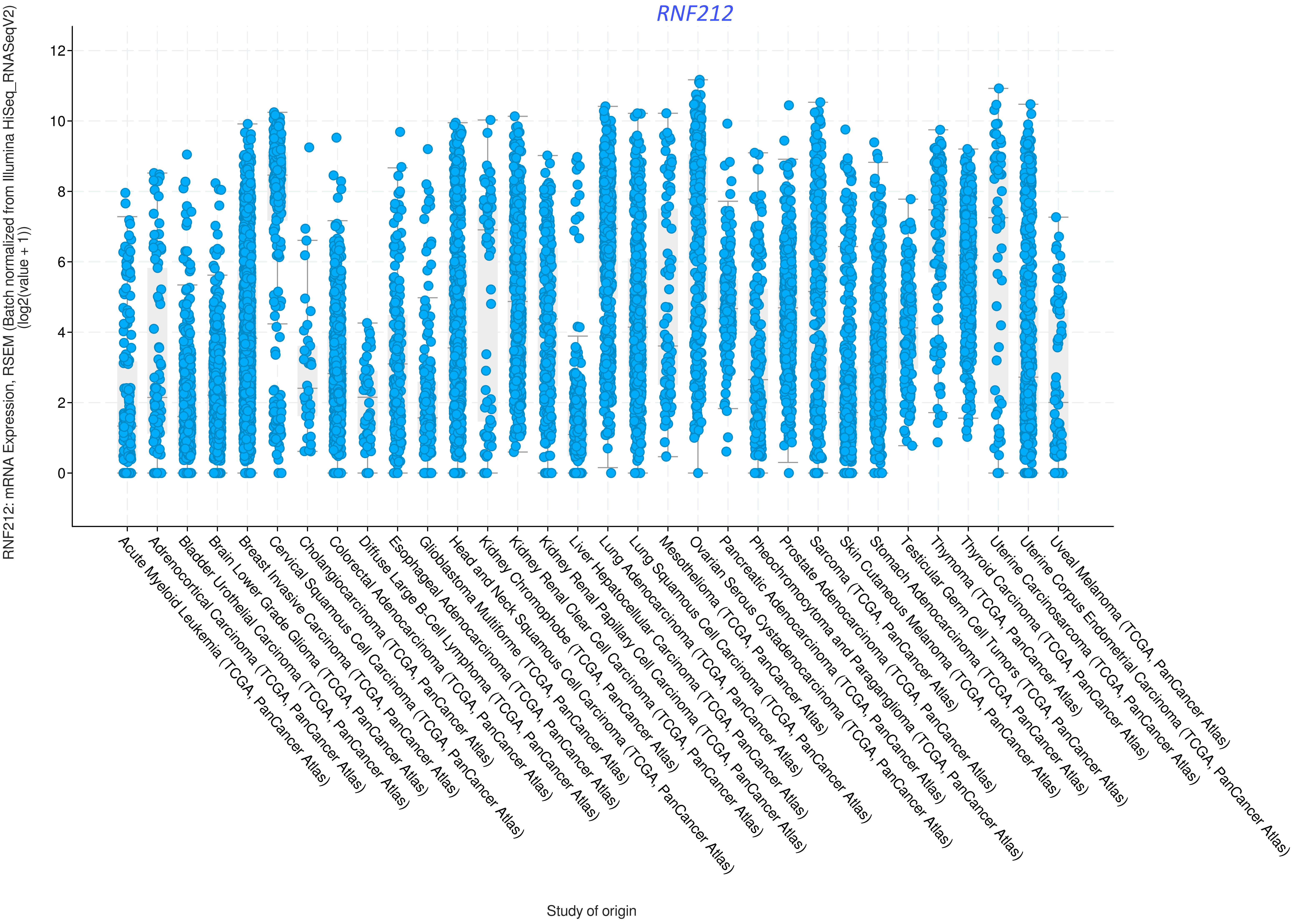

RNF212
RNF212: mRNA Expression, RSEM (Batch normalized from Illumina HiSeq_RNASeqV2) (log2(value + 1))
12
10
8
6
4
2
0
Study of origin
Acute Myeloid Leukemia (TCGA, PanCancer Atlas)
Adrenocortical Carcinoma (TCGA, PanCancer Atlas)
Bladder Urothelial Carcinoma (TCGA, PanCancer Atlas)
Brain Lower Grade Glioma (TCGA, PanCancer Atlas)
Breast Invasive Carcinoma (TCGA, PanCancer Atlas)
Cervical Squamous Cell Carcinoma (TCGA, PanCancer Atlas)
Cholangiocarcinoma (TCGA, PanCancer Atlas)
Colorectal Adenocarcinoma (TCGA, PanCancer Atlas)
Diffuse Large B-Cell Lymphoma (TCGA, PanCancer Atlas)
Esophageal Adenocarcinoma (TCGA, PanCancer Atlas)
Glioblastoma Multiforme (TCGA, PanCancer Atlas)
Head and Neck Squamous Cell Carcinoma (TCGA, PanCancer Atlas)
Kidney Chromophobe (TCGA, PanCancer Atlas)
Kidney Renal Clear Cell Carcinoma (TCGA, PanCancer Atlas)
Kidney Renal Papillary Cell Carcinoma (TCGA, PanCancer Atlas)
Liver Hepatocellular Carcinoma (TCGA, PanCancer Atlas)
Lung Adenocarcinoma (TCGA, PanCancer Atlas)
Lung Squamous Cell Carcinoma (TCGA, PanCancer Atlas)
Mesothelioma (TCGA, PanCancer Atlas)
Ovarian Serous Cystadenocarcinoma (TCGA, PanCancer Atlas)
Pancreatic Adenocarcinoma (TCGA, PanCancer Atlas)
Pheochromocytoma and Paraganglioma (TCGA, PanCancer Atlas)
Prostate Adenocarcinoma (TCGA, PanCancer Atlas)
Sarcoma (TCGA, PanCancer Atlas)
Skin Cutaneous Melanoma (TCGA, PanCancer Atlas)
Stomach Adenocarcinoma (TCGA, PanCancer Atlas)
Testicular Germ Cell Tumors (TCGA, PanCancer Atlas)
Thymoma (TCGA, PanCancer Atlas)
Thyroid Carcinoma (TCGA, PanCancer Atlas)
Uterine Carcinosarcoma (TCGA, PanCancer Atlas)
Uterine Corpus Endometrial Carcinoma (TCGA, PanCancer Atlas)
Uveal Melanoma (TCGA, PanCancer Atlas)

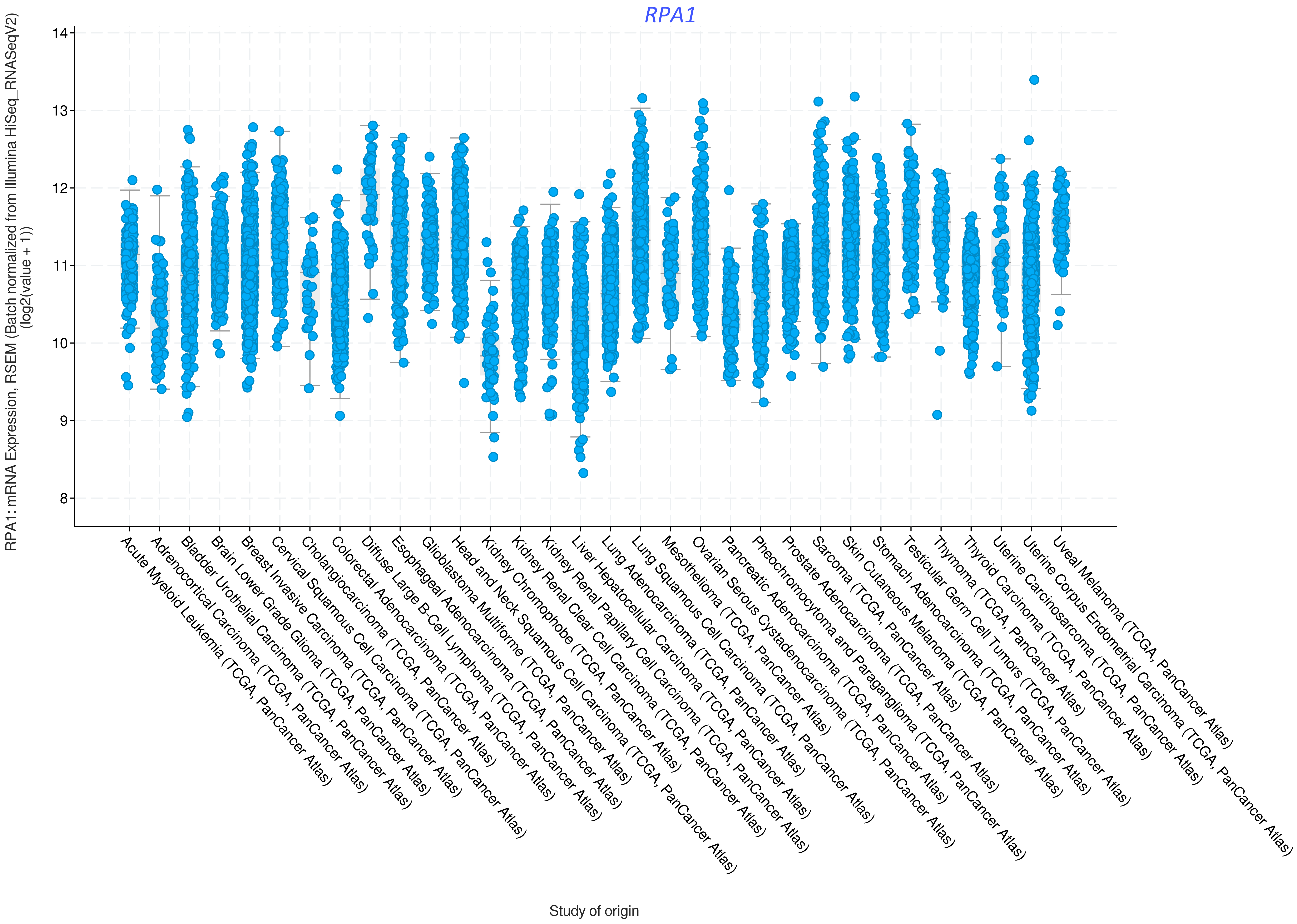
RPA1
RPA1: mRNA Expression, RSEM (Batch normalized from Illumina HiSeq_RNASeqV2) (log2(value + 1))
14
13
12
11
10
9
8
Acute Myeloid Leukemia (TCGA, PanCancer Atlas)
Adrenocortical Carcinoma (TCGA, PanCancer Atlas)
Bladder Urothelial Carcinoma (TCGA, PanCancer Atlas)
Brain Lower Grade Glioma (TCGA, PanCancer Atlas)
Breast Invasive Carcinoma (TCGA, PanCancer Atlas)
Cervical Squamous Cell Carcinoma (TCGA, PanCancer Atlas)
Cholangiocarcinoma (TCGA, PanCancer Atlas)
Colorectal Adenocarcinoma (TCGA, PanCancer Atlas)
Diffuse Large B-Cell Lymphoma (TCGA, PanCancer Atlas)
Esophageal Adenocarcinoma (TCGA, PanCancer Atlas)
Glioblastoma Multiforme (TCGA, PanCancer Atlas)
Head and Neck Squamous Cell Carcinoma (TCGA, PanCancer Atlas)
Kidney Chromophobe (TCGA, PanCancer Atlas)
Kidney Renal Clear Cell Carcinoma (TCGA, PanCancer Atlas)
Kidney Renal Papillary Cell Carcinoma (TCGA, PanCancer Atlas)
Liver Hepatocellular Carcinoma (TCGA, PanCancer Atlas)
Lung Adenocarcinoma (TCGA, PanCancer Atlas)
Lung Squamous Cell Carcinoma (TCGA, PanCancer Atlas)
Mesothelioma (TCGA, PanCancer Atlas)
Ovarian Serous Cystadenocarcinoma (TCGA, PanCancer Atlas)
Pancreatic Adenocarcinoma (TCGA, PanCancer Atlas)
Pheochromocytoma and Paraganglioma (TCGA, PanCancer Atlas)
Prostate Adenocarcinoma (TCGA, PanCancer Atlas)
Sarcoma (TCGA, PanCancer Atlas)
Skin Cutaneous Melanoma (TCGA, PanCancer Atlas)
Stomach Adenocarcinoma (TCGA, PanCancer Atlas)
Testicular Germ Cell Tumors (TCGA, PanCancer Atlas)
Thymoma (TCGA, PanCancer Atlas)
Thyroid Carcinoma (TCGA, PanCancer Atlas)
Uterine Carcinosarcoma (TCGA, PanCancer Atlas)
Uterine Corpus Endometrial Carcinoma (TCGA, PanCancer Atlas)
Uveal Melanoma (TCGA, PanCancer Atlas)
Study of origin

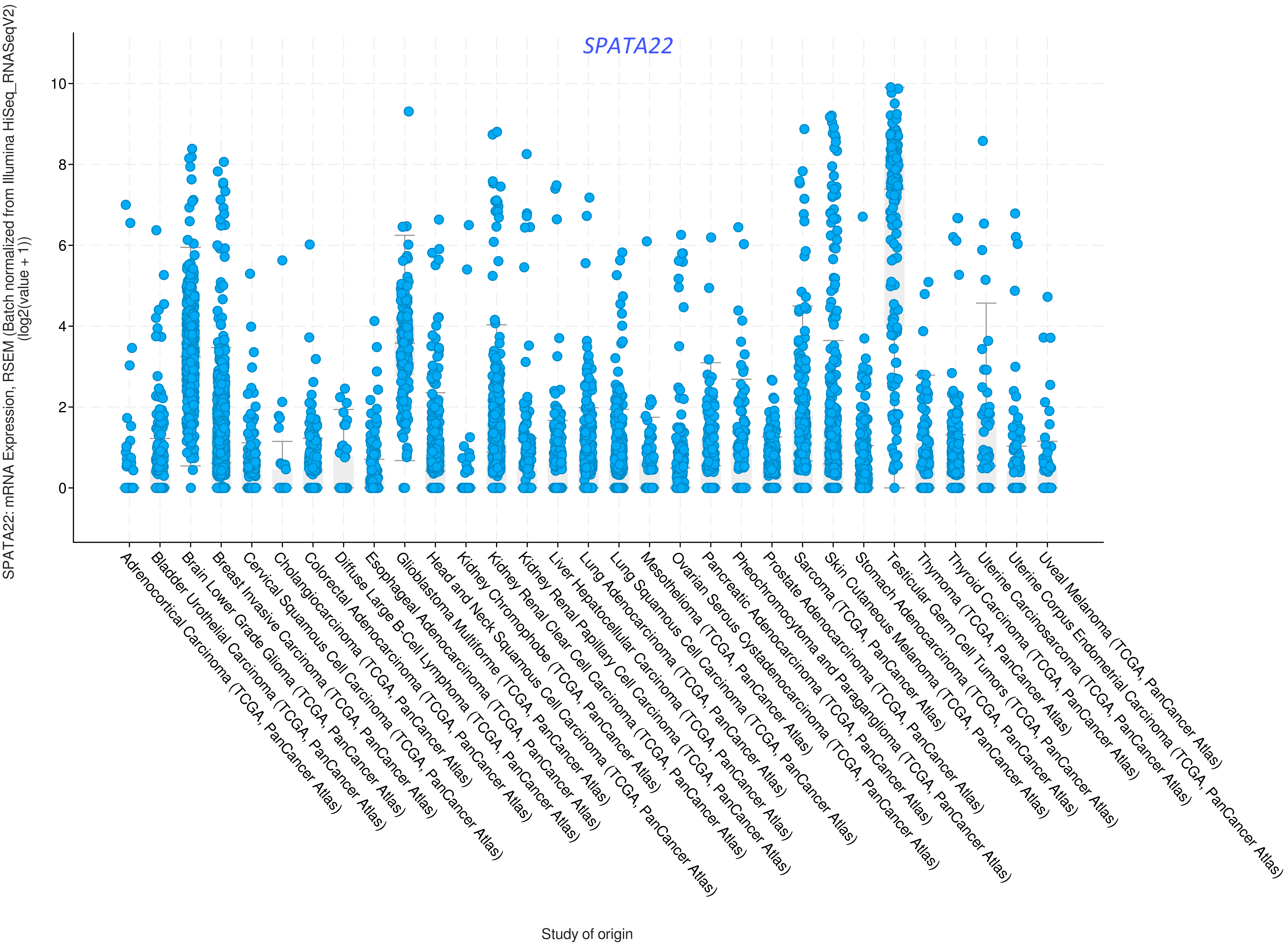

SPATA22
SPATA22: mRNA Expression, RSEM (Batch normalized from Illumina HiSeq_RNASeqV2) (log2(value + 1))
0
2
4
6
8
10
Study of origin
Adrenocortical Carcinoma (TCGA, PanCancer Atlas)
Bladder Urothelial Carcinoma (TCGA, PanCancer Atlas)
Brain Lower Grade Glioma (TCGA, PanCancer Atlas)
Breast Invasive Carcinoma (TCGA, PanCancer Atlas)
Cervical Squamous Cell Carcinoma (TCGA, PanCancer Atlas)
Cholangiocarcinoma (TCGA, PanCancer Atlas)
Colorectal Adenocarcinoma (TCGA, PanCancer Atlas)
Diffuse Large B-Cell Lymphoma (TCGA, PanCancer Atlas)
Esophageal Adenocarcinoma (TCGA, PanCancer Atlas)
Glioblastoma Multiforme (TCGA, PanCancer Atlas)
Head and Neck Squamous Cell Carcinoma (TCGA, PanCancer Atlas)
Kidney Chromophobe (TCGA, PanCancer Atlas)
Kidney Renal Clear Cell Carcinoma (TCGA, PanCancer Atlas)
Kidney Renal Papillary Cell Carcinoma (TCGA, PanCancer Atlas)
Liver Hepatocellular Carcinoma (TCGA, PanCancer Atlas)
Lung Adenocarcinoma (TCGA, PanCancer Atlas)
Lung Squamous Cell Carcinoma (TCGA, PanCancer Atlas)
Mesothelioma (TCGA, PanCancer Atlas)
Ovarian Serous Cystadenocarcinoma (TCGA, PanCancer Atlas)
Pancreatic Adenocarcinoma (TCGA, PanCancer Atlas)
Pheochromocytoma and Paraganglioma (TCGA, PanCancer Atlas)
Prostate Adenocarcinoma (TCGA, PanCancer Atlas)
Sarcoma (TCGA, PanCancer Atlas)
Skin Cutaneous Melanoma (TCGA, PanCancer Atlas)
Stomach Adenocarcinoma (TCGA, PanCancer Atlas)
Testicular Germ Cell Tumors (TCGA, PanCancer Atlas)
Thymoma (TCGA, PanCancer Atlas)
Thyroid Carcinoma (TCGA, PanCancer Atlas)
Uterine Carcinosarcoma (TCGA, PanCancer Atlas)
Uterine Corpus Endometrial Carcinoma (TCGA, PanCancer Atlas)
Uveal Melanoma (TCGA, PanCancer Atlas)

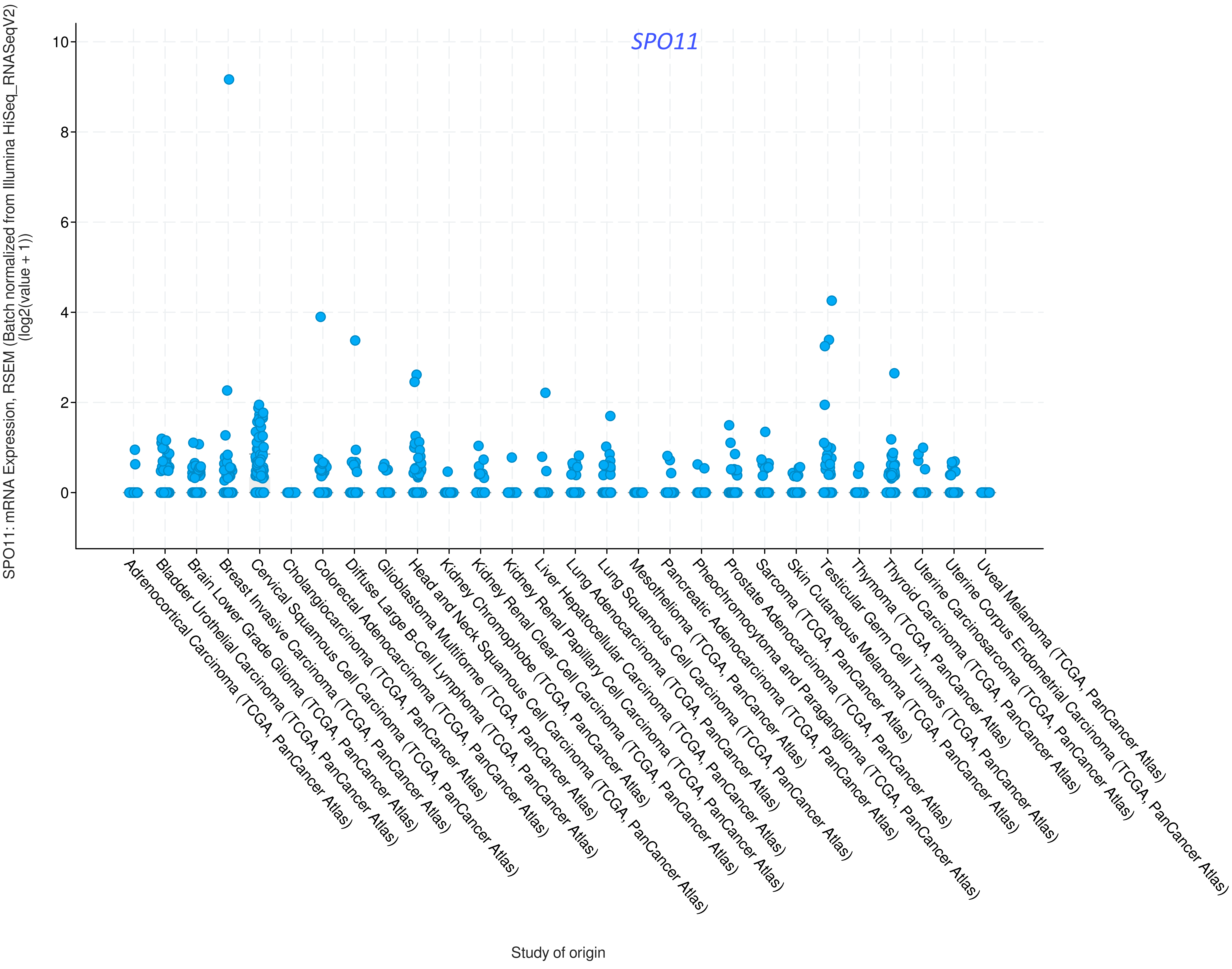
SPO11
SPO11: mRNA Expression, RSEM (Batch normalized from Illumina HiSeq_RNASeqV2) (log2(value + 1))
10
8
6
4
2
0
Adrenocortical Carcinoma (TCGA, PanCancer Atlas)
Bladder Urothelial Carcinoma (TCGA, PanCancer Atlas)
Brain Lower Grade Glioma (TCGA, PanCancer Atlas)
Breast Invasive Carcinoma (TCGA, PanCancer Atlas)
Cervical Squamous Cell Carcinoma (TCGA, PanCancer Atlas)
Cholangiocarcinoma (TCGA, PanCancer Atlas)
Colorectal Adenocarcinoma (TCGA, PanCancer Atlas)
Diffuse Large B-Cell Lymphoma (TCGA, PanCancer Atlas)
Glioblastoma Multiforme (TCGA, PanCancer Atlas)
Head and Neck Squamous Cell Carcinoma (TCGA, PanCancer Atlas)
Kidney Chromophobe (TCGA, PanCancer Atlas)
Kidney Renal Clear Cell Carcinoma (TCGA, PanCancer Atlas)
Kidney Renal Papillary Cell Carcinoma (TCGA, PanCancer Atlas)
Liver Hepatocellular Carcinoma (TCGA, PanCancer Atlas)
Lung Adenocarcinoma (TCGA, PanCancer Atlas)
Lung Squamous Cell Carcinoma (TCGA, PanCancer Atlas)
Mesothelioma (TCGA, PanCancer Atlas)
Pancreatic Adenocarcinoma (TCGA, PanCancer Atlas)
Pheochromocytoma and Paraganglioma (TCGA, PanCancer Atlas)
Prostate Adenocarcinoma (TCGA, PanCancer Atlas)
Sarcoma (TCGA, PanCancer Atlas)
Skin Cutaneous Melanoma (TCGA, PanCancer Atlas)
Testicular Germ Cell Tumors (TCGA, PanCancer Atlas)
Thymoma (TCGA, PanCancer Atlas)
Thyroid Carcinoma (TCGA, PanCancer Atlas)
Uterine Carcinosarcoma (TCGA, PanCancer Atlas)
Uterine Corpus Endometrial Carcinoma (TCGA, PanCancer Atlas)
Uveal Melanoma (TCGA, PanCancer Atlas)
Study of origin

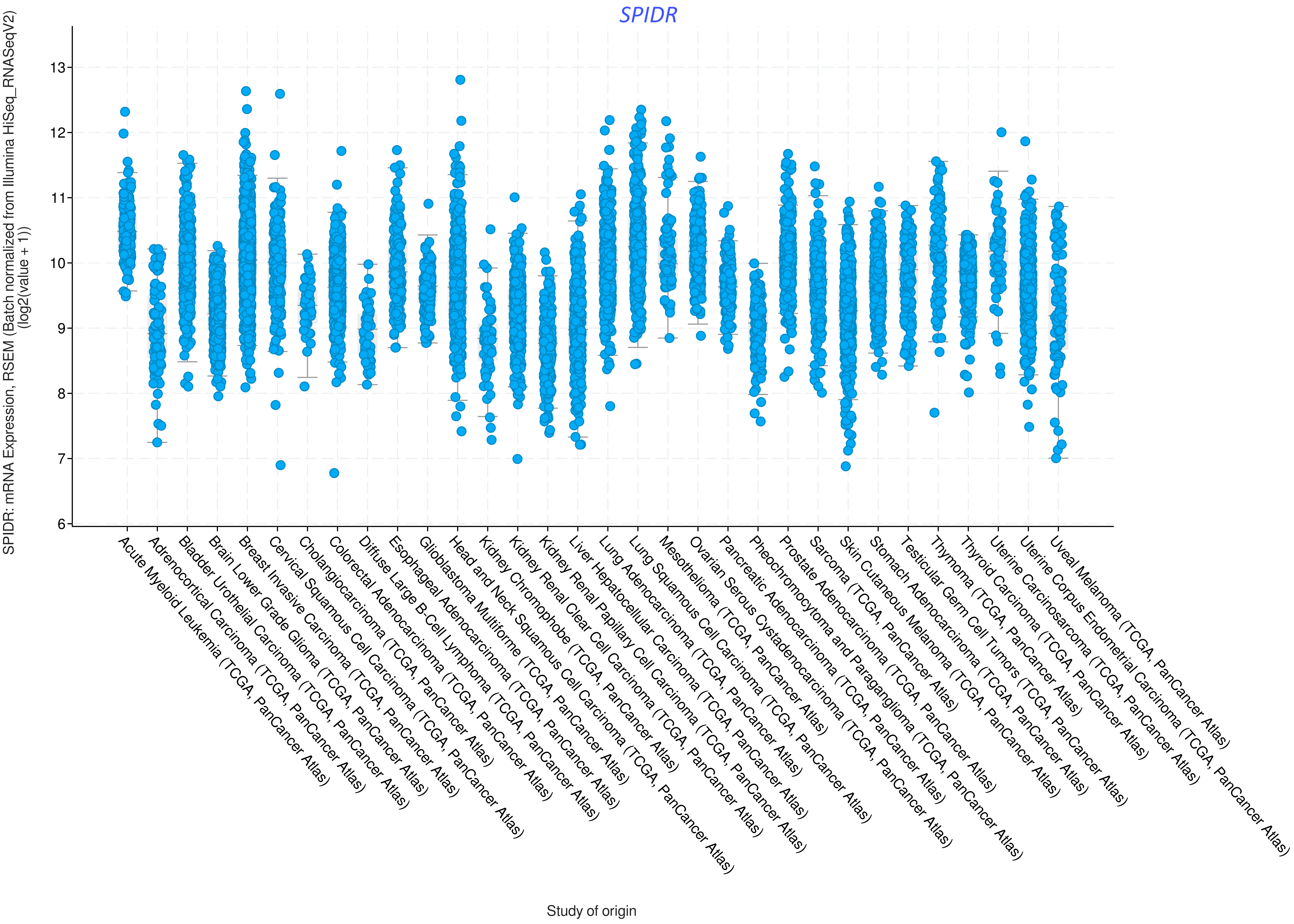
SPIDR
SPIDR: mRNA Expression, RSEM (Batch normalized from Illumina HiSeq_RNASeqV2) (log2(value + 1))
13
12
11
10
9
8
7
6
Acute Myeloid Leukemia (TCGA, PanCancer Atlas)
Adrenocortical Carcinoma (TCGA, PanCancer Atlas)
Bladder Urothelial Carcinoma (TCGA, PanCancer Atlas)
Brain Lower Grade Glioma (TCGA, PanCancer Atlas)
Breast Invasive Carcinoma (TCGA, PanCancer Atlas)
Cervical Squamous Cell Carcinoma (TCGA, PanCancer Atlas)
Cholangiocarcinoma (TCGA, PanCancer Atlas)
Colorectal Adenocarcinoma (TCGA, PanCancer Atlas)
Diffuse Large B-Cell Lymphoma (TCGA, PanCancer Atlas)
Esophageal Adenocarcinoma (TCGA, PanCancer Atlas)
Glioblastoma Multiforme (TCGA, PanCancer Atlas)
Head and Neck Squamous Cell Carcinoma (TCGA, PanCancer Atlas)
Kidney Chromophobe (TCGA, PanCancer Atlas)
Kidney Renal Clear Cell Carcinoma (TCGA, PanCancer Atlas)
Kidney Renal Papillary Cell Carcinoma (TCGA, PanCancer Atlas)
Liver Hepatocellular Carcinoma (TCGA, PanCancer Atlas)
Lung Adenocarcinoma (TCGA, PanCancer Atlas)
Lung Squamous Cell Carcinoma (TCGA, PanCancer Atlas)
Mesothelioma (TCGA, PanCancer Atlas)
Ovarian Serous Cystadenocarcinoma (TCGA, PanCancer Atlas)
Pancreatic Adenocarcinoma (TCGA, PanCancer Atlas)
Pheochromocytoma and Paraganglioma (TCGA, PanCancer Atlas)
Prostate Adenocarcinoma (TCGA, PanCancer Atlas)
Sarcoma (TCGA, PanCancer Atlas)
Skin Cutaneous Melanoma (TCGA, PanCancer Atlas)
Stomach Adenocarcinoma (TCGA, PanCancer Atlas)
Testicular Germ Cell Tumors (TCGA, PanCancer Atlas)
Thymoma (TCGA, PanCancer Atlas)
Thyroid Carcinoma (TCGA, PanCancer Atlas)
Uterine Carcinosarcoma (TCGA, PanCancer Atlas)
Uterine Corpus Endometrial Carcinoma (TCGA, PanCancer Atlas)
Uveal Melanoma (TCGA, PanCancer Atlas)
Study of origin

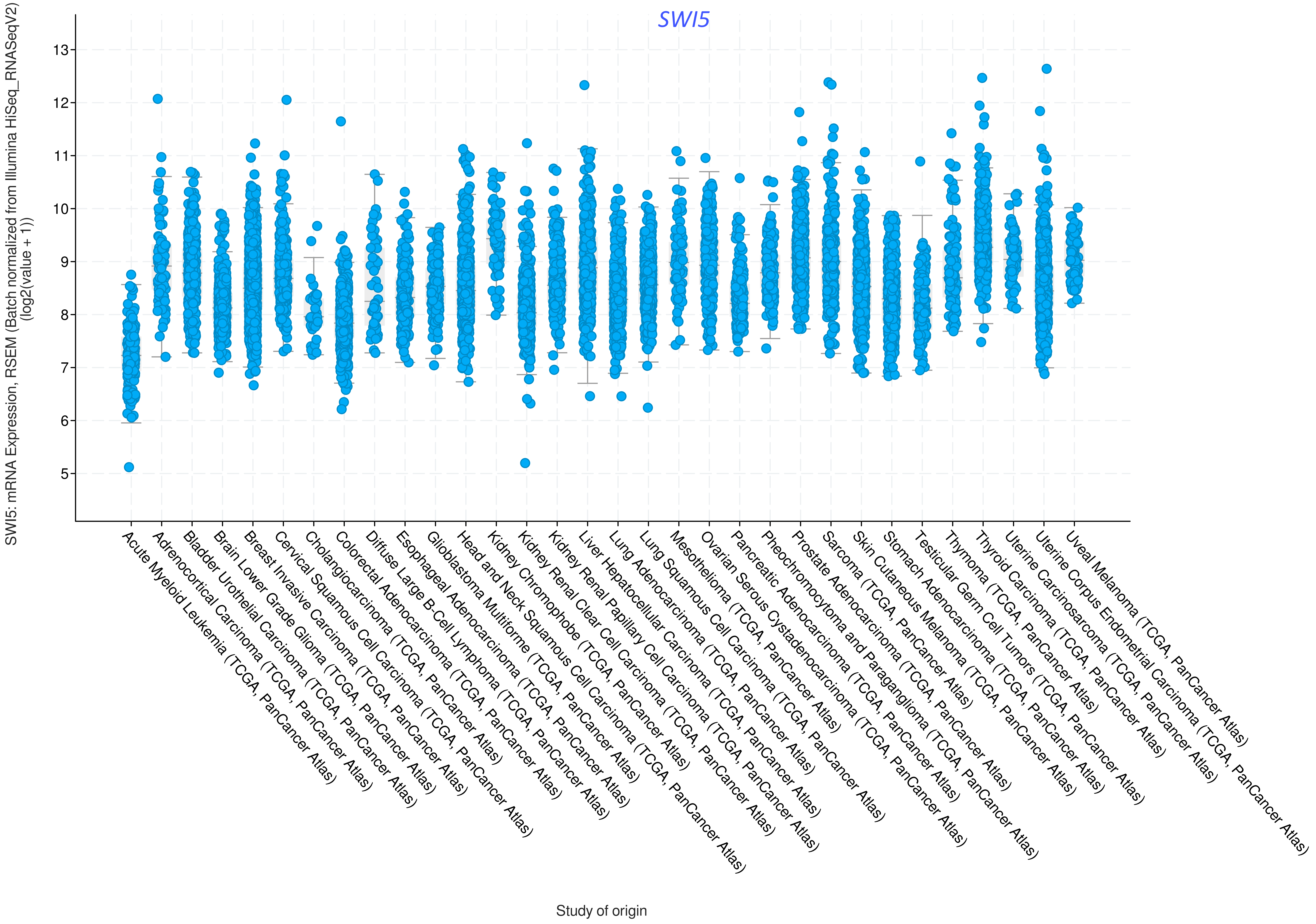
SWI5
SWI5: mRNA Expression, RSEM (Batch normalized from Illumina HiSeq_RNASeqV2) (log2(value + 1))
13
12
11
10
9
8
7
6
5
Acute Myeloid Leukemia (TCGA, PanCancer Atlas)
Adrenocortical Carcinoma (TCGA, PanCancer Atlas)
Bladder Urothelial Carcinoma (TCGA, PanCancer Atlas)
Brain Lower Grade Glioma (TCGA, PanCancer Atlas)
Breast Invasive Carcinoma (TCGA, PanCancer Atlas)
Cervical Squamous Cell Carcinoma (TCGA, PanCancer Atlas)
Cholangiocarcinoma (TCGA, PanCancer Atlas)
Colorectal Adenocarcinoma (TCGA, PanCancer Atlas)
Diffuse Large B-Cell Lymphoma (TCGA, PanCancer Atlas)
Esophageal Adenocarcinoma (TCGA, PanCancer Atlas)
Glioblastoma Multiforme (TCGA, PanCancer Atlas)
Head and Neck Squamous Cell Carcinoma (TCGA, PanCancer Atlas)
Kidney Chromophobe (TCGA, PanCancer Atlas)
Kidney Renal Clear Cell Carcinoma (TCGA, PanCancer Atlas)
Kidney Renal Papillary Cell Carcinoma (TCGA, PanCancer Atlas)
Liver Hepatocellular Carcinoma (TCGA, PanCancer Atlas)
Lung Adenocarcinoma (TCGA, PanCancer Atlas)
Lung Squamous Cell Carcinoma (TCGA, PanCancer Atlas)
Mesothelioma (TCGA, PanCancer Atlas)
Ovarian Serous Cystadenocarcinoma (TCGA, PanCancer Atlas)
Pancreatic Adenocarcinoma (TCGA, PanCancer Atlas)
Pheochromocytoma and Paraganglioma (TCGA, PanCancer Atlas)
Prostate Adenocarcinoma (TCGA, PanCancer Atlas)
Sarcoma (TCGA, PanCancer Atlas)
Skin Cutaneous Melanoma (TCGA, PanCancer Atlas)
Stomach Adenocarcinoma (TCGA, PanCancer Atlas)
Testicular Germ Cell Tumors (TCGA, PanCancer Atlas)
Thymoma (TCGA, PanCancer Atlas)
Thyroid Carcinoma (TCGA, PanCancer Atlas)
Uterine Carcinosarcoma (TCGA, PanCancer Atlas)
Uterine Corpus Endometrial Carcinoma (TCGA, PanCancer Atlas)
Uveal Melanoma (TCGA, PanCancer Atlas)
Study of origin

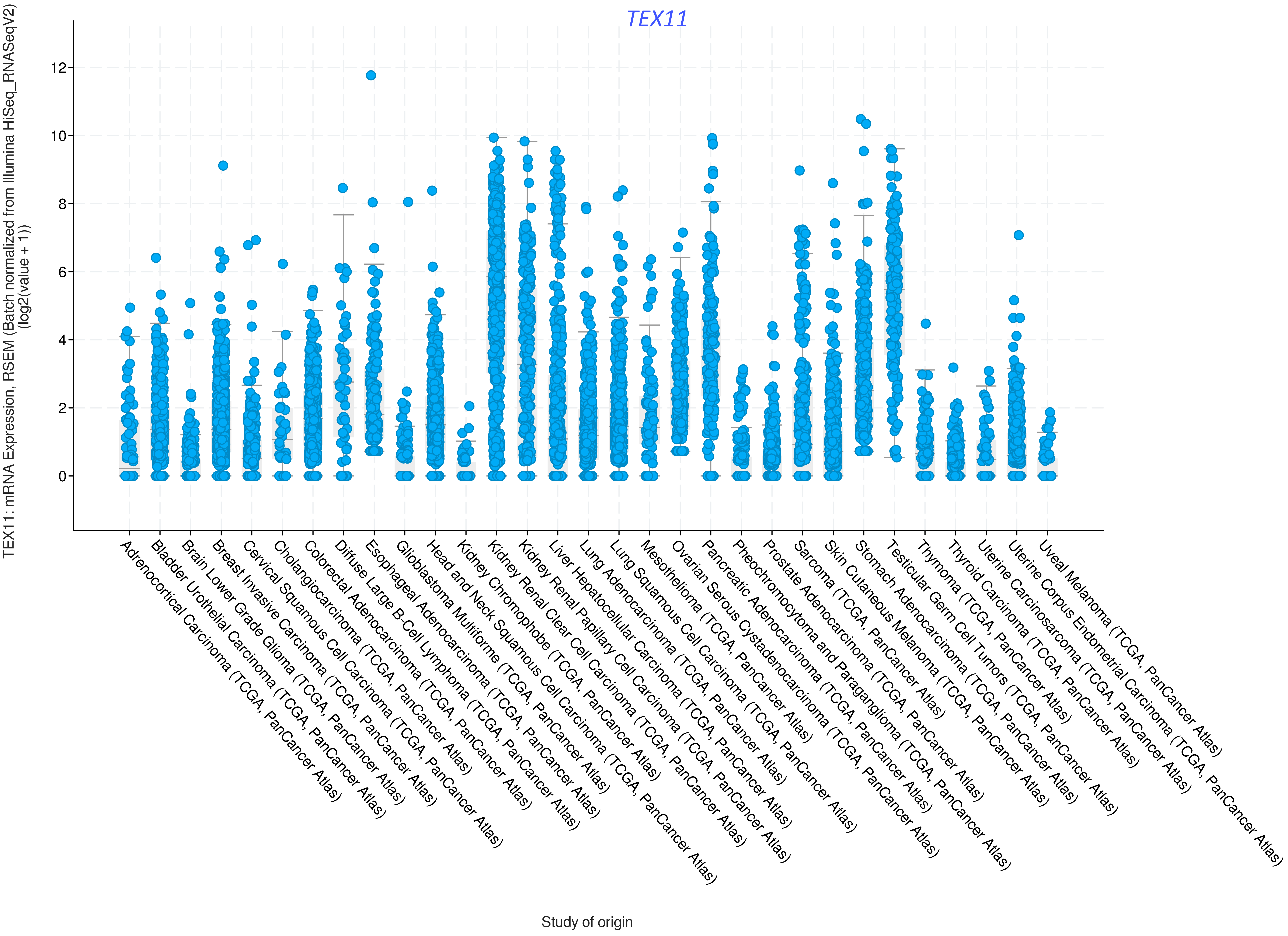
TEX11
TEX11: mRNA Expression, RSEM (Batch normalized from Illumina HiSeq_RNASeqV2) (log2(value + 1))
12
10
8
6
4
2
0
Study of origin
Adrenocortical Carcinoma (TCGA, PanCancer Atlas)
Bladder Urothelial Carcinoma (TCGA, PanCancer Atlas)
Brain Lower Grade Glioma (TCGA, PanCancer Atlas)
Breast Invasive Carcinoma (TCGA, PanCancer Atlas)
Cervical Squamous Cell Carcinoma (TCGA, PanCancer Atlas)
Cholangiocarcinoma (TCGA, PanCancer Atlas)
Colorectal Adenocarcinoma (TCGA, PanCancer Atlas)
Diffuse Large B-Cell Lymphoma (TCGA, PanCancer Atlas)
Esophageal Adenocarcinoma (TCGA, PanCancer Atlas)
Glioblastoma Multiforme (TCGA, PanCancer Atlas)
Head and Neck Squamous Cell Carcinoma (TCGA, PanCancer Atlas)
Kidney Chromophobe (TCGA, PanCancer Atlas)
Kidney Renal Clear Cell Carcinoma (TCGA, PanCancer Atlas)
Kidney Renal Papillary Cell Carcinoma (TCGA, PanCancer Atlas)
Liver Hepatocellular Carcinoma (TCGA, PanCancer Atlas)
Lung Adenocarcinoma (TCGA, PanCancer Atlas)
Lung Squamous Cell Carcinoma (TCGA, PanCancer Atlas)
Mesothelioma (TCGA, PanCancer Atlas)
Ovarian Serous Cystadenocarcinoma (TCGA, PanCancer Atlas)
Pancreatic Adenocarcinoma (TCGA, PanCancer Atlas)
Pheochromocytoma and Paraganglioma (TCGA, PanCancer Atlas)
Prostate Adenocarcinoma (TCGA, PanCancer Atlas)
Sarcoma (TCGA, PanCancer Atlas)
Skin Cutaneous Melanoma (TCGA, PanCancer Atlas)
Stomach Adenocarcinoma (TCGA, PanCancer Atlas)
Testicular Germ Cell Tumors (TCGA, PanCancer Atlas)
Thymoma (TCGA, PanCancer Atlas)
Thyroid Carcinoma (TCGA, PanCancer Atlas)
Uterine Carcinosarcoma (TCGA, PanCancer Atlas)
Uterine Corpus Endometrial Carcinoma (TCGA, PanCancer Atlas)
Uveal Melanoma (TCGA, PanCancer Atlas)

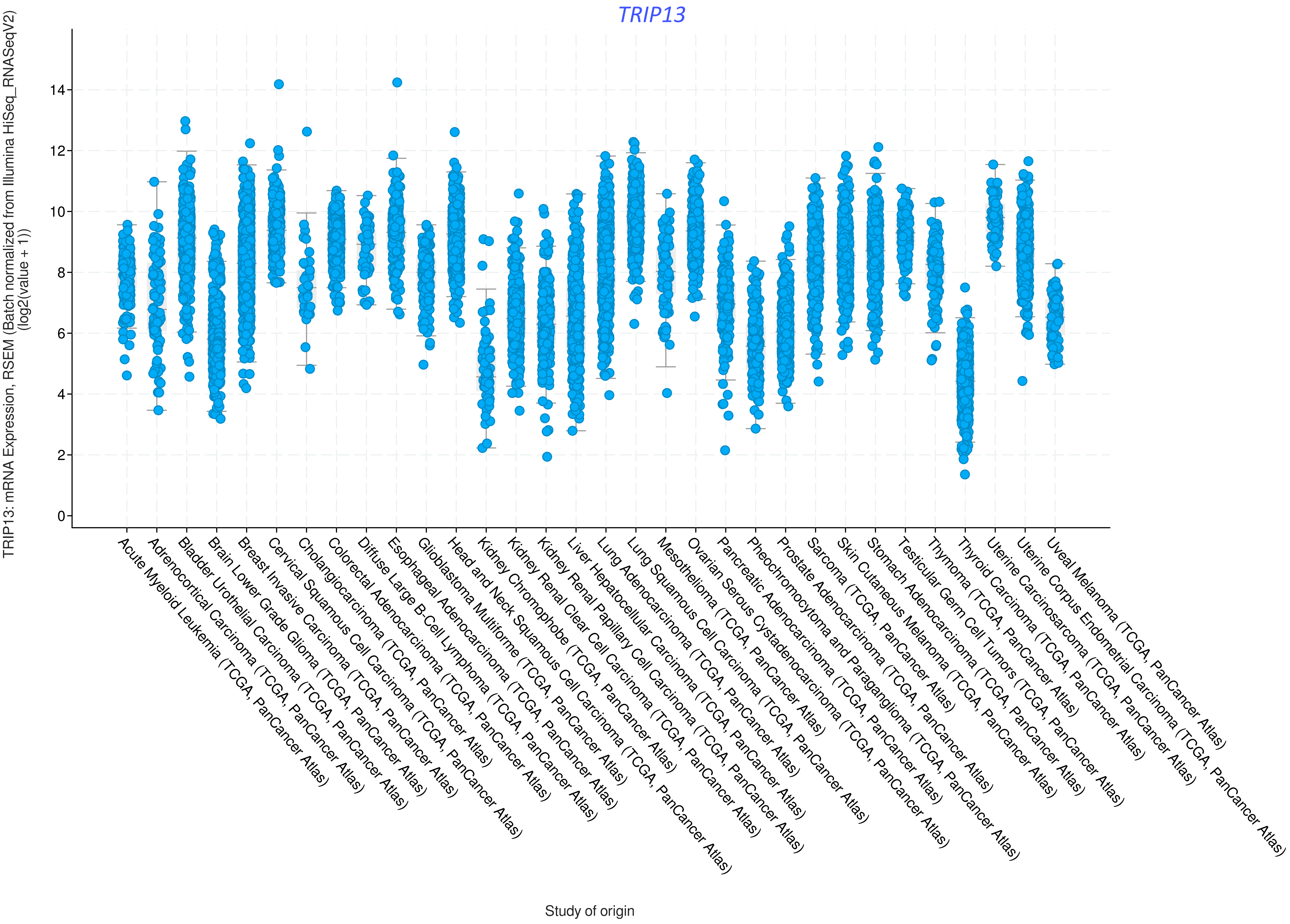
TRIP13
TRIP13: mRNA Expression, RSEM (Batch normalized from Illumina HiSeq_RNASeqV2) (log2(value + 1))
0
2
4
6
8
10
12
14
Study of origin
Acute Myeloid Leukemia (TCGA, PanCancer Atlas)
Adrenocortical Carcinoma (TCGA, PanCancer Atlas)
Bladder Urothelial Carcinoma (TCGA, PanCancer Atlas)
Brain Lower Grade Glioma (TCGA, PanCancer Atlas)
Breast Invasive Carcinoma (TCGA, PanCancer Atlas)
Cervical Squamous Cell Carcinoma (TCGA, PanCancer Atlas)
Cholangiocarcinoma (TCGA, PanCancer Atlas)
Colorectal Adenocarcinoma (TCGA, PanCancer Atlas)
Diffuse Large B-Cell Lymphoma (TCGA, PanCancer Atlas)
Esophageal Adenocarcinoma (TCGA, PanCancer Atlas)
Glioblastoma Multiforme (TCGA, PanCancer Atlas)
Head and Neck Squamous Cell Carcinoma (TCGA, PanCancer Atlas)
Kidney Chromophobe (TCGA, PanCancer Atlas)
Kidney Renal Clear Cell Carcinoma (TCGA, PanCancer Atlas)
Kidney Renal Papillary Cell Carcinoma (TCGA, PanCancer Atlas)
Liver Hepatocellular Carcinoma (TCGA, PanCancer Atlas)
Lung Adenocarcinoma (TCGA, PanCancer Atlas)
Lung Squamous Cell Carcinoma (TCGA, PanCancer Atlas)
Mesothelioma (TCGA, PanCancer Atlas)
Ovarian Serous Cystadenocarcinoma (TCGA, PanCancer Atlas)
Pancreatic Adenocarcinoma (TCGA, PanCancer Atlas)
Pheochromocytoma and Paraganglioma (TCGA, PanCancer Atlas)
Prostate Adenocarcinoma (TCGA, PanCancer Atlas)
Sarcoma (TCGA, PanCancer Atlas)
Skin Cutaneous Melanoma (TCGA, PanCancer Atlas)
Stomach Adenocarcinoma (TCGA, PanCancer Atlas)
Testicular Germ Cell Tumors (TCGA, PanCancer Atlas)
Thymoma (TCGA, PanCancer Atlas)
Thyroid Carcinoma (TCGA, PanCancer Atlas)
Uterine Carcinosarcoma (TCGA, PanCancer Atlas)
Uterine Corpus Endometrial Carcinoma (TCGA, PanCancer Atlas)
Uveal Melanoma (TCGA, PanCancer Atlas)

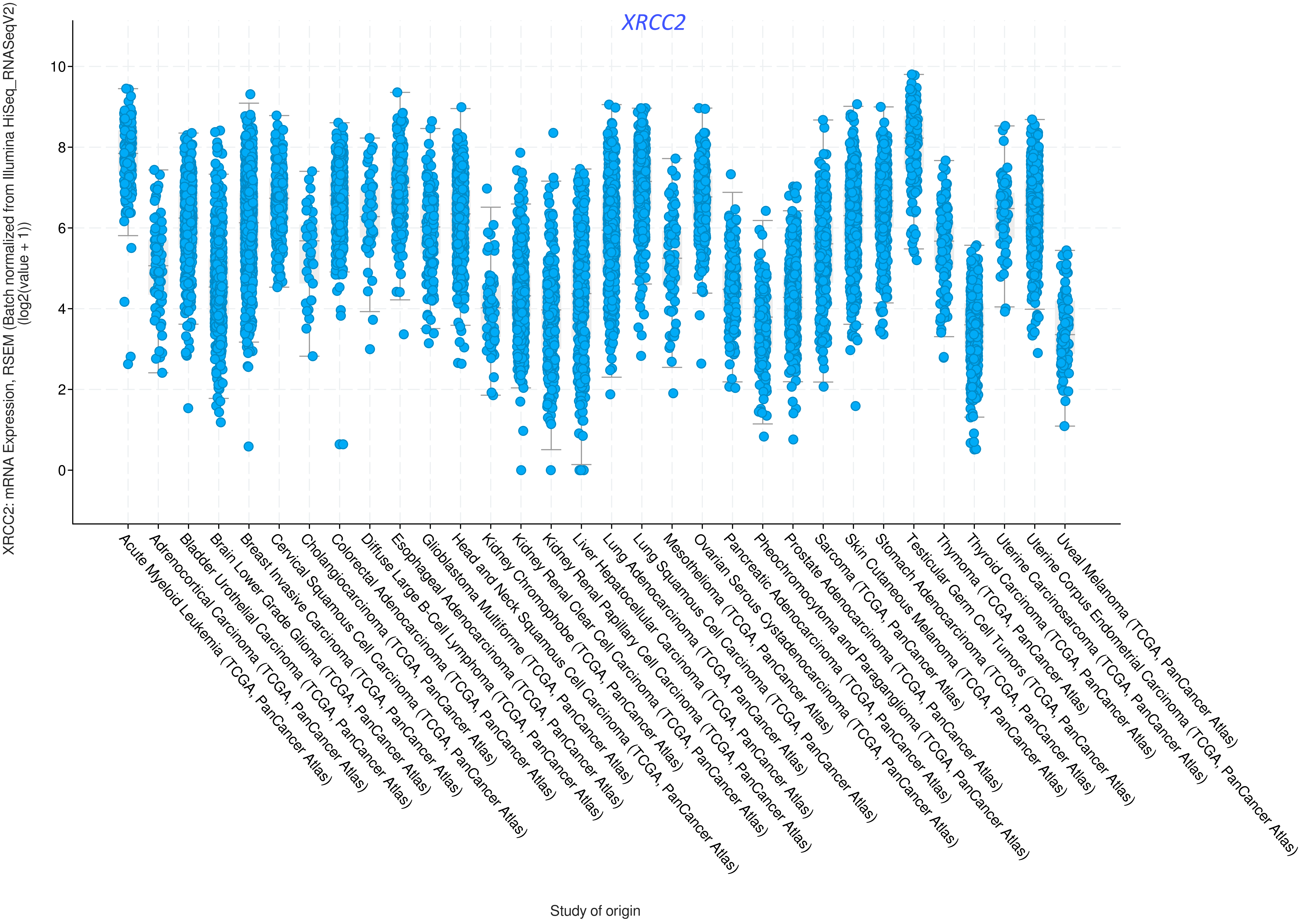

XRCC2
XRCC2: mRNA Expression, RSEM (Batch normalized from Illumina HiSeq_RNASeqV2) (log2(value + 1))
10
8
6
4
2
0
Acute Myeloid Leukemia (TCGA, PanCancer Atlas)
Adrenocortical Carcinoma (TCGA, PanCancer Atlas)
Bladder Urothelial Carcinoma (TCGA, PanCancer Atlas)
Brain Lower Grade Glioma (TCGA, PanCancer Atlas)
Breast Invasive Carcinoma (TCGA, PanCancer Atlas)
Cervical Squamous Cell Carcinoma (TCGA, PanCancer Atlas)
Cholangiocarcinoma (TCGA, PanCancer Atlas)
Colorectal Adenocarcinoma (TCGA, PanCancer Atlas)
Diffuse Large B-Cell Lymphoma (TCGA, PanCancer Atlas)
Esophageal Adenocarcinoma (TCGA, PanCancer Atlas)
Glioblastoma Multiforme (TCGA, PanCancer Atlas)
Head and Neck Squamous Cell Carcinoma (TCGA, PanCancer Atlas)
Kidney Chromophobe (TCGA, PanCancer Atlas)
Kidney Renal Clear Cell Carcinoma (TCGA, PanCancer Atlas)
Kidney Renal Papillary Cell Carcinoma (TCGA, PanCancer Atlas)
Liver Hepatocellular Carcinoma (TCGA, PanCancer Atlas)
Lung Adenocarcinoma (TCGA, PanCancer Atlas)
Lung Squamous Cell Carcinoma (TCGA, PanCancer Atlas)
Mesothelioma (TCGA, PanCancer Atlas)
Ovarian Serous Cystadenocarcinoma (TCGA, PanCancer Atlas)
Pancreatic Adenocarcinoma (TCGA, PanCancer Atlas)
Pheochromocytoma and Paraganglioma (TCGA, PanCancer Atlas)
Prostate Adenocarcinoma (TCGA, PanCancer Atlas)
Sarcoma (TCGA, PanCancer Atlas)
Skin Cutaneous Melanoma (TCGA, PanCancer Atlas)
Stomach Adenocarcinoma (TCGA, PanCancer Atlas)
Testicular Germ Cell Tumors (TCGA, PanCancer Atlas)
Thymoma (TCGA, PanCancer Atlas)
Thyroid Carcinoma (TCGA, PanCancer Atlas)
Uterine Carcinosarcoma (TCGA, PanCancer Atlas)
Uterine Corpus Endometrial Carcinoma (TCGA, PanCancer Atlas)
Uveal Melanoma (TCGA, PanCancer Atlas)
Study of origin

Table S1. Additional information of genes mentioned in the review

Transcriptomic analysis of each gene in human tissues, cell lines, and cancer was performed using data obtained from the Protein Atlas portal (https://www.proteinatlas.org/). The genes were categorized based on their involvement in the structural axes of the chromosome, structural components of the synaptonemal complex, or the recombination machinery. Within each category, the genes were listed alphabetically. Furthermore, the expression of each gene was extracted from the TCGA Pan Cancer Atlas, available at the cBioPortal (https://www.cbioportal.org/). The expression data is presented separately in Figure S1. The disease-gene associations described are derived from automatic text mining of the biomedical literature, manually curated database annotations, cancer mutation data, and genome-wide association studies or by knowledge (https://diseases.jensenlab.org). Only those with high confidence of each association are indicated.

Table S1: List of genes mentioned in the review

	Tissue	Cell line	Cancer	Mice KO	Text mining	Knowledge
**Structural axes**						
**RAD21L1**	Tissue enriched (testis)<br>Detected in single	Cancer enhanced (Bladder and ovarian cancer)	Not detected<br>Not detected	male infertility, female subfertilty (POI) [1]	Male infertility<br>Infertility<br>Primary ovarian insufficiency<br>Cornelia de Lange syndrome	
**REC8**	Tissue enhanced (pituitary gland)<br>Detected in many	end	Low cancer specificity (endometrial and ovarian)<br>Detected in all	male and female infertility [2]	Infertility<br>Primary ovarian insufficiency<br>Male infertility<br>Cornelia de Lange syndrome	
**SMC1B**	Tissue enriched (testis)<br>Detected in single	Cancer enhanced (Cervical cancer)<br>Detected in many	Cancer enriched (cervical cancer with high expression)<br>Detected in many	male and female infertility [3]	Primary ovarian insufficiency<br>Infertility<br>Cornelia de Lange syndrome<br>Male infertility<br>Roberts syndrome	
**STAG3**	Tissue enriched (testis)<br>Detected in single	Cancer enhanced (Bile duct cancer)<br>Detected in some	Low cancer specificity<br>Detected in many	male and female infertility [4,5]	Primary ovarian insufficiency<br>Infertility<br>Male infertility<br>Amenorrhea<br>Gonadal dysgenesis<br>Premature menopause	Primary ovarian insufficiency, azoospermia
**HORMAD1**	Tissue enriched (testis)<br>Detected in some	Cancer enhanced (Gallbladder cancer)<br>Detected in many	Cancer enhanced (testis cancer)<br>Detected in many	male and female infertility [6]	Male infertility<br>Infertility	
**HORMAD2**	Tissue enriched (testis)<br>Detected in some	Very low detected	Very low detected (liver)	male and female infertility [7]	IgA glomerulonephritis	
**structural SC**						
**C14ORF39/SIX6OS1**	Tissue enhanced (pituitary gland, salivary gland, testis)<br>Detected in some	Cancer enhanced (Bone cancer, Neuroblastoma)<br>Detected in some	Cancer enhanced (testis cancer)<br>Detected in single	male and female infertility [8]	Primary ovarian insufficiency<br>Azoospermia<br>Infertility	Primary ovarian insufficiency
**SYCE1**	Tissue enriched (testis)<br>Detected in many	Cancer enhanced (Bile duct cancer)<br>Detected in many	Low detected (testis)	Male and female infertility [9]	Primary ovarian insufficiency, Male infertility, Infertility, Amenorrhea, Gonadal dysgenesis<br>Premature menopause	Primary ovarian insufficiency
**SYCE2**	Tissue enriched (testis)<br>Detected in some	Low cancer specificity<br>Detected in many	Low cancer specificity, (cervical higher)<br>Detected in many	Male and female infertility [10]	Infertility<br>Male infertility	
**SYCE3**	Tissue enriched (testis)<br>Detected in many	Cancer enriched (Prostate cancer)<br>Detected in many	Low cancer specificity<br>Detected in many	Male and female infertility [11]	Infertility, Male infertility, Primary ovarian insufficiency	

**SYCP1**	Tissue enriched (testis)<br>Detected in single	Cancer enriched (Bone cancer)<br>Detected in single	Very low detected (breast)	male and female infertility [12]	Infertility<br>Male infertility<br>Primary ovarian insufficiency	Primary ovarian insufficiency
**SYCP2**	Tissue enhanced (testis)<br>Detected in many	Cancer enhanced (Breast cancer)<br>Detected in many	Group enriched (breast cancer, cervical cancer)<br>Detected in many	male and female infertility [13]	Male infertility<br>Infertility, Primary ovarian insufficiency	
**SYCP3**	Testis - Spermatogenesis (mainly)	Cancer enriched (Myeloma)Detected in single	Cancer enriched (testis cancer)<br>Detected in single	Male infertility, female subfertility [14]	Male infertility<br>Infertility<br>Primary ovarian insufficiency<br>Klinefelter syndrome	
**TEX12**	Group enriched (retina, testis)<br>Detected in some	Not detected<br>Not detected	very low detected	Male and female infertility [15]	Infertility, male infertility	
RECOMBINATION						
**ANKRD31**	Tissue enriched (testis)<br>Detected in single	Few cell lines, very low expresion	very low expression (breast)	Female POI, male infertile [16]		
**BLM**	Tissue enhanced (bone marrow, lymphoid tissue, salivary gland)<br>Detected in some	Low cancer specificity<br>Detected in all	Low cancer specificity<br>Detected in many	cKO, only males analyzed, meiotic arrest [17]	Bloom syndrome<br>Werner syndrome<br>Rothmund-Thomson syndrome<br>Rapadilino syndrome<br>Xeroderma pigmentosum<br>Anemia	Bloom syndrome
**BRCA2**	Tissue enhanced (bone marrow, lymphoid tissue, testis)<br>Detected in some	Low cancer specificity<br>Detected in all	Low cancer specificity<br>Detected in many		Breast cancer<br>Ovarian cancer<br>Hereditary breast ovarian cancer syndrome<br>Carcinoma<br>Lynch syndrome<br>Prostate cancer<br>Anemia<br>Pancreatic cancer<br>Serous cystadenocarcinoma<br>Li-Fraumeni syndrome	Breast cancer<br>Fanconi anemia<br>Ovarian cancer<br>Cholangiocarcinoma<br>Prostate cancer<br>Fanconi anemia<br>Cancer
**BRME1/C19orf57**	Tissue enhanced (testis)<br>Detected in many	Low cancer specificity<br>Detected in many	Low cancer specificity<br>Detected in many	male and female infertility [18]		
**C11orf80 (TOP6BL)**	Tissue enhanced (skin)<br>Detected in all	Low cancer specificity<br>Detected in all	Low cancer specificity<br>Detected in all	male and female infertility [19]	Gestational trophoblastic neoplasm	
**CCNB1IP1/HEI10**	Low tissue specificity<br>Detected in all	Low cancer specificity<br>Detected in all	Low cancer specificity<br>Detected in all	Male and female infertility [20]		
**DMC1**	Tissue enhanced (testis)<br>Detected in many	Low cancer specificity<br>Detected in many	Cancer enhanced (testis cancer)<br>Detected in some	Male and female infertility [21]	Azoosperima	

**EXO1**	Tissue enhanced (bone marrow,testis)<br>Detected in some	Low cancer specificity<br>Detected in all	Low cancer specificity<br>Detected in many (testis)	null are infertile and enzymatic activity dead are fertile [22]	Xeroderma pigmentosum group G<br>Cancer<br>Werner syndrome<br>Aicardi-Goutieres syndrome<br>Breast disease<br>Gastrointestinal system disease<br>Anemia	
**FANCA**	Tissue enhanced (bone marrow, testis)<br>Detected in many	Low cancer specificity<br>Detected in all	Low cancer specificity<br>Detected in many	male and female subfertility [23]	Anemia<br>Cancer<br>Breast disease<br>Xeroderma pigmentosum<br>Bloom syndrome<br>Hereditary breast ovarian cancer syndrome<br>Microcephaly<br>Prostate disease<br>Nijmegen breakage syndrome<br>Gastrointestinal system disease	Fanconi anemia, melanoma
**FANCM**	Low tissue specificity<br>Detected in many	Low cancer specificity<br>Detected in all	Low cancer specificity<br>Detected in many	male and female subfertility [23]	Anemia<br>Bloom syndrome<br>Ovarian cancer<br>Breast cancer<br>Hereditary breast ovarian cancer syndrome<br>Xeroderma pigmentosum<br>Ataxia telangiectasia<br>Azoospermia	Fanconi anemia, Primary ovarian insufficiency
**HFM1**	Tissue enhanced (pituitary gland, testis)<br>Detected in some	Cancer enhanced (Neuroblastoma)<br>Detected in some	Cancer enhanced (testis cancer)<br>Detected in single	male and female infertility [24]	Primary ovarian insufficiency<br>Infertility<br>Azoospermia<br>Amenorrhea	Primary ovarian insufficiency
**HELQ**	Low tissue specificity<br>Detected in all	Low cancer specificity<br>Detected in all	Low cancer specificity<br>Detected in all	male and female subfertility [25]	low POI, low Warsaw syndrome	
**HROB**	Tissue enhanced (bone marrow, testis)<br>Detected in some	Low cancer specificity<br>Detected in all	Low cancer specificity<br>Detected in many (testis and cervical)	male and female infertility [26]		
**HSF2BP**	Tissue enriched (testis)<br>Detected in some	Low cancer specificity<br>Detected in many	Low cancer specificity<br>Detected in many	male and female infertility [18]	POI, Infertility	Primary ovarian insufficiency
**HOP2/PSMC3IP**	Tissue enhanced (testis)<br>Detected in many	Low cancer specificity<br>Detected in all	Low cancer specificity<br>Detected in all	male and female infertility [27]	Primary ovarian insufficiency<br>Gonadal dysgenesis<br>Amenorrhea	

**IHO1**	Group enriched (choroid plexus, testis)<br>Detected in many	Cancer enhanced (Skin and bone cancer)	low detected, enhanced in melanoma	male and female infertility [28]	Gestational trophoblastic neoplasm<br>Infertility	
**MEI4**	Tissue enhanced (kidney, testis, thyroid gland)<br>Detected in some	low detected (lung)	Cancer enhanced (renal cancer, testis cancer)<br>Detected in some	male and female infertility [29]		
**MEI1**	Tissue enhanced (lymphoid tissue, testis)<br>Detected in man	Cancer enhanced (Myeloma)<br>Detected in some	Cancer enhanced (cervical cancer)<br>Detected in many	male and female infertility [30]	Gestational trophoblastic neoplasm<br>Male infertility<br>Infertility	
**MEIOB**	Tissue enriched (testis)<br>Detected in some	Cancer enhanced (Bone cancer, Rhabdoid)<br>Detected in many	Very low expression<br>dtected in testis cancer	male and female infertility [31]	Azoospermia<br>Primary ovarian insufficiency<br>Infertility sertoli cell-only syndrome	
**MCM8**	Low tissue specificity<br>Detected in all	Low cancer specificity<br>Detected in all	Low cancer specificity<br>Detected in all	Male and female infertility [32]	Primary ovarian insufficiency<br>Amenorrhea<br>Gonadal dysgenesis<br>Premature menopause<br>Infertility	Primary ovarian insufficiency
**MCM9**	Low tissue specificity<br>Detected in all	Low cancer specificity<br>Detected in all	Low cancer specificity<br>Detected in all	Subfertile males and infertile females [32]	Primary ovarian insufficiency<br>Amenorrhea<br>Gonadal dysgenesis	
**MND1**	Tissue enhanced (bone marrow, lymphoid tissue, testis)<br>Detected in many	Low cancer specificity<br>Detected in all	Low cancer specificity<br>Detected in all	Male and female infertility [27]		
**MLH1**	Low tissue specificity<br>Detected in all very high	Low cancer specificity<br>Detected in all	Low cancer specificity<br>Low cancer specificity<br>Detected in all very high	Male and female infertility [33]	Lynch syndrome<br>Mismatch repair cancer syndrome<br>Cancer<br>Gastrointestinal system disease<br>Endometriosis<br>Uterine disease<br>Adenoma<br>Ovarian disease<br>Breast disease<br>Hereditary breast ovarian cancer syndrome	Mismatch repair cancer syndrome<br>Ovarian cancer, Lynch Syndrome
**MLH3**	Low tissue specificity<br>Detected in all	Low tissue specificity<br>Detected in all	Low tissue specificity<br>Detected in all	Male and female are infertile [34]	Lynch syndrome<br>Mismatch repair cancer syndrome<br>Cancer<br>Gastrointestinal system disease<br>Endometriosis<br>Uterine disease	Lynch syndrome

**MSH4**	Tissue enhanced (brain, epididymis, testis)<br>Detected in some	Low expression (colorectal and lung caner)	very low or undetectable	Male and female infertility [35]	Lynch syndrome<br>Mismatch repair cancer syndrome<br>Cancer<br>Gastrointestinal system disease<br>Xeroderma pigmentosum group G<br>Endometriosis<br>Adenoma<br>Myotonic dystrophy type 1<br>Uterine disease<br>Ovarian disease	
**MSH5**	Tissue enhanced (testis)<br>Detected in many	Cancer enhanced (Gastric cancer)<br>Detected in some	Low cancer specificity<br>Detected in many	Male and female infertility [36]	Primary ovarian insufficiency<br>Common variable immunodeficiency<br>Immunoglobulin alpha deficiency	Primary ovarian insufficiency
**PRDM9**	Tissue enhanced (brain, epididymis, testis)<br>Detected in some	Cancer enhanced (Thyroid, myeloma cancer)<br>Detected in some	Not detected	male and female infertility [37]	Male infertility<br>Infertility<br>Charcot-Marie-Tooth disease type 1A<br>Hereditary neuropathy with liability to pressure palsies	
**PSMA8**	Tissue enriched (testis)<br>Detected in some	Group enriched (Gallbladder cancer, Lymphoma, Myeloma)<br>Detected in some	Not detected	male infertility [38]		
**RAD51**	Tissue enhanced (lymphoid tissue, testis)<br>Detected in many	Low cancer specificity<br>Detected in all	Cancer enhanced (testis cancer)<br>Detected in all	meiotic arrest in spemmatocytes by siRNA [39]	Cancer<br>Breast disease<br>Ovarian disease<br>Anemia<br>Xeroderma pigmentosum<br>Gastrointestinal system disease<br>Hereditary breast ovarian cancer syndrome<br>Bloom syndrome<br>Lung disease	Congenital mirror movement disorder<br>Breast cancer<br>Fanconi anemia

**RAD51B**	Low tissue specificity<br>Detected in all	Low cancer specificity<br>Detected in all	Low cancer specificity<br>Detected in many	Lethal [40]	Cancer<br>Breast disease<br>Ovarian disease<br>Restless legs syndrome<br>Uterine fibroid<br>Hereditary breast ovarian cancer syndrome<br>Anemia<br>Gastrointestinal system disease<br>Chronic obstructive pulmonary disease	
**RNF212**	Tissue enhanced (pancreas)<br>Detected in many	Group enriched (Cervical cancer, Gallbladder cancer, Lung cancer)<br>Detected in many	Cancer enhanced (cervical cancer, ovarian cancer)<br>Detected in many	male and female infertility [41]	Male infertility, Infertiity	Bone disease
**REC114**	Tissue enriched (testis)<br>Detected in some	few cell lines (kindey and lung)	Cancer enriched (testis cancer)	male and female infertility [42]	Gestational trophoblastic neoplasm<br>Infertility	
**RPA1**	Low tissue specificity<br>Detected in all	Low cancer specificity<br>Detected in all	Low cancer specificity<br>Detected in all	male infertile [43]	Xeroderma pigmentosum<br>Cancer	
**SPATA22**	Tissue enriched (testis)<br>Detected in some	Cancer enhanced (Bile duct cancer, Neuroblastoma)<br>Detected in some	Cancer enriched (testis cancer)<br>Detected in single	male and female infertility [44]	Male infertility<br>Infertility<br>Hereditary spastic paraplegia<br>Primary ovarian insufficiency	
**SPO11**	Tissue enriched (testis)<br>Detected in single	Not detected	Not detected	male and female infertility [45]	Infertility<br>Male infertility<br>Primary ovarian insufficiency	
**SPIDR**	Low tissue specificity<br>Detected in all	Low cancer specificity<br>Detected in all	Low cancer specificity<br>Detected in all	Male infertility, female subfertility [46]	Primary ovarian insufficiency	
**SWI5**	Tissue enhanced (testis)<br>Detected in all	Low cancer specificity<br>Detected in all	Low cancer specificity<br>Detected in all			
**TEX11**	Tissue enriched (pancreas)<br>Detected in some (testis)	Cancer enhanced (Bone cancer, Gastric cancer)<br>Detected in some	Cancer enhanced (renal cancer, testis cancer)<br>Detected in some	Male infertile and female are fertile [47]	Male infertility, Infertiity, Klinefelter	
**TRIP13**	Tissue enhanced (testis)<br>Detected in many	Low cancer specificity<br>Detected in all	Low cancer specificity<br>Low cancer specificity<br>Detected in many	Male and female infertility [48]	Cancer<br>Mosaic variegated aneuploidy syndrome	Mosaic variegated aneuploidy syndrome
**XRCC2**	Tissue enhanced (bone marrow)<br>Detected in many	Low cancer specificity<br>Detected in all	Low cancer specificity<br>Detected in many	embyronic lethal https://www.jax.org/strain/018146	Xeroderma pigmentosum<br>Ovarian cancer<br>Hereditary breast ovarian cancer syndrome<br>Anemia<br>Carcinoma	Breast cancer<br>Fanconi anemia<br>Primary ovarian insufficiency

Suplementary references

1. Herrán, Y.; Gutiérrez-Caballero, C.; Sánchez-Martín, M.; Hernández, T.; Viera, A.; Barbero, J.L.; de Álava, E.; de Rooij, D.G.; Suja, J.Á.; Llano, E.; et al. The Cohesin Subunit RAD21L Functions in Meiotic Synapsis and Exhibits Sexual Dimorphism in Fertility. *EMBO J.* **2011**, *30*, 3091–3105, doi:10.1038/emboj.2011.222.
2. Bannister, L.A.; Reinholdt, L.G.; Munroe, R.J.; Schimenti, J.C. Positional Cloning and Characterization of Mouse Mei8, a Disrupted Allelle of the Meiotic Cohesin Rec8. *Genes. N. Y. N 2000* **2004**, *40*, 184–194, doi:10.1002/gene.20085.
3. Revenkova, E.; Eijpe, M.; Heyting, C.; Hodges, C.A.; Hunt, P.A.; Liebe, B.; Scherthan, H.; Jessberger, R. Cohesin SMC1 Beta Is Required for Meiotic Chromosome Dynamics, Sister Chromatid Cohesion and DNA Recombination. *Nat. Cell Biol.* **2004**, *6*, 555–562, doi:10.1038/ncb1135.
4. Llano, E.; Gomez-H, L.; García-Tuñón, I.; Sánchez-Martín, M.; Caburet, S.; Barbero, J.L.; Schimenti, J.C.; Veitia, R.A.; Pendas, A.M. STAG3 Is a Strong Candidate Gene for Male Infertility. *Hum. Mol. Genet.* **2014**, *23*, 3421–3431, doi:10.1093/hmg/ddu051.
5. Caburet, S.; Arboleda, V.A.; Llano, E.; Overbeek, P.A.; Barbero, J.L.; Oka, K.; Harrison, W.; Vaiman, D.; Ben-Neriah, Z.; García-Tuñón, I.; et al. Mutant Cohesin in Premature Ovarian Failure. *N. Engl. J. Med.* **2014**, *370*, 943–949, doi:10.1056/NEJMoa1309635.
6. Daniel, K.; Lange, J.; Hached, K.; Fu, J.; Anastassiadis, K.; Roig, I.; Cooke, H.J.; Stewart, A.F.; Wassmann, K.; Jasin, M.; et al. Meiotic Homologue Alignment and Its Quality Surveillance Are Controlled by Mouse HORMAD1. *Nat. Cell Biol.* **2011**, *13*, 599–610, doi:10.1038/ncb2213.
7. Wojtasz, L.; Cloutier, J.M.; Baumann, M.; Daniel, K.; Varga, J.; Fu, J.; Anastassiadis, K.; Stewart, A.F.; Reményi, A.; Turner, J.M.A.; et al. Meiotic DNA Double-Strand Breaks and Chromosome Asynapsis in Mice Are Monitored by Distinct HORMAD2-Independent and -Dependent Mechanisms. *Genes Dev.* **2012**, *26*, 958–973, doi:10.1101/gad.187559.112.
8. Gómez-H, L.; Felipe-Medina, N.; Sánchez-Martín, M.; Davies, O.R.; Ramos, I.; García-Tuñón, I.; de Rooij, D.G.; Dereli, I.; Tóth, A.; Barbero, J.L.; et al. C14ORF39/SIX6OS1 Is a Constituent of the Synaptonemal Complex and Is Essential for Mouse Fertility. *Nat. Commun.* **2016**, *7*, 13298, doi:10.1038/ncomms13298.
9. Bolcun-Filas, E.; Hall, E.; Speed, R.; Taggart, M.; Grey, C.; de Massy, B.; Benavente, R.; Cooke, H.J. Mutation of the Mouse Syce1 Gene Disrupts Synapsis and Suggests a Link between Synaptonemal Complex Structural Components and DNA Repair. *PLoS Genet.* **2009**, *5*, e1000393, doi:10.1371/journal.pgen.1000393.
10. Bolcun-Filas, E.; Costa, Y.; Speed, R.; Taggart, M.; Benavente, R.; De Rooij, D.G.; Cooke, H.J. SYCE2 Is Required for Synaptonemal Complex Assembly, Double Strand Break Repair, and Homologous Recombination. *J. Cell Biol.* **2007**, *176*, 741–747, doi:10.1083/jcb.200610027.
11. Schramm, S.; Fraune, J.; Naumann, R.; Hernandez-Hernandez, A.; Höög, C.; Cooke, H.J.; Alsheimer, M.; Benavente, R. A Novel Mouse Synaptonemal Complex Protein Is Essential for Loading of Central Element Proteins, Recombination, and Fertility. *PLoS Genet.* **2011**, *7*, e1002088, doi:10.1371/journal.pgen.1002088.

12. de Vries, F.A.T.; de Boer, E.; van den Bosch, M.; Baarends, W.M.; Ooms, M.; Yuan, L.; Liu, J.-G.; van Zeeland, A.A.; Heyting, C.; Pastink, A. Mouse Sycp1 Functions in Synaptonemal Complex Assembly, Meiotic Recombination, and XY Body Formation. *Genes Dev.* **2005**, *19*, 1376–1389, doi:10.1101/gad.329705.
13. Yang, F.; De La Fuente, R.; Leu, N.A.; Baumann, C.; McLaughlin, K.J.; Wang, P.J. Mouse SYCP2 Is Required for Synaptonemal Complex Assembly and Chromosomal Synapsis during Male Meiosis. *J. Cell Biol.* **2006**, *173*, 497–507, doi:10.1083/jcb.200603063.
14. Yuan, L.; Liu, J.G.; Zhao, J.; Brundell, E.; Daneholt, B.; Höög, C. The Murine SCP3 Gene Is Required for Synaptonemal Complex Assembly, Chromosome Synapsis, and Male Fertility. *Mol. Cell* **2000**, *5*, 73–83, doi:10.1016/s1097-2765(00)80404-9.
15. Hamer, G.; Wang, H.; Bolcun-Filas, E.; Cooke, H.J.; Benavente, R.; Höög, C. Progression of Meiotic Recombination Requires Structural Maturation of the Central Element of the Synaptonemal Complex. *J. Cell Sci.* **2008**, *121*, 2445–2451, doi:10.1242/jcs.033233.
16. Papanikos, F.; Clément, J.A.J.; Testa, E.; Ravindranathan, R.; Grey, C.; Dereli, I.; Bondarieva, A.; Valerio-Cabrera, S.; Stanzione, M.; Schleiffer, A.; et al. Mouse ANKRD31 Regulates Spatiotemporal Patterning of Meiotic Recombination Initiation and Ensures Recombination between X and Y Sex Chromosomes. *Mol. Cell* **2019**, *74*, 1069-1085.e11, doi:10.1016/j.molcel.2019.03.022.
17. Holloway, J.K.; Morelli, M.A.; Borst, P.L.; Cohen, P.E. Mammalian BLM Helicase Is Critical for Integrating Multiple Pathways of Meiotic Recombination. *J. Cell Biol.* **2010**, *188*, 779–789, doi:10.1083/jcb.200909048.
18. Felipe-Medina, N.; Caburet, S.; Sánchez-Sáez, F.; Condezo, Y.B.; de Rooij, D.G.; Gómez-H, L.; Garcia-Valiente, R.; Todeschini, A.L.; Duque, P.; Sánchez-Martin, M.A.; et al. A Missense in HSF2BP Causing Primary Ovarian Insufficiency Affects Meiotic Recombination by Its Novel Interactor C19ORF57/BRME1. *eLife* **2020**, *9*, e56996, doi:10.7554/eLife.56996.
19. Robert, T.; Nore, A.; Brun, C.; Maffre, C.; Crimi, B.; Bourbon, H.-M.; de Massy, B. The TopoVIB-Like Protein Family Is Required for Meiotic DNA Double-Strand Break Formation. *Science* **2016**, *351*, 943–949, doi:10.1126/science.aad5309.
20. Ward, J.O.; Reinholdt, L.G.; Motley, W.W.; Niswander, L.M.; Deacon, D.C.; Griffin, L.B.; Langlais, K.K.; Backus, V.L.; Schimenti, K.J.; O'Brien, M.J.; et al. Mutation in Mouse Hei10, an E3 Ubiquitin Ligase, Disrupts Meiotic Crossing Over. *PLoS Genet.* **2007**, *3*, e139, doi:10.1371/journal.pgen.0030139.
21. Pittman, D.L.; Cobb, J.; Schimenti, K.J.; Wilson, L.A.; Cooper, D.M.; Brignull, E.; Handel, M.A.; Schimenti, J.C. Meiotic Prophase Arrest with Failure of Chromosome Synapsis in Mice Deficient for Dmc1, a Germline-Specific RecA Homolog. *Mol. Cell* **1998**, *1*, 697–705, doi:10.1016/s1097-2765(00)80069-6.
22. Schaetzlein, S.; Chahwan, R.; Avdievich, E.; Roa, S.; Wei, K.; Eoff, R.L.; Sellers, R.S.; Clark, A.B.; Kunkel, T.A.; Scharff, M.D.; et al. Mammalian Exo1 Encodes Both Structural and Catalytic Functions That Play Distinct Roles in Essential Biological Processes. *Proc. Natl. Acad. Sci. U. S. A.* **2013**, *110*, E2470-2479, doi:10.1073/pnas.1308512110.
23. Tsui, V.; Crismani, W. The Fanconi Anemia Pathway and Fertility. *Trends Genet.* **2019**, *35*, 199–214, doi:10.1016/j.tig.2018.12.007.

24. Guiraldelli, M.F.; Eyster, C.; Wilkerson, J.L.; Dresser, M.E.; Pezza, R.J. Mouse HFM1/Mer3 Is Required for Crossover Formation and Complete Synapsis of Homologous Chromosomes during Meiosis. *PLOS Genet.* **2013**, *9*, e1003383, doi:10.1371/journal.pgen.1003383.
25. Adelman, C.A.; Lolo, R.L.; Birkbak, N.J.; Murina, O.; Matsuzaki, K.; Horejsi, Z.; Parmar, K.; Borel, V.; Skehel, J.M.; Stamp, G.; et al. HELQ Promotes RAD51 Paralogue-Dependent Repair to Avert Germ Cell Loss and Tumorigenesis. *Nature* **2013**, *502*, 381–384, doi:10.1038/nature12565.
26. Hustedt, N.; Saito, Y.; Zimmermann, M.; Álvarez-Quilón, A.; Setiaputra, D.; Adam, S.; McEwan, A.; Yuan, J.Y.; Olivieri, M.; Zhao, Y.; et al. Control of Homologous Recombination by the HROB-MCM8-MCM9 Pathway. *Genes Dev.* **2019**, *33*, 1397–1415, doi:10.1101/gad.329508.119.
27. Pezza, R.J.; Voloshin, O.N.; Volodin, A.A.; Boateng, K.A.; Bellani, M.A.; Mazin, A.V.; Camerini-Otero, R.D. The Dual Role of HOP2 in Mammalian Meiotic Homologous Recombination. *Nucleic Acids Res.* **2014**, *42*, 2346–2357, doi:10.1093/nar/gkt1234.
28. Stanzione, M.; Baumann, M.; Papanikos, F.; Dereli, I.; Lange, J.; Ramlal, A.; Tränkner, D.; Shibuya, H.; de Massy, B.; Watanabe, Y.; et al. Meiotic DNA Break Formation Requires the Unsynapsed Chromosome Axis-Binding Protein IHO1 (CCDC36) in Mice. *Nat. Cell Biol.* **2016**, *18*, 1208–1220, doi:10.1038/ncb3417.
29. Kumar, R.; Bourbon, H.-M.; de Massy, B. Functional Conservation of Mei4 for Meiotic DNA Double-Strand Break Formation from Yeasts to Mice. *Genes Dev.* **2010**, *24*, 1266–1280, doi:10.1101/gad.571710.
30. Libby, B.J.; Reinholdt, L.G.; Schimenti, J.C. Positional Cloning and Characterization of Mei1, a Vertebrate-Specific Gene Required for Normal Meiotic Chromosome Synapsis in Mice. *Proc. Natl. Acad. Sci. U. S. A.* **2003**, *100*, 15706–15711, doi:10.1073/pnas.2432067100.
31. Luo, M.; Yang, F.; Leu, N.A.; Landaiche, J.; Handel, M.A.; Benavente, R.; La Salle, S.; Wang, P.J. MEIOB Exhibits Single-Stranded DNA-Binding and Exonuclease Activities and Is Essential for Meiotic Recombination. *Nat. Commun.* **2013**, *4*, 2788, doi:10.1038/ncomms3788.
32. Lutzmann, M.; Grey, C.; Traver, S.; Ganier, O.; Maya-Mendoza, A.; Ranisavljevic, N.; Bernex, F.; Nishiyama, A.; Montel, N.; Gavois, E.; et al. MCM8- and MCM9-Deficient Mice Reveal Gametogenesis Defects and Genome Instability Due to Impaired Homologous Recombination. *Mol. Cell* **2012**, *47*, 523–534, doi:10.1016/j.molcel.2012.05.048.
33. Baker, S.M.; Plug, A.W.; Prolla, T.A.; Bronner, C.E.; Harris, A.C.; Yao, X.; Christie, D.M.; Monell, C.; Arnheim, N.; Bradley, A.; et al. Involvement of Mouse Mlh1 in DNA Mismatch Repair and Meiotic Crossing Over. *Nat. Genet.* **1996**, *13*, 336–342, doi:10.1038/ng0796-336.
34. Lipkin, S.M.; Moens, P.B.; Wang, V.; Lenzi, M.; Shanmugarajah, D.; Gilgeous, A.; Thomas, J.; Cheng, J.; Touchman, J.W.; Green, E.D.; et al. Meiotic Arrest and Aneuploidy in MLH3-Deficient Mice. *Nat. Genet.* **2002**, *31*, 385–390, doi:10.1038/ng931.
35. Kneitz, B.; Cohen, P.E.; Avdievich, E.; Zhu, L.; Kane, M.F.; Hou, H.; Kolodner, R.D.; Kucherlapati, R.; Pollard, J.W.; Edelmann, W. MutS Homolog 4 Localization to

Meiotic Chromosomes Is Required for Chromosome Pairing during Meiosis in Male and Female Mice. *Genes Dev.* **2000**, *14*, 1085–1097.
36. Edelmann, W.; Cohen, P.E.; Kneitz, B.; Winand, N.; Lia, M.; Heyer, J.; Kolodner, R.; Pollard, J.W.; Kucherlapati, R. Mammalian MutS Homologue 5 Is Required for Chromosome Pairing in Meiosis. *Nat. Genet.* **1999**, *21*, 123–127, doi:10.1038/5075.
37. Mihola, O.; Trachtulec, Z.; Vlcek, C.; Schimenti, J.C.; Forejt, J. A Mouse Speciation Gene Encodes a Meiotic Histone H3 Methyltransferase. *Science* **2009**, *323*, 373–375, doi:10.1126/science.1163601.
38. Gómez-H, L.; Felipe-Medina, N.; Condezo, Y.B.; Garcia-Valiente, R.; Ramos, I.; Suja, J.A.; Barbero, J.L.; Roig, I.; Sánchez-Martín, M.; de Rooij, D.G.; et al. The PSMA8 Subunit of the Spermatoproteasome Is Essential for Proper Meiotic Exit and Mouse Fertility. *PLoS Genet.* **2019**, *15*, e1008316, doi:10.1371/journal.pgen.1008316.
39. Dai, J.; Voloshin, O.; Potapova, S.; Camerini-Otero, R.D. Meiotic Knockdown and Complementation Reveals Essential Role of RAD51 in Mouse Spermatogenesis. *Cell Rep.* **2017**, *18*, 1383–1394, doi:10.1016/j.celrep.2017.01.024.
40. Shu, Z.; Smith, S.; Wang, L.; Rice, M.C.; Kmiec, E.B. Disruption of MuREC2/RAD51L1 in Mice Results in Early Embryonic Lethality Which Can Be Partially Rescued in a P53(-/-) Background. *Mol. Cell. Biol.* **1999**, *19*, 8686–8693, doi:10.1128/MCB.19.12.8686.
41. Reynolds, A.; Qiao, H.; Yang, Y.; Chen, J.K.; Jackson, N.; Biswas, K.; Holloway, J.K.; Baudat, F.; de Massy, B.; Wang, J.; et al. RNF212 Is a Dosage-Sensitive Regulator of Crossing-over during Mammalian Meiosis. *Nat. Genet.* **2013**, *45*, 269–278, doi:10.1038/ng.2541.
42. Kumar, R.; Oliver, C.; Brun, C.; Juarez-Martinez, A.B.; Tarabay, Y.; Kadlec, J.; de Massy, B. Mouse REC114 Is Essential for Meiotic DNA Double-Strand Break Formation and Forms a Complex with MEI4. *Life Sci. Alliance* **2018**, *1*, e201800259, doi:10.26508/lsa.201800259.
43. Shi, B.; Xue, J.; Yin, H.; Guo, R.; Luo, M.; Ye, L.; Shi, Q.; Huang, X.; Liu, M.; Sha, J.; et al. Dual Functions for the SsDNA-Binding Protein RPA in Meiotic Recombination. *PLoS Genet.* **2019**, *15*, e1007952, doi:10.1371/journal.pgen.1007952.
44. La Salle, S.; Palmer, K.; O'Brien, M.; Schimenti, J.C.; Eppig, J.; Handel, M.A. Spata22, a Novel Vertebrate-Specific Gene, Is Required for Meiotic Progress in Mouse Germ Cells. *Biol. Reprod.* **2012**, *86*, 45, doi:10.1095/biolreprod.111.095752.
45. Romanienko, P.J.; Camerini-Otero, R.D. The Mouse Spo11 Gene Is Required for Meiotic Chromosome Synapsis. *Mol. Cell* **2000**, *6*, 975–987, doi:10.1016/s1097-2765(00)00097-6.
46. Huang, T.; Wu, X.; Wang, S.; Bao, Z.; Wan, Y.; Wang, Z.; Li, M.; Yu, X.; Lv, Y.; Liu, Z.; et al. SPIDR Is Required for Homologous Recombination during Mammalian Meiosis. *Nucleic Acids Res.* **2023**, gkad154, doi:10.1093/nar/gkad154.
47. Yang, F.; Gell, K.; van der Heijden, G.W.; Eckardt, S.; Leu, N.A.; Page, D.C.; Benavente, R.; Her, C.; Höög, C.; McLaughlin, K.J.; et al. Meiotic Failure in Male Mice Lacking an X-Linked Factor. *Genes Dev.* **2008**, *22*, 682–691, doi:10.1101/gad.1613608.
48. Li, X.C.; Schimenti, J.C. Mouse Pachytene Checkpoint 2 (Trip13) Is Required for Completing Meiotic Recombination but Not Synapsis. *PLoS Genet.* **2007**, *3*, e130, doi:10.1371/journal.pgen.0030130.
